# Hierarchical analyses of avian community biogeography in the Afromontane highlands

**DOI:** 10.21425/f5fbg51310

**Published:** 2021-11-01

**Authors:** Jacob C. Cooper

**Affiliations:** 1 University of Chicago Committee on Evolutionary Biology, 1025 E. 57th Street, Chicago, IL 60637, USA; 2 Division of Birds, Negaunee Integrative Research Center, Field Museum, Chicago, IL 60605, USA; 3 Current Address: University of Kansas Biodiversity Institute, 1345 Jayhawk Boulevard, Lawrence, KS 66045, USA

**Keywords:** Afromontane, Afromontane birds, cluster analyses, ecostructure, montane biogeography, vicariance

## Abstract

The Afromontane mountains are a complex series of highlands that have intermittently been connected by habitat corridors during climatic cycles, resulting in a mosaic of range disjunctions and allospecies complexes in the present day. Patterns of community relatedness between geographic regions are often determined through single-species analyses or spatial analyses of diversity and nestedness at the species level. To understand patterns of Afromontane community evolution and to assess the effects of taxonomy on our understanding of biogeographic patterns, I concatenated three lists of Afromontane bird taxa divided into five taxonomic hierarchies. These lists were converted into a presence-absence matrix across 42 montane regions and analyzed using a variety of clustering techniques based on a replicable coding pipeline. I used these lists and methods to determine patterns of relatedness between montane blocks, to assess the consistency with which biogeographic regions were recovered, and to shed light on the patterns of connectivity within the Afromontane region. My results reaffirm the distinctiveness of many biogeographic regions (e.g., the Cameroon Highlands) while also clarifying regional relationships and the presence of ‘transition zones’ between regions. Differences between lists illustrated how our understanding of taxonomy and distribution in the Afromontane highlands can also change our understanding of Afromontane biogeography. Most notably, I found evidence for an Expanded Eastern Arc that included the Eastern Arc Mountains and highlands in Malawi, Mozambique, and Zimbabwe. This study presents a rigorous yet easily adjustable pipeline for studying regional biogeography from multiple taxonomic perspectives using both traditional and novel approaches.

## Introduction

For hundreds of years, scientists have studied the ways in which montane communities are assembled and maintained (Rahbek et al. 2019). Within Africa, complex patterns of regional endemism and large range disjunctions within single species or between related taxa complicate our understanding of connectivity between extant montane habitats (Hall and Moreau 1970, Dowsett 1986, Bowie 2003, Linder et al. 2012). Many of these patterns of disjunction and local endemism appear to result from two complementary processes: (i) environmental changes that have allowed for ranges to expand and contract through time (e.g., the Turnover-Pulse Hypothesis *sensu* Vrba, 1993) and (ii) relatively stable climates in montane regions that allow for diversification while buffering against extinction (Prigogine 1987, Fjeldså and Bowie 2008, Fjeldså et al. 2011, Couvreur et al. 2020). An example of both phenomena for birds is the Phasianidae genus *Xenoperdix* (Svendsen et al., 1994), a relative of the Southeast Asian genus *Arborophila* (Hodgson, 1837). This relict genus has persisted in the Eastern Arc Mountains of Tanzania, where the complex regional geography and climatic cycling has promoted the differentiation of two geographically proximate (but allopatric) taxa that are often regarded as separate species (Dinesen et al. 1994, Bowie and Fjeldså 2005).

The distributions of African taxa lend credence to the hypothesis that climatic cycling has resulted in modern distributions of flora and fauna by way of ‘corridors’ of suitable climatic conditions that linked disparate montane blocks (White 1981, Dowsett 1986, Prigogine 1987, Kingdon 1989, Vrba 1993, Bowie 2003, Fjeldså et al. 2011, Couvreur et al. 2020, Cooper et al. 2021). The relative likelihood of vicariance vs. dispersal (or range expansion) during these climatic cycles varies with respect to species’ life history, morphology, migratory habits, and available habitat (Cracraft 1983, Bermingham et al. 1992). Within the Afromontane avifauna, different magnitudes of separation exist, with some related, allopatric populations belonging to well-differentiated superspecies spread across the continent (e.g., the arboreal members of the genus *Turdoides* (Cretzschmar 1926) [formerly placed in *Kupeornis* (Serle 1949)]) while others show no obvious differentiation across widely separated populations (e.g., *Ploceus insignis*; Sharpe 1891, Billerman et al. 2020). The consistency of overlap between Afromontane regions with respect to the distributions of species with varying levels of diversification suggest that similar colonization patterns led to modern distributions for a majority of taxa.

These resulting broad-scale patterns of distributional overlap of avian montane lineages in Africa have been used to develop hypotheses of biogeographic relationships for multiple taxonomic groups (Hall and Moreau 1970, Dowsett 1986, Kingdon 1989, Graham et al. 2005, Linder et al. 2012). Previous studies of relationships have used hierarchical cluster analysis (Graham et al. 2005, Linder et al. 2012), nestedness analyses (Cordeiro 1998), and other methods of comparing species composition, including the Simpson’s index (Simpson 1960), which takes into account the low species richness of satellite montane blocks (Dowsett 1986). These studies rely both on the widespread distributions of some taxa (e.g., *Ploceus insignis*) and on the distribution of ‘superspecies’ that consist of multiple allopatric taxa that are or appear to be closely related (Hall and Moreau 1970, Dowsett 1986). These studies have reinforced the distinctiveness of many Afromontane blocks, such as the Ethiopian Highlands, while reinforcing the apparent connections of others, such as the ties between the avifaunas of the Cameroon Line and the Lacustrine Rift (also known as the Albertine Rift). Many of these studies have also sought to determine the number of biogeographic regions that exist within the African continent. The methods for this determination vary, but often rely heavily on how results from hierarchical clustering analysis are interpreted and on knowledge of the flora and fauna. Past biogeographical assessments are further complicated by shifting taxonomy, as new Afromontane bird species are still being described *de novo* (Bowie and Fjeldså 2005, Voelker et al. 2010) and as existing taxonomic treatises change through the addition of genetic, morphological, or ecological information (Bowie et al. 2005, Bowie et al. 2016, Pearson and Turner 2017, Cooper et al. 2021).

**Figure 1.**
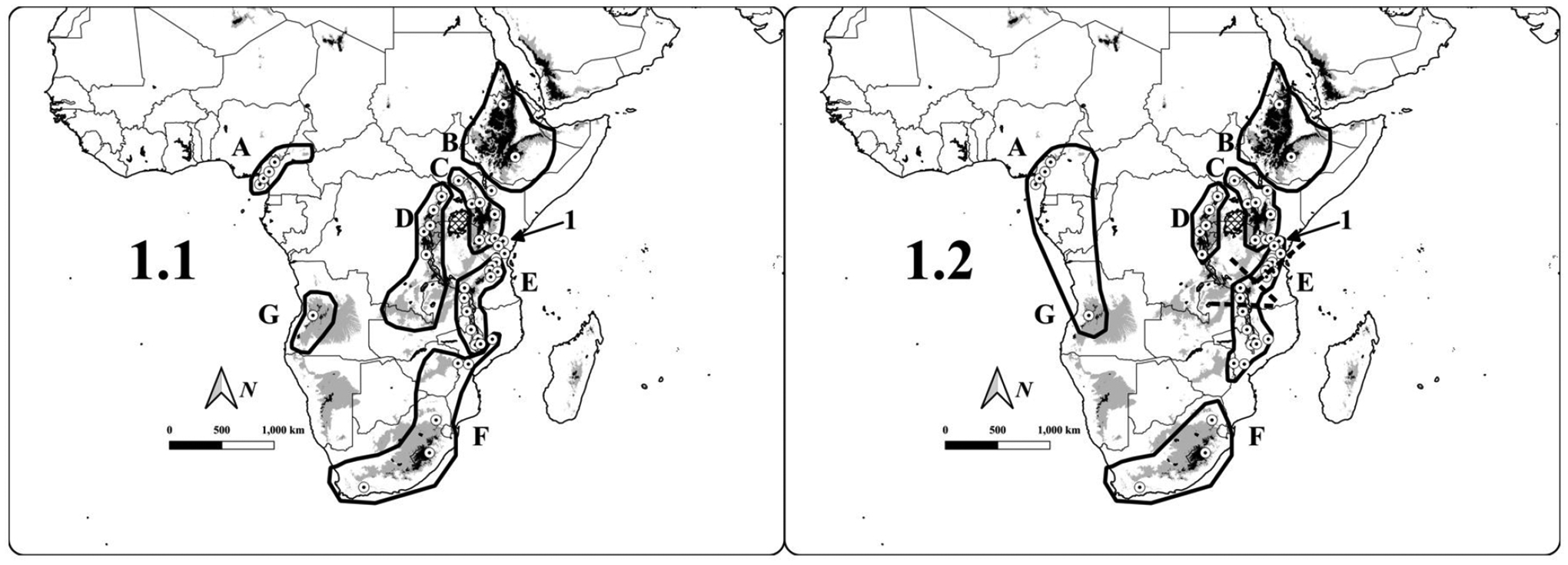
Maps of sub-Saharan Africa with study localities shown as white dots with black centers. Elevations of <1250 m are shown in white, elevations 1250–1750 m are shown in gray, and elevations of >1750 m are shown in black. Country boundaries (thin black lines) are superimposed and lakes are shown with cross-hatching. Approximated montane regions based on Dowsett (1986; Fig. 1.1 at left) and from the consensus dendrogram from the 2021 list (Fig 1.2 at right) are shown with thick black lines. Lettering refers to the following regions, with names corresponding to Dowsett (1986) and the 2021 list: **A**) Cameroon/Cameroon Highlands; **B**) Ethiopia/Ethiopian Highlands; **C**) Kenya/Kenya-Tanzania; **D**) East Congo/Lacustrine Rift; **E**) Tanganyika-Nyasa/Expanded Eastern Arc; **F**) Southeastern/Southern Great Escarpment; and **G**) Angola. For the 2021 list at right, subregions of the Expanded Eastern Arc are as follows, with approximate borders shown as bars across the biogeographic region: 1) Northern Eastern Arc (Taita Hills to Ukaguru/Rubeho); 2) Central Eastern Arc (Uluguru & Udzungwa); 3) Southern Highlands and Nyika; and 4) Southern Eastern Arc (alternatively, Malawi-Mozambique; Dedza-Salima to the Chimanimani Mountains). The annotation ‘**1**’ points to the Taita Hills, Pare Mountains, and Usambara Mountains, which were not placed in any specific region by Dowsett (1986) but group with the Expanded Eastern Arc in this study. Figure created in QGIS 3.18.2 & 3.20 (QGIS Development Team 2021) with elevation data from Amatulli et al. (2018) and map layers from NaturalEarthData (https://www.naturalearthdata.com/).

To address overall patterns of Afromontane biogeography in concert with changing taxonomy, I adapted a table of montane bird taxa from previous authors into a presence-absence matrix for 42 different montane regions (Figure 1; Table S1). The matrix is divided into different taxonomic hierarchies that reflect our understanding of Afromontane diversification at different taxonomic levels and, theoretically, with varying amounts of time (accumulated diversification) being used to define taxa. I analyze these data using a variety of clustering methods to determine 1) the relationships between different montane regions, 2) the robustness of the relationships between authors and methods, and 3) to identify ‘core’ montane regions and the ‘transitional’ zones between regions. Furthermore, I present the analysis pipeline in its entirety such that these analyses can be repeated in the future as our knowledge of the Afromontane avifauna improves.

## Materials and Methods

### Species dataset

I adapted a matrix of montane bird taxa from Dowsett (1986) and Bowie (2003) to a more recent taxonomy (Clements et al. 2019, 2021) and created a presence absence matrix of Afromontane birds for 51 sub-Saharan Afromontane sites (Table S1). These localities are generally well-known larger montane regions that are distributed throughout Sub-Saharan Africa, with some less species-rich ‘satellite ranges’ included as well. I expanded this list by including species with endemic montane populations that are more widespread outside of the study region (e.g., *Anthus similis*; Jerdon 1840), species that are found in lower-lying montane regions that are more xeric (e.g., *Columba oliviae* in the Somalian Highlands; Clarke 1918), species that are geographically restricted around montane regions (e.g., *Monticola rupestris*; Vieillot 1818), and species that are elevational migrants within specific montane regions (e.g., *Vanellus melanopterus*; Cretzschmar 1829). Hereafter, this list will be referred to as the ‘2021 list’ (Table S2). Thus, unlike Dowsett (1986) or Bowie (2003), the species I added to the montane list are affiliated with montane regions (as determined by habitat and distribution), but they are not restricted to specific elevations or habitat types. I referenced published habitat information and subspecific distributions from aggregated sources to create the complete presence absence matrix (Sinclair and Ryan 2010, Borrow and Demey 2014, Clements et al. 2019, Billerman et al. 2020, Clements et al. 2021), and I cross-referencing to two publicly accessible databases, eBird (https://ebird.org/, Sullivan et al. 2009, 2014) and Xeno-Canto (https://www.xeno-canto.org/, specifically Lambert 2017). These maps and databases, while professionally assembled and curated, can be less accurate than individually compiled species lists, published localities, vetted sightings, or georeferenced specimens (Ficetola et al. 2014). I opted for these resources because of their public availability and because the inclusion or omission of localized taxa had a minimal effect on cluster topology. Taxa not within the study area’s montane regions (e.g., *Sylvia lugens clara*; Meise 1933), taxa that migrate between montane regions latitudinally (e.g., *Sarothrura ayresi*; Gurney 1877), and taxa that are locally montane but occur broadly in lower elevations within the tropical latitudes (e.g., *Tauraco livingstonii*; Gray 1864) were excluded from all iterations of the analyses (Table S3). I excluded montane regions that had <34 species based on the combined species lists except for Mt. Gorongosa whose affiliations with other East African highlands are quite strong despite its low species richness, thus resulting in 42 regions for the analyses. This species richness threshold was used (i) to avoid the inclusion of less studied montane regions whose species list are more likely to be incomplete and (ii) to avoid clustering unrelated, species depauperate satellite montane regions (de Klerk et al. 2002).

I analyzed species’ distributions across montane regions using five taxonomic hierarchies (i.e., levels): Genus, Superspecies, Species, Group, and Subspecies. Taxonomic hierarchies and taxonomy implemented can affect the patterns recovered by scientists, and understanding the bias introduced by human classification is an important facet of biogeographical research (Fjeldså 2003). I derived Genus, Species, Group, and Subspecies from the eBird/Clements et al. (2019, 2021) checklist, wherein ‘Groups’ are used within the eBird taxonomy for related and/or similar and presumably related taxa. For example, *Campethera tullbergi* (Sjöstedt, 1892) possesses two phenotypically differentiable Groups, *tullbergi* and *taniolaema/hausburgi*, which have been considered as a monotypic *C. tullbergi* and polytypic *C. taniolaema* by some authors (Gill et al. 2021). Superspecies are derived from historical taxonomies and from known and/or presumed relationships between taxa (Billerman et al. 2020). I included this category to replicate the Superspecies category used by Hall and Moreau (1970). The Superspecies category has evolved with published genetic data highlighting allospecies relationships between populations formerly considered conspecific (e.g., the *Laniarius fuelleborni* complex; Reichenow 1900, Voelker et al. 2010). Many presumed superspecies are not grouped together due to ambiguities regarding their relationships (e.g., the arboreal members of the genus *Turdoides* [i.e., *Kupeornis*] are sometimes considered a single superspecies, but are considered separately herein; Hall and Moreau 1970, Dowsett 1986).

### Geographic similarity

I used the Jaccard index (*S*) to estimate the similarity in taxonomic composition among montane regions using the 2021 species list. The Jaccard index has a range from 0 (identical communities) to 1 (wholly different communities). I used the function ‘vegdist’ in the R package *vegan* to calculate the Jaccard index (Oksanen et al. 2019). An accompanying matrix of the great circle distances among montane regions was estimated using the approximate midpoint of each montane region or the location of the highest point in each montane region (https://www.google.com/maps/; https://www.wikipedia.org/; Table 1). I used the ‘distm’ function in the *geosphere* library to estimate great circle distances (Hijmans 2019). Lastly, I examined the correlation between the similarity and distance matrices using a Mantel test (Legendre and Legendre 2012), implemented using the ‘mantel’ function in the R package *vegan* (Oksanen et al. 2019).

### Clustering methods

I clustered montane regions based on similarities in taxonomic composition using *k*-means clustering (Forgy 1965, MacQueen 1967, Hartigan and Wong 1979, Lloyd 1982). This method partitions observations into *k* clusters such that the sum of squares in each cluster is minimized. The optimal number of clusters was determined using a gap-statistic (Tibshirani et al. 2001), implemented with the function ‘fviz_nbclust’ in the R package *factoextra* (Kassambara and Mundt 2020). For the optimal cluster size, group assignments for montane regions were saved for each dataset and for each taxonomic level.

I assessed the geographic patterns of taxonomic composition among montane regions using a hierarchical cluster analysis via the unweighted pair group method with arithmetic mean (UPGMA; Sokal & Michener 1958), implemented with the ‘hclust’ function in the R stats library (R Core Team 2021). This is a ‘bottom up’ method that groups the most similar regions at each step, thereby determining similarity in a stepwise fashion that can be visualized using dendrograms. I used the number of clusters from the *k*-means analysis to provide a non-biased estimate of the number of clusters for the hierarchical cluster analysis. However, I also visually identified breaks in the dendrograms to estimate the number of clusters, most notably for consensus dendrograms created from all taxonomic hierarchies for a particular list. Dendrograms were manipulated, analyzed, and plotted using the R packages *ape* (Paradis and Schleip 2019), *dendextend* (Galili 2015), *ggtree* (Yu et al. 2017, Yu et al. 2018, Yu 2020), *phytools* (Revell 2012), and *tidytree* (Yu 2021).

Lastly, I visualized the inherent geographic structure of taxonomic composition among montane regions using the R package *ecostructure* (White et al. 2019, White et al. 2021). This program emulates the genetic algorithm STRUCTURE (Hubisz et al. 2009) to parse presence-absence matrices into different geographic motifs. Similar to *k*-means clustering, this method can be applied across a spectrum of group sizes (*K*) to study the geographic structuring of communities. Unlike other methods employed herein, *ecostructure* also allows for viewing the ‘admixture’ between community motifs, allowing for clearer interpretation of connectivity between montane regions in a format that is not limited by group assignment or dendrogram formation. I performed iterations of *ecostructure* for *K* of 2–14 for each taxonomic hierarchy in the 2021 list, with particular attention paid to the number of groups determined from *k*-means clustering. This pipeline uses the *grid* function in the R stats library and functions in the R package *gridExtra* (Auguie 2017).

### General Coding Packages

All analyses were performed in the statistical software package R, versions 4.0.3 and 4.1.1 (R Core Team 2021). Data organization and visualizations were implemented using ColorBrewer 2.0 (https://colorbrewer2.org), ImageMagick (The ImageMagick Development Team 2021), and the R packages *data. table* (Dowle and Srinivasan 2019), *ggplot2* (Wickham 2016), *maps* (Becker et al. 2018), *rnaturalearth* (South 2017), *sf* (Pebesma 2018), *tidyverse* (Wickham et al. 2019), *viridis* (Garnier 2018), and *RcolorBrewer* (Neuwirth 2014).

## Results

For all taxonomic levels, the list derived from Dowsett (1986) had the fewest lineages represented, while Bowie (2003) had an intermediate and the 2021 list had the greatest number of lineages (Table 2). These lists possessed 261, 309, and 350 species, respectively.

**Figure 2.**
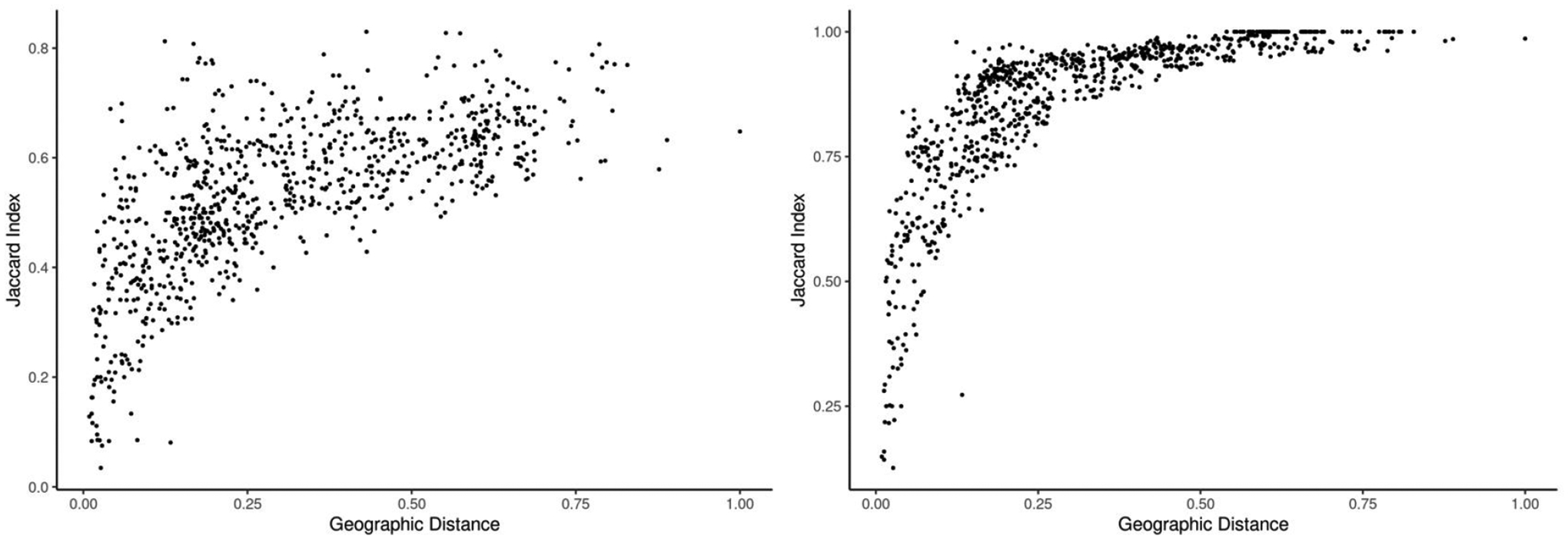
Jaccard index vs. geographic distance among Afromontane regions for the taxonomic levels of Genus (left) and subspecies (right) using the 2021 bird species list. Patterns of decreasing similarity with increasing geographic distance were consistent across taxonomic levels but the patterns and rate at which these changes occur increased with taxonomic specificity. Geographic distance is shown as a proportion of the greatest distance between any two points to facilitate viewing in the plot.

The Mantel tests demonstrated significant correlations between compositional similarity and geographic distance for all taxonomic levels (*P*<0.05). Compositional similarity declined with increasing geographic separation, although the magnitude and rate at which differences accumulated depended on taxonomic scale, with finer scales (e.g., subspecies) accumulated differences faster than coarser scales (Figure 2). Thus, while geographic proximity was correlated with compositional similarity, regions in the same geographic area can contain dissimilar taxonomic assemblages.

The optimal number of clusters for subdividing the Afromontane highlands varied by source list and by taxonomic level, with an average of 9 clusters for the Dowsett (1986) list, 10 clusters for the Bowie (2003) list, and 11 clusters for the 2021 list (Table 2). Across iterations of the cluster analysis, the exact number of clusters varied with the inclusion or exclusion of taxa (especially widespread taxa) and with the inclusion or exclusion of ‘satellite’ ranges with low species richness. The estimated number of clusters were most similar between lists at the species (mean *k* = 8.3, *σ* = 2.9) and subspecies levels (mean *k* = 10.3, *σ* = 2.1). The largest number of clusters (*k* = 20) were estimated at the Superspecies level using the Bowie (2003) list, and the smallest number of clusters (*k* = 3) were estimated at the Group level using the Bowie (2003) list.

Through all methods, several biogeographic regions were recovered that largely corresponded to those noted in the literature. The consensus dendrogram for all lists contained polytomies (Supplementary Appendix S1: 3.1, pp. 76–81), with a general consensus for eight major groups: 1) Ethiopian Highlands, 2) Lacustrine Rift (including the Lendu Plateau and Mt. Kabobo), 3) Cameroon Highlands, 4) Southern Great Escarpment, 5) Kenya-Tanzania Highlands (extending into Uganda [Mt. Elgon]), 6) Angola, 7) two relatively low-richness ‘transitional’ ranges peripheral to Kenya-Tanzania, the Imatong Mountains and the Southern Ethiopia/Northern Kenya Transitional Highlands, and 8) what I term the Expanded Eastern Arc (Supplementary Appendix S1: 3.5, p. 81).

**Figure 3.**
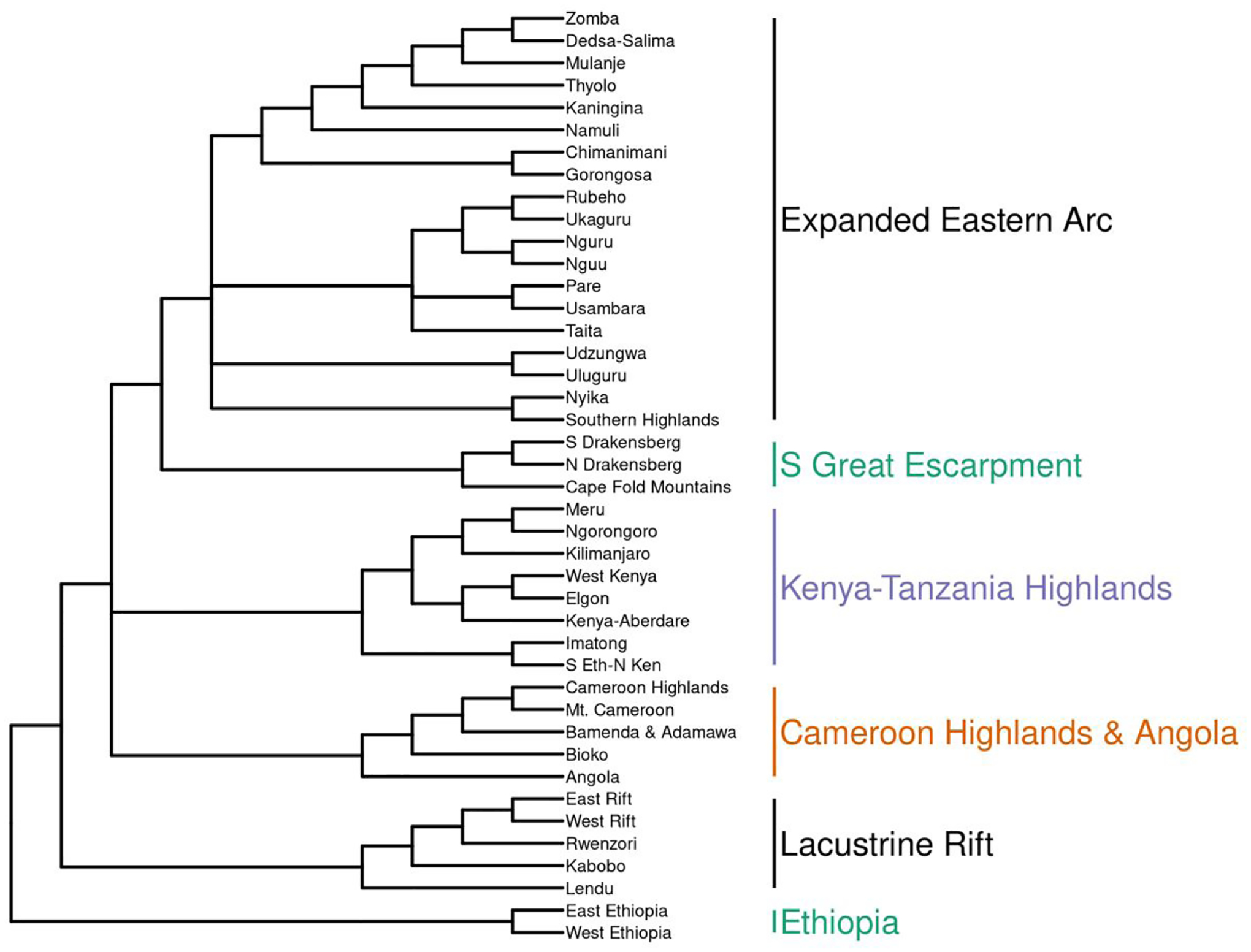
Consensus dendrogram across all taxonomic levels from the hierarchical cluster analysis of bird species assemblages among Afromontane regions using the 2021 species list.

**Figure 4.**
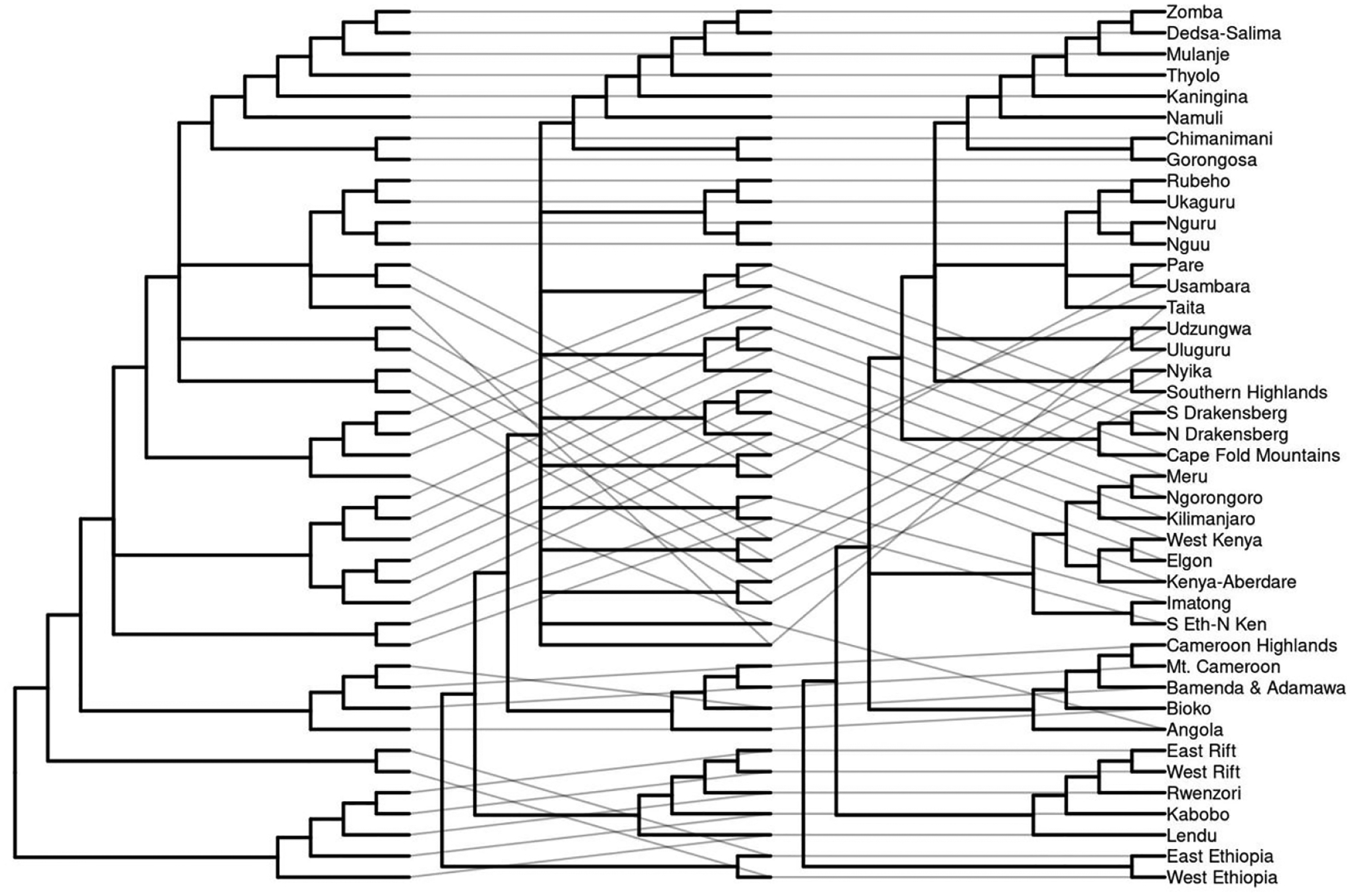
Consensus dendrograms across all taxonomic levels from the hierarchical cluster analysis of species assemblages among Afromontane regions using the species lists derived from Dowsett (1986; left), Bowie (2003, center), and the 2021 list (right). While relationships between individual montane regions were maintained within different lists, the relationships between larger biogeographic areas fluctuated between lists.

The Expanded Eastern Arc consists of a polytomy with four subgroups, namely A) a polytomy of the northern Eastern Arc Mountains from Taita Hills, Kenya to Rubeho and Ukaguru Mountains, Tanzania; B) the Udzungwa and Uluguru Mountains, Tanzania; C) Southern Highlands, Tanzania and Nyika, Malawi; D) Central Malawi south through Mozambique and Zimbabwe (with a southern terminus of the Chimanimani Mountains). Consensus trees for Dowsett (1986) and Bowie (2003) resulted in 7 and 14 groups respectively, with the Bowie list possessing a large polytomy for most of the Eastern and Southern African Highlands (Appendix S1: 3.3, p 78.). The 2021 list consensus has 6 major biogeographic regions: 1) Ethiopian highlands, 2) Lacustrine Rift; and a polytomy of 3) Kenya-Tanzania Highlands (including an outgroup of the Imatong Mountains and Southern Ethiopia/Northern Kenya Transitional Highlands); 4) the Southern Great Escarpment; 5) the Cameroon Highlands and Angola; and 6) the Expanded Eastern Arc stretching from Taita in the north to Chimanimani and Gorongosa in the south with a polytomy of 4 subgroups, with subgroup membership identical to the overall consensus dendrogram (Figure 1, Figure 3). The Bowie (2003) consensus list had the largest polytomy, whereas the Dowsett (1986) list and the 2021 list both possessed smaller polytomies regarding the Expanded Eastern Arc and the relationships between major Eastern and Southern African regions (Figure 4).

Within-region relationships were largely conserved through all sources, though the topology of the higher-order relationships within the dendrograms varied. The major subsets of the Kenya-Tanzania highlands, the Northern (Volcanic) Tanzania and Kenya-Uganda Highlands, however, are sister clusters within the Dowsett (1986) list and this study’s dendrogram analyses, hence their consideration as a single biogeographic entity (the Kenya-Tanzania Highlands) with two major subregions.

The removal of satellite regions with low species richness affected the number of clusters being recovered (especially with respect to *k*-means analyses), but had little effect on the overall topology of dendrograms within specific biogeographic regions. Transitional regions that possessed sufficient species richness to maintain their inclusion in these analyses became more ‘fluid’ in their position after the removal of other, minor satellite regions. Namely, the Imatong Mountains and the Southern Ethiopia/Northern Kenya Transitional Highlands (i.e., the fragmented highlands from the Mega region, Ethiopia to northern Kenya around Mt. Kulal) were often placed sister to each other and were not always linked to the nearby Kenya-Tanzania Highlands. Likewise, Angola fluctuated between different positions, reflecting its complicated biogeographic history with clear connections to multiple different montane regions. Many of these polytomies and the changing placement of specific montane regions within the dendrograms reflect patterns of historical connectivity and colonization between multiple Afromontane regions in the formation of communities. Transitional regions (i.e., regions at the flanking edges of ‘core’ biogeographic regions, such as the Southern Ethiopia/Northern Kenya Transitional Highlands) and regions that have been built by repeated colonization events from multiple montane blocks (i.e., Angola) therefore ‘jump’ between biogeographic clusters depending on the taxonomic level and species list being used.

The addition or subtraction of species from the presence-absence matrix had less of an effect on the topology of the dendrograms and regional *ecostructure* outputs, and instead affected the estimated number of clusters and higher-order relationships within the dendrograms. As mentioned, if not removed, satellite regions with low species richness clustered together in all methods. Widely disparate regions that share few (if any) species inherently shared many absences, leading to their aggregation.

**Figure 5.**
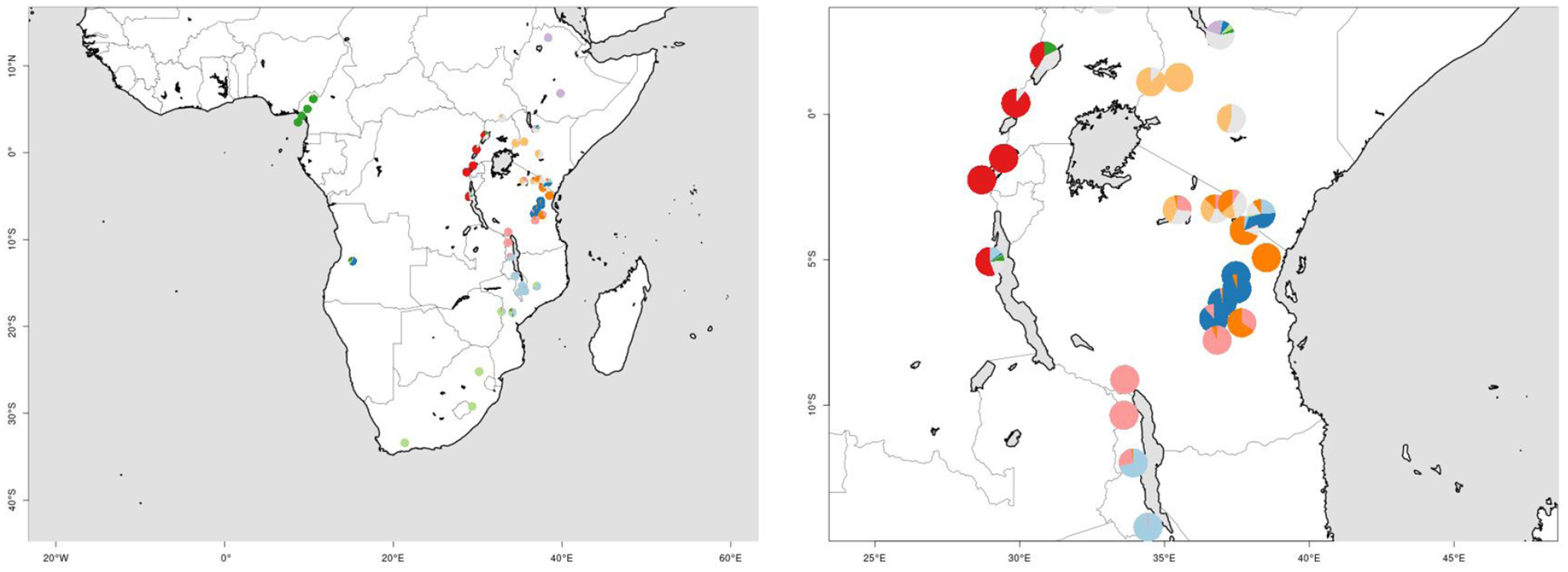
Assignments from the *ecostructure* analysis of Afromontane regions using *k* = 10, the ideal number of *k*-means groups determined for the 2021 species list at the Species level. Broad scale continental clusters are shown at left. At right, fine-scale analyses highlight the admixture present in transitional regions between biogeographic motifs, indicated by the presence of multiple colors in a given locality’s pie chart. The inset is centered on Tanzania, showing motif contribution to species communities at sites in the Lacustrine Rift (red), Kenya-Tanzania (light orange), Ethiopian Highlands (gray), and Expanded Eastern Arc subgroups (orange, blue, pink and light blue). Silver represents a motif that is predominant within the Kenya-Tanzania region, but also present in parts of adjacent montane regions (i.e., the Lacustrine Rift)

Similarly, the cluster analyses revealed connectivity between transitional regions and the main biogeographical regions they connect, demonstrating some of the same patterns as the dendrograms. Notably, this included the occasional grouping of the Lendu Plateau with the Cameroon Line rather than the adjacent Lacustrine Rift. Other vicariance or dispersal mediated connections between disparate montane regions were highlighted during different iterations of the clustering algorithms, such as the linkage between the Ethiopian Highlands and the Southern Great Escarpment (e.g., as illustrated by *Gypeatus barbatus* [Linnaeus 1758] and the genus *Heteromirafra* [Grant 1913]). The *ecostructure* analysis more clearly illustrated these patterns of connectivity, with many intermediary regions showing admixture from multiple biogeographic motifs (Figure 5). Transitional regions, such as the Taita Hills were shown to be the result of admixture from multiple biogeographical source populations rather than assigned to their own cluster or to one of their flanking regions.

## Discussion

This study presents a comprehensive, continent-scale analysis of Afromontane avian biogeography using multiple statistical techniques and species lists. By using different clustering algorithms, I demonstrate the ways in which highlands are connected and the ways in which complex biogeographical histories can confound straightforward interpretations of biogeographical clustering. This methodology has confirmed the stability of many local-scale patterns of biogeographic clustering with regards to regions such as the Lacustrine Rift and Southern Great Escarpment. Larger order clustering varies, however, with the regions elucidated here differing from those presented by Dowsett (1986) and those derived from the groupings presented by Bowie (2003). Previous categorization efforts relied primarily on a single clustering method applied to the distribution of superspecies (Hall and Moreau 1970), upon differing patterns of nestedness (Dowsett 1986, Cordeiro 1998), or the use of alternative clustering methods, most notably UPGMA (Graham et al. 2005, Linder et al. 2012). Here, I combine methods employed by previous authors with a new technique for visualizing community composition (*ecostructure*) that provides a novel perspective into the motifs contributing to transitional montane bird communities (White et al. 2019, White et al. 2021).

Perhaps the most similar previous analyses to this effort for Afromontane birds are those performed by Graham et al. (2005) and Dowsett (1986). Graham et al. (2005) employed extensive use of UPGMA dendrograms to understand the pattern and structure of 23 montane sites in the Cameroon Highlands. Their analyses showed varying patterns of regional clustering at different hierarchical scales, and varying patterns at different levels of species inclusion (i.e., complete communities vs. only regional endemics). Specific core areas were consistently recovered as ‘groups’ (e.g., the Adamawa Plateau), but other transitional or satellite highlands (e.g., Ngel-Nyaki) were difficult to place and often clustered with other satellite regions rather than adjacent core regions (Graham et al. 2005). Dowsett (1986) circumvented issues of low or lower species richness in satellite regions by employing the Simpson’s index (Simpson 1960) to assess “the proportion of the smaller avifauna that is shared by both” sites. Using this method and the number of endemic species, Dowsett (1986) illustrated the relatedness between major montane regions and elucidated the origin and relationships of these species while also noting many transitional and satellite regions.

Similar to Graham et al. (2005), I find that transitional and satellite regions are often difficult to place and that their position can shift depending on the species being included within the analyses. For many of these sites with low species richness, measures such as nestedness are more effective for elucidating biogeographic relationships, especially when cluster algorithms group montane regions based on absences rather than presences (Dowsett 1986). Dowsett (1986) recognized seven major montane regions: Cameroon Highlands, Angola, Southeastern (including the Southern Great Escarpment north to southern Malawi and central Mozambique), Tanganyika-Nyasa (the southern Eastern Arc and northern Malawi), East Congo (i.e., the Lacustrine Rift), Kenya (from the Imatong Mountains to northeastern Tanzania), and Ethiopia (Figure 1). Dowsett (1986) also considered the Usambara Mountains to be intermediary in composition and refrained from considering them part of either flanking region. Subsequent authors have recognized similar biogeographic regions, with deviances related primarily to the Eastern Arc Mountains, where work post-1986 has resulted in the discovery of multiple new taxa, clarifications of bird distributions, and a finer-scale understanding of regional community structure (Cordeiro 1998, Fjeldså et al. 2010, Billerman et al. 2020). Here, I found six major regions after the exclusion of satellite regions in the consensus dendrogram; however, these lists differ in their recognition of Angola as a distinct biogeographic entity (here grouped with the Cameroon Highlands), and in the assignation of mountains in East Africa between an Expanded Eastern Arc and Southern Great Escarpment rather than Dowsett’s (1986) transitional ranges around a Tanganyika-Nyasa group and a Southeastern Group (Figure 1).

Performing analyses at multiple taxonomic levels provides a clearer understanding of how community structuring varies across temporal scales and across human interpretations of diversity. While the amount of evolutionary time that contributes to Genus (and sometimes even Subspecific) levels of diversity varies, it serves as a proxy for varying amounts of accrued diversity through time. Assessing the Genus and Superspecies levels allows historical relationships to provide more weight for clustering models, whereas analyses of Group and Subspecies levels divided populations as finely as possible between individual montane regions to further elucidate recent relationships. The most consistent group numbers between authors is subject to vary with inclusion of satellite regions, but often includes the most intermediary hierarchy used, Species. The *k*-means cluster estimates are sensitive to the inclusion or exclusion of taxa, and thus should not be regarded as firm for the existing number of biogeographical regions. The UPGMA clustering method is more consistent in its overall topology, however, and large agreements for what constitutes a biogeographic region exist between datasets, though the relationships among these major areas are often unclear, especially based on the Bowie (2003) list. Furthermore, the inclusion or exclusion of taxa can affect the placement of transitional regions whose makeup is derived from multiple biogeographic regions.

Analyses of community composition using the *ecostructure* method are particularly illuminating for relationships between major biogeographic regions, especially for transitional and some satellite regions (Figure 5). The *ecostructure* method allows for more insight into the complex makeup of regions influenced by multiple biogeographic motifs (i.e., clusters). Regions such as the Taita Hills, located near the often difficult-to-classify Usambara Mountains (Dowsett 1986), show complex patterns of relatedness to adjacent biogeographic regions that are occluded by diversity metrics and traditional clustering techniques. Furthermore, adjusting levels of *K* within the *ecostructure* algorithm allows for finer and finer scale analyses of biogeographic breaks within particular regions, allowing for easier interpretations of community composition that defy ordinal classification. Results from *ecostructure* sometimes make it difficult to assign particular ranges to specific biogeographic motifs, but they can be enormously helpful in identifying transition regions of community admixture (White et al. 2021) and can more accurately reflect true patterns of non-uniform colonization between montane taxa, as evidenced by genetics and by varying levels of differentiation within single taxa and superspecies (Dowsett 1986, Vaz da Silva 2015, Billerman et al. 2020, Cooper et al. 2021). However, this study still cannot clarify the ambiguity regarding the effects of local extinction on species’ modern distributions and modern montane communities given that vicariant processes can result in parallel patterns to dispersal (e.g., in the case of *Gypeatus barbatus* in the Ethiopian Highlands and the Southern Great Escarpment; Zink et al. 2000, Billerman et al. 2020).

All of these results taken together help refine our understanding of the connectivity and clustering of mountain ranges, while also illuminating how our understanding of these relationships changes as our knowledge of the birds in the region improves. Using the 2021 list, I found that the different sections of the Eastern Arc Mountains, widely considered to be almost wholly limited to Tanzania (Burgess et al. 2007), may be more broadly considered to include all ranges between the Taita Hills of Kenya and the Chimanimani Mountains of Mozambique and Zimbabwe. Broader connections between these East Afromontane regions have been found for other organisms as well, such as the bird *Pogonicichla stellata* (Vieillot 1818, Bowie et al. 2006) and notably for the frog *Hyperolius substriatus* (Ahl 1931), which demonstrates connectivity between parts of the southern Malawi-Mozambique highlands (e.g., Mt. Mulanje) and the Eastern Arc Mountains of Tanzania (Lawson 2013). Several biogeographic connections have also been found across one of the main biogeographic breaks separating the Eastern Arc Mountains from the Southern Highlands (i.e., the Makambako Gap) in mammals (e.g., *Lophocebus*; Palmer 1903, Jones et al. 2005), chameleons (e.g., *Kinyongia*; Tillbury et al. 2006, Menegon et al. 2015), and vipers (e.g., *Atheris*; Cope 1862, Menegon et al. 2011). Different clustering methods suggest different ways of subsetting the Eastern Arc, and a combination of these methods confirms the presence of multiple distinct subregions and transitional flanking regions that parallels other biogeographic assessments within the region (Dowsett 1986, Cordeiro 1998). Likewise, connections found between highland regions in this study align with colonization routes hypothesized to exist using ecological niche models for other Afromontane birds and plants (Vaz da Silva 2015, Allen et al. 2021, Cooper et al. 2021).

Using these three species lists confirms the stability of many relationships between different highland regions while also illustrating how our interpretation of what constitutes a montane bird community can change how we view biogeographic relationships. Identical methods applied to these different species lists create a wide range of recommended cluster numbers while also resulting in surprising polytomies and relationship shifts as species were added or removed to the dataset. Most concurrence is in fine-scale biogeographic assessments, but some larger scale relationships (e.g., Kenya-Tanzania) are also largely recovered across source lists. Polytomies exist in every dataset and may never be fully resolved given the complex nature of colonization and connectivity between montane regions.

Our understanding of species limits and relationships continues to change (e.g., within the avian genus *Zosterops*; Linneaus 1766, Pearson and Turner 2017, Martins et al. 2020), and many montane taxa that are presently considered populations of lowland taxa (e.g., *Sheppardia gunningi alticola*; Fjeldså et al. 2000) may eventually be recognized as endemic montane species (Fjeldså et al. 2000). Afromontane species distribution knowledge also continues to improve as species like *Nesocharis ansorgei* (Hartert 1899) are discovered in regions where they were previously overlooked (Delhaye-Prat and Ikonga 2015, Mills and Pinto 2015). Thus, just as the results presented herein challenge some of those presented by previous studies, there is little doubt that future studies will further refine our understanding of biogeographic relationships among the Afromontane highlands.

## Supplementary Material

Supplemental **Appendix S1**. Code for the article.

TableS1 **Table S1**. Presence-absence matrix (PAM) of all Afromontane bird taxa used in this study, with annotations for source (e.g., Dowsett [1986]) and inclusion in statistical analyses.

TableS2 **Table S2**. Discrepancies between Afromontane bird taxa source lists used in this study.

TableS3 **Table S3**. Afromontane bird species excluded from these analyses, with reason for exclusion.

## Figures and Tables

**Table 1.** The Afromontane regions considered in the study ordered from Northwest to Southeast. Each region is listed with its name, region according to the 2021 list’s hierarchical cluster analysis (Figure 4), coordinates, and additional notes. In general, elevations conform to the highest point within a given highland, and coordinates refer to either the highest point or an approximate midpoint. Data here were gleaned primarily from Google Maps (https://google.com/maps) and Wikipedia (https://en.wikipedia.org). Note that this table excludes five regions from the Presence-Absence Matrix (PAM) due to their low species richness: the [Upper] Guinea Highlands (Côte d’Ivoire, Guinea-Conakry, Liberia & Sierra Leone), Monte Alen (Equatorial Guinea), Marungu (Democratic Republic of the Congo), Mahale (Tanzania), the North Somali Mountains (Somalia), the Djibouti Highlands (Djibouti), the Central African Plateau (Zambia), the Rondo Plateau (Tanzania), and the Mayombe Escarpment (Republic of the Congo).

Locality	Region	Longitude	Latitude	Elevation	Note
Bioko	Cameroon & Angola	8.7	3.5	3011	Pico Basilé
Mt. Cameroon	Cameroon & Angola	9.17	4.22	4040	
Cameroon Highlands	Cameroon & Angola	9.83	5.03	2411	Mt. Manengouba coordinates
Bamenda & Adamawa	Cameroon & Angola	10.52	6.2	3011	Mt. Oku [Kilum] coordinates
Lendu	Lacustrine Rift	30.86	2.01	2455	
West Rift	Lacustrine Rift	28.69	−2.25	3475	Mt. Kahuzi coordinates; includes Itombwe; high elev. for Itombwe given
Rwenzori	Lacustrine Rift	29.87	0.39	5109	Ngaliema [Mt. Stanley] coordinates
East Rift	Lacustrine Rift	29.45	−1.51	4507	Mt. Karisimba coordinates; includes Kibira-Nyungwe
Kabobo	Lacustrine Rift	28.97	−5.06	2700	
West Ethiopia	Ethiopia	38.37	13.24	4550	Ras Dashen coordinates
East Ethiopia	Ethiopia	39.82	6.83	4377	Tullu Dimtu coordinates
S Eth-N Ken	Kenya-Tanzania	36.92	2.73	2285	Mt. Kulal coordinates; includes some adjacent regions, such as Mega, Ethiopia
Imatong	Kenya-Tanzania	32.91	3.95	3187	Mt. Kinyeti coordinates
Elgon	Kenya-Tanzania	34.53	1.12	4321	
West Kenya	Kenya-Tanzania	35.5	1.27	3530	Includes several adjacent highlands; locality noted is Cherang’any [Cherangani] Hills
Kenya-Aberdare	Kenya-Tanzania	37.31	−0.15	5199	Mt. Kenya coordinates
Ngorongoro	Kenya-Tanzania	35.44	−3.28	3206	Oldeani (Mountain) coordinates
Meru	Kenya-Tanzania	36.75	−3.25	4562	
Kilimanjaro	Kenya-Tanzania	37.35	−3.08	5895	
Taita	Expanded Eastern Arc	38.33	−3.42	2228	Dawida Massif; estimated locality from Google.
Pare	Expanded Eastern Arc	37.75	−4	2463	Shengena Peak elevation; coordinates placed within range
Usambara	Expanded Eastern Arc	38.52	−4.93	2290	
Nguu	Expanded Eastern Arc	37.47	−5.55	1550	Birds may not be fully quantified in PAM
Nguru	Expanded Eastern Arc	37.5	−6	2400	
Ukaguru	Expanded Eastern Arc	37	−6.47	2250	
Rubeho	Expanded Eastern Arc	36.7	−7.02	2286	
Uluguru	Expanded Eastern Arc	37.67	−7.17	2630	
Udzungwa	Expanded Eastern Arc	36.82	−7.77	2579	
Southern Highlands	Expanded Eastern Arc	33.63	−9.12	2981	Mt. Rungwe
Nyika	Expanded Eastern Arc	33.6	−10.35	2605	
Kaningina	Expanded Eastern Arc	33.92	−11.99	1860	Kaningina Forest Reserve area
Dedza-Salima	Expanded Eastern Arc	34.43	−14.2	2000	Dedza-Salima Forest Reserve area
Zomba	Expanded Eastern Arc	35.29	−15.33	2087	
Thyolo	Expanded Eastern Arc	34.95	−16.05	1400	Michiru Mountain Conservation Area used as coordinates
Mulanje	Expanded Eastern Arc	35.59	−15.95	3002	
Namuli	Expanded Eastern Arc	37.03	−15.37	2419	
Gorongosa	Expanded Eastern Arc	34.11	−18.4	1863	
Chimanimani	Expanded Eastern Arc	32.84	−18.3	2592	Includes all East Highlands
N Drakensberg	S Great Escarpment	30.17	−25.21	2274	Steenkampsberg given for general highland area; Northern Drakensberg
S Drakensberg	S Great Escarpment	29.36	−29.2	3450	Mafadi used as coordinates
Cape Fold Mountains	S Great Escarpment	21.37	−33.4	2325	
Angola	Cameroon & Angola	15.17	−12.47	2620	

**Table 2.** The number of avian taxon units (*tc*) and the number of clusters from the *k*-means cluster analysis (*cl*) for three species lists (rows) and five taxonomic levels (columns) within the Afromontane highlands. The bottom row contains the number of clusters averaged across species lists and the last column contains the number of clusters averaged across taxonomic levels with the standard deviation (*SD*) shown in parentheses.

Source	Genus	Superspecies	Species	Group	Subspecies	Avg. Clusters
Dowsett (1986)	109 tc., 4 cl.	207 tc., 13 cl.	261 tc., 5 cl.	334 tc., 10 cl.	532 tc., 12 cl.	8.8 cl. (4.1)
Bowie (2003)	123 tc., 4 cl.	250 tc., 20 cl.	309 tc., 10 cl.	393 tc., 3 cl.	627 tc., 11 cl.	9.6 cl. (6.8)
2021 list	130 tc., 10 cl.	287 tc., 10 cl.	350 tc., 10 cl.	442 tc., 16 cl.	725 tc., 8 cl.	10.8 cl. (3.0)
*Avg*. k *cl*. (*SD*)	6 cl. (3.5)	14.3 cl. (5.1)	8.3 cl. (2.9)	9.6 cl. (6.5)	10.3 cl. (2.1)	9.73 cl.

## Highlights

- I used a hierarchical taxonomic framework to construct a continent-wide presence-absence matrix of bird occurrences to address hypotheses of the biogeographical relationships among Afromontane regions.
- Using classic techniques and novel techniques such as *ecostructure*, I highlight the position, relationship, and composition of ‘transitional regions’ between core montane areas.
- I demonstrate the stability of regional biogeographic assessments through time while providing clear evidence for how taxonomic and community considerations can affect higher-order relationships between major biogeographic areas.
- I support and expand upon the notion that many of Africa’s eastern and southern montane regions be considered part of the same biogeographic region, here termed the ‘Expanded Eastern Arc’.
- This study provides a robust framework for testing biogeographical hypotheses that can be replicated across other insular biogeographical systems.
