# Supplementary material for: Hierarchical analyses of avian community biogeography in the Afromontane highlands
Source: Front Biogeogr. Author manuscript; Available in PMC 2023 Feb 24. (PMC9956335; doi:10.21425/f5fbg51310)
Supplement: Supplemental — Appendix S1. Code for the article. [file NIHMS1832318-supplement-Supplemental.pdf]

Supplementary Material - Appendix S1

Code for 'Hierarchical analyses of avian community biogeography in the Afromontane highlands'

Frontiers of Biogeography - Issue 13.4

Jacob C. Cooper

13 October 2021

Contents

1.1 Introduction **4**

1.2 Setting up the code **4**
Required *R* Packages: . . . 4
Setting up the dataset: . . . 4
1.2.2 Remove Satellite Regions . . . 8

1.3 Taxa overview **10**

2.1 Introduction: Cluster Codes **10**

2.2 Clustering Code & Genus **14**

2.3 Superspecies Clusters **28**

2.4 Species Cluster **40**

2.5 Group **52**

2.6 Subspecies **64**

2.7 Number of groups across assessments **76**
KMeans . . . 76
Hierarchical Clustering . . . 77
Difference between Elbow and K-Means . . . 78

3.1 Introduction: Creating Consensus Trees **79**

3.2 Cooper Trees **80**

3.3 Bowie Trees **81**

3.4 Dowsett Trees **82**

3.5 All Sources **83**

4.1 Introduction: Plotting Trees **84**
Note on KMeans . . . 85

4.2 Phylogenetic Trees **85**
4.2.1 Genus . . . 85
4.2.2 Superspecies . . . 88
4.2.3 Species . . . 91
4.2.4 Group . . . 94
4.2.5 Subspecies . . . 97

4.3 Overall Consensus Tree **101**
3.1 Clustering for 2021 list . . . 103
3.2 Data with satellite regions, for comparison . . . 103

4.4 Cooper Consensus Plot **104**

5.1 Introduction: Ecostructure Part I: Setup and Initial Plots **108**

5.2 Genus assignment **115**
K = 2 118
K = 3 119
K = 4 119
K = 5 119
K = 6 119
K = 7 119
K = 8 119
K = 9 120
K = 10 120
K = 11 120
K = 12 120
K = 13 120
K = 14 120

6.1 Introduction: Ecostructure Part II **120**

6.2 Code Setup **120**

6.3 Superspecies assignment **122**
K = 2 122
K = 3 122
K = 4 122
K = 5 122
K = 6 122
K = 7 123
K = 8 123
K = 9 123
K = 10 123
K = 11 123
K = 12 123
K = 13 123
K = 14 123

1.1 Introduction

This exercise is designed to determine relationships between different Afromontane regions using different species lists and different taxonomic levels.

1.2 Setting up the code

I hide it here, but note that I declare my filepath for the rest of the document.

Required *R* Packages:

```
library(tidyverse)
```

```
## -- Attaching packages --------------------------------------- tidyverse 1.3.1 --
## v ggplot2 3.3.5     v purrr   0.3.4
## v tibble  3.1.4     v dplyr   1.0.7
## v tidyr   1.1.3     v stringr 1.4.0
## v readr   2.0.1     v forcats 0.5.1
## -- Conflicts ------------------------------------------ tidyverse_conflicts() --
## x dplyr::filter() masks stats::filter()
## x dplyr::lag()    masks stats::lag()
```

```
library(ggplot2)
```

Setting up the dataset:

Note here that I am reducing the presence-absence matrix down to the final presence-absence matrix used in the study (v2).

```
x=as.data.frame(read_csv(paste0(filepath,"TableS1-Species_PAM_v2.csv")))
```

```
## Rows: 825 Columns: 60
## -- Column specification --------------------------------------------------------
## Delimiter: ","
## chr  (8): From Bowie, From Dowsett, Exclude, Genus, Superspecies, Species, G...
## dbl (52): Clements, Upper Guinea Highlands, Bioko, Mt. Cameroon, Cameroon Hi...
##
## i Use `spec()` to retrieve the full column specification for this data.
## i Specify the column types or set `show_col_types = FALSE` to quiet this message.
```

```
summary(x)
```

```
##   From Bowie         From Dowsett         Exclude             Clements
##  Length:825         Length:825         Length:825         Min.   :  329
##  Class :character   Class :character   Class :character   1st Qu.:21949
##  Mode  :character   Mode  :character   Mode  :character   Median :23896
##                                                           Mean   :22705
##                                                           3rd Qu.:28639
##                                                           Max.   :31354
##
##     Genus           Superspecies         Species             Group
##  Length:825         Length:825         Length:825         Length:825
##  Class :character   Class :character   Class :character   Class :character
##  Mode  :character   Mode  :character   Mode  :character   Mode  :character
```

```
##
##
##
##
##   Subspecies         Upper Guinea Highlands     Bioko      Mt. Cameroon
##  Length:825         Min.   :1              Min.   :1     Min.   :1
##  Class :character   1st Qu.:1              1st Qu.:1     1st Qu.:1
##  Mode  :character   Median :1              Median :1     Median :1
##                     Mean   :1              Mean   :1     Mean   :1
##                     3rd Qu.:1              3rd Qu.:1     3rd Qu.:1
##                     Max.   :1              Max.   :1     Max.   :1
##                     NA's   :812            NA's   :789   NA's   :775
##  Cameroon Highlands Bamenda & Adamawa   Monte Alen      Lendu       West Rift
##  Min.   :1          Min.   :1         Min.   :1     Min.   :1     Min.   :1
##  1st Qu.:1          1st Qu.:1         1st Qu.:1     1st Qu.:1     1st Qu.:1
##  Median :1          Median :1         Median :1     Median :1     Median :1
##  Mean   :1          Mean   :1         Mean   :1     Mean   :1     Mean   :1
##  3rd Qu.:1          3rd Qu.:1         3rd Qu.:1     3rd Qu.:1     3rd Qu.:1
##  Max.   :1          Max.   :1         Max.   :1     Max.   :1     Max.   :1
##  NA's   :773        NA's   :761       NA's   :819   NA's   :756   NA's   :693
##     Rwenzori     East Rift       Kabobo       Marungu        Mahale
##  Min.   :1     Min.   :1     Min.   :1     Min.   :1     Min.   :1
##  1st Qu.:1     1st Qu.:1     1st Qu.:1     1st Qu.:1     1st Qu.:1
##  Median :1     Median :1     Median :1     Median :1     Median :1
##  Mean   :1     Mean   :1     Mean   :1     Mean   :1     Mean   :1
##  3rd Qu.:1     3rd Qu.:1     3rd Qu.:1     3rd Qu.:1     3rd Qu.:1
##  Max.   :1     Max.   :1     Max.   :1     Max.   :1     Max.   :1
##  NA's   :727   NA's   :705   NA's   :759   NA's   :792   NA's   :798
##  North Somali Mtns    Djibouti   West Ethiopia East Ethiopia  S Eth-N Ken
##  Min.   :1         Min.   :1     Min.   :1     Min.   :1     Min.   :1
##  1st Qu.:1         1st Qu.:1     1st Qu.:1     1st Qu.:1     1st Qu.:1
##  Median :1         Median :1     Median :1     Median :1     Median :1
##  Mean   :1         Mean   :1     Mean   :1     Mean   :1     Mean   :1
##  3rd Qu.:1         3rd Qu.:1     3rd Qu.:1     3rd Qu.:1     3rd Qu.:1
##  Max.   :1         Max.   :1     Max.   :1     Max.   :1     Max.   :1
##  NA's   :805       NA's   :821   NA's   :722   NA's   :732   NA's   :791
##     Imatong        Elgon       West Kenya  Kenya-Aberdare   Ngorongoro
##  Min.   :1     Min.   :1     Min.   :1     Min.   :1      Min.   :1
##  1st Qu.:1     1st Qu.:1     1st Qu.:1     1st Qu.:1      1st Qu.:1
##  Median :1     Median :1     Median :1     Median :1      Median :1
##  Mean   :1     Mean   :1     Mean   :1     Mean   :1      Mean   :1
##  3rd Qu.:1     3rd Qu.:1     3rd Qu.:1     3rd Qu.:1      3rd Qu.:1
##  Max.   :1     Max.   :1     Max.   :1     Max.   :1      Max.   :1
##  NA's   :762   NA's   :730   NA's   :717   NA's   :719    NA's   :741
##       Meru      Kilimanjaro      Taita          Pare        Usambara
##  Min.   :1     Min.   :1     Min.   :1     Min.   :1     Min.   :1
##  1st Qu.:1     1st Qu.:1     1st Qu.:1     1st Qu.:1     1st Qu.:1
##  Median :1     Median :1     Median :1     Median :1     Median :1
##  Mean   :1     Mean   :1     Mean   :1     Mean   :1     Mean   :1
##  3rd Qu.:1     3rd Qu.:1     3rd Qu.:1     3rd Qu.:1     3rd Qu.:1
##  Max.   :1     Max.   :1     Max.   :1     Max.   :1     Max.   :1
##  NA's   :741   NA's   :743   NA's   :782   NA's   :764   NA's   :744
##       Nguu         Nguru        Ukaguru       Rubeho       Uluguru
##  Min.   :1     Min.   :1     Min.   :1     Min.   :1     Min.   :1
```

```
##  1st Qu.:1     1st Qu.:1     1st Qu.:1     1st Qu.:1     1st Qu.:1
##  Median :1     Median :1     Median :1     Median :1     Median :1
##  Mean   :1     Mean   :1     Mean   :1     Mean   :1     Mean   :1
##  3rd Qu.:1     3rd Qu.:1     3rd Qu.:1     3rd Qu.:1     3rd Qu.:1
##  Max.   :1     Max.   :1     Max.   :1     Max.   :1     Max.   :1
##  NA's   :784   NA's   :779   NA's   :772   NA's   :774   NA's   :748
##     Udzungwa   Southern Highlands     Nyika       Kaningina    Dedza-Salima
##  Min.   :1     Min.   :1          Min.   :1     Min.   :1     Min.   :1
##  1st Qu.:1     1st Qu.:1          1st Qu.:1     1st Qu.:1     1st Qu.:1
##  Median :1     Median :1          Median :1     Median :1     Median :1
##  Mean   :1     Mean   :1          Mean   :1     Mean   :1     Mean   :1
##  3rd Qu.:1     3rd Qu.:1          3rd Qu.:1     3rd Qu.:1     3rd Qu.:1
##  Max.   :1     Max.   :1          Max.   :1     Max.   :1     Max.   :1
##  NA's   :737   NA's   :743        NA's   :744   NA's   :765   NA's   :777
##      Zomba          Thyolo       Mulanje        Namuli      Gorongosa
##  Min.   :1     Min.   :1     Min.   :1     Min.   :1     Min.   :1
##  1st Qu.:1     1st Qu.:1     1st Qu.:1     1st Qu.:1     1st Qu.:1
##  Median :1     Median :1     Median :1     Median :1     Median :1
##  Mean   :1     Mean   :1     Mean   :1     Mean   :1     Mean   :1
##  3rd Qu.:1     3rd Qu.:1     3rd Qu.:1     3rd Qu.:1     3rd Qu.:1
##  Max.   :1     Max.   :1     Max.   :1     Max.   :1     Max.   :1
##  NA's   :775   NA's   :775   NA's   :776   NA's   :784   NA's   :792
##   Chimanimani  N Drakensberg S Drakensberg Cape Fold Mountains
##  Min.   :1     Min.   :1     Min.   :1     Min.   :1
##  1st Qu.:1     1st Qu.:1     1st Qu.:1     1st Qu.:1
##  Median :1     Median :1     Median :1     Median :1
##  Mean   :1     Mean   :1     Mean   :1     Mean   :1
##  3rd Qu.:1     3rd Qu.:1     3rd Qu.:1     3rd Qu.:1
##  Max.   :1     Max.   :1     Max.   :1     Max.   :1
##  NA's   :771   NA's   :766   NA's   :757   NA's   :776
##  Central African Plateau (Zambia)     Angola    Rondo Plateau    Mayombe
##  Min.   :1                        Min.   :1     Min.   :1     Min.   :1
##  1st Qu.:1                        1st Qu.:1     1st Qu.:1     1st Qu.:1
##  Median :1                        Median :1     Median :1     Median :1
##  Mean   :1                        Mean   :1     Mean   :1     Mean   :1
##  3rd Qu.:1                        3rd Qu.:1     3rd Qu.:1     3rd Qu.:1
##  Max.   :1                        Max.   :1     Max.   :1     Max.   :1
##  NA's   :792                      NA's   :766   NA's   :817   NA's   :821
```

```r
x.1=x[,1:9]
x.2=x[,-c(1:9)]

x.2[is.na(x.2)]=0

x=cbind(x.1,x.2)

colSums(x[,-c(1:9)])
```

```
##          Upper Guinea Highlands                            Bioko
##                              13                               36
##                    Mt. Cameroon              Cameroon Highlands
##                              50                               52
##                Bamenda & Adamawa                      Monte Alen
##                              64                                6
##                           Lendu                       West Rift
```

```
##                                 69                                132
##                           Rwenzori                          East Rift
##                                 98                                120
##                             Kabobo                            Marungu
##                                 66                                 33
##                             Mahale                 North Somali Mtns
##                                 27                                 20
##                           Djibouti                     West Ethiopia
##                                  4                                103
##                      East Ethiopia                       S Eth-N Ken
##                                 93                                 34
##                            Imatong                              Elgon
##                                 63                                 95
##                         West Kenya                    Kenya-Aberdare
##                                108                                106
##                         Ngorongoro                               Meru
##                                 84                                 84
##                        Kilimanjaro                              Taita
##                                 82                                 43
##                               Pare                           Usambara
##                                 61                                 81
##                               Nguu                             Nguru
##                                 41                                 46
##                            Ukaguru                            Rubeho
##                                 53                                 51
##                            Uluguru                          Udzungwa
##                                 77                                 88
##                 Southern Highlands                              Nyika
##                                 82                                 81
##                          Kaningina                      Dedza-Salima
##                                 60                                 48
##                              Zomba                             Thyolo
##                                 50                                 50
##                            Mulanje                            Namuli
##                                 49                                 41
##                          Gorongosa                        Chimanimani
##                                 33                                 54
##                      N Drakensberg                     S Drakensberg
##                                 59                                 68
##                Cape Fold Mountains Central African Plateau (Zambia)
##                                 49                                 33
##                             Angola                     Rondo Plateau
##                                 59                                  8
##                            Mayombe
##                                  4
```

```
colnames(x)
```

```
##  [1] "From Bowie"                       "From Dowsett"
##  [3] "Exclude"                          "Clements"
##  [5] "Genus"                            "Superspecies"
##  [7] "Species"                          "Group"
##  [9] "Subspecies"                       "Upper Guinea Highlands"
## [11] "Bioko"                            "Mt. Cameroon"
## [13] "Cameroon Highlands"               "Bamenda & Adamawa"
```

```
## [15] "Monte Alen"                         "Lendu"
## [17] "West Rift"                          "Rwenzori"
## [19] "East Rift"                          "Kabobo"
## [21] "Marungu"                            "Mahale"
## [23] "North Somali Mtns"                  "Djibouti"
## [25] "West Ethiopia"                      "East Ethiopia"
## [27] "S Eth-N Ken"                        "Imatong"
## [29] "Elgon"                              "West Kenya"
## [31] "Kenya-Aberdare"                     "Ngorongoro"
## [33] "Meru"                               "Kilimanjaro"
## [35] "Taita"                              "Pare"
## [37] "Usambara"                           "Nguu"
## [39] "Nguru"                              "Ukaguru"
## [41] "Rubeho"                             "Uluguru"
## [43] "Udzungwa"                           "Southern Highlands"
## [45] "Nyika"                              "Kaningina"
## [47] "Dedza-Salima"                       "Zomba"
## [49] "Thyolo"                             "Mulanje"
## [51] "Namuli"                             "Gorongosa"
## [53] "Chimanimani"                        "N Drakensberg"
## [55] "S Drakensberg"                      "Cape Fold Mountains"
## [57] "Central African Plateau (Zambia)" "Angola"
## [59] "Rondo Plateau"                      "Mayombe"
```

```
x2=x

# reformat names to be hierarchical

x2$Superspecies=paste(x2$Genus,x2$Superspecies)
x2$Species=paste(x2$Superspecies,x2$Species)
x2$Group=paste(x2$Species,x2$Group)
x2$Subspecies=paste(x2$Group,x2$Subspecies)
```

1.2.2 Remove Satellite Regions

For this iteration, I am going to remove satellite regions that can conflate analyses and group depauperate regions together.

```
hist(colSums(x2[,10:ncol(x2)]),breaks = 15,
     main = "Histogram of Richness by Site",
     xlab = "Species richness")
```

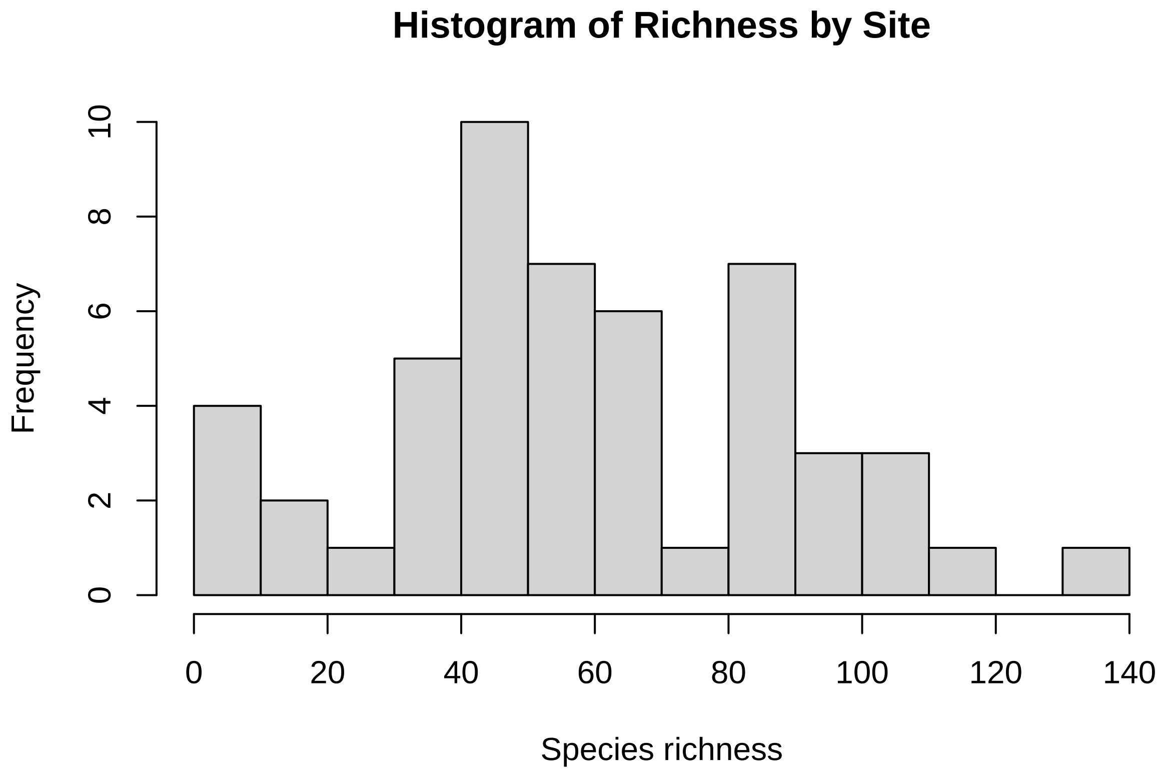

There appears to be a mostly normal distribution with a tail of regions with fewer than 20 localities. Which localities are these?

```
which(colSums(x2[,10:ncol(x2)])<34)
```

```
##          Upper Guinea Highlands                          Monte Alen
##                               1                                   6
##                         Marungu                              Mahale
##                              12                                  13
##               North Somali Mtns                            Djibouti
##                              14                                  15
##                       Gorongosa Central African Plateau (Zambia)
##                              43                                  48
##                   Rondo Plateau                             Mayombe
##                              50                                  51
```

These are the regions that are often grouped together as the *satellite regions* clade. Thus, we are removing them to improve the clustering ability and to better analyze the regions with better represented communities (that are thus more inferrable for relationships). **Note**: I am making an exception for Gorongosa because this region was classifiable, despite its more limited species community. This was also the only point that would not be classified as a 'satellite' region.

Note the following sites were removed from a previous iteration based on lack of confidence in species' lists:

1. Mahenge
2. Mbulu

```
x2=x2%>%select(-`Upper Guinea Highlands`,-`Monte Alen`,
               -Marungu,-Mahale,-`North Somali Mtns`,
```

```
               -Djibouti,-`Central African Plateau (Zambia)`,
               -`Rondo Plateau`,-Mayombe)

x2=x2[-which(rowSums(x2[,10:ncol(x2)])==0),]
# save alternate version without these localities
write_csv(x2,file = paste0(filepath,"TableS1_v2.csv"),col_names = T)
```

Now we need to reformat the metadata file as well.

```
locs=colnames(x2[,-c(1:9)])

meta=read_csv(paste0(filepath,"locality_metadata.csv"))
```

```
## Rows: 51 Columns: 6
```

```
## -- Column specification --------------------------------------------------------
## Delimiter: ","
## chr (3): Locality, Division, Note
## dbl (3): Longitude, Latitude, Elevation
```

```
##
## i Use `spec()` to retrieve the full column specification for this data.
## i Specify the column types or set `show_col_types = FALSE` to quiet this message.
```

```
meta2=meta[which(meta$Locality%in%locs),]

write_csv(meta2,file=paste0(filepath,"locality_metadata_v2.csv"))
```

1.3 Taxa overview

With the removal of the aforemention 'satellite' regions and other poorly sampled sites, we have the resulting number of species, with change denoted (if any). **These are the official numbers for this study**:

Source	Genus	Superspecies	Species	Group	Subspecies
Dowsett (1986)	109	207	261	334	532
Bowie (2003)	123	250	309	393	627
This study	130	287	350	442	725

2.1 Introduction: Cluster Codes

This exercise is designed to determine hypotheses of phylogenetic relationships between major biogeographic regions by means of assessing taxonomic relationships between all taxa known from these mountains. Taxa differ with regards to being described to Genera, species, and subspecies, and the hierarchical effects of these relationships sheds light on how similar populations are between different mountain ranges.

Here, we use `kmeans` clustering and `hclust` hierarchical clustering. I determine ideal group size of `kmeans` using the gap-statistic, and the ideal size for `hclust` using the more qualitative elbow method, with guidance from the `kmeans` group size.

```
library(tidyverse)
library(ggplot2)
library(vegan)
```

```
## Loading required package: permute
```

```
## Loading required package: lattice

## This is vegan 2.5-7
```

```
library(ape)
library(factoextra)
```

```
## Welcome! Want to learn more? See two factoextra-related books at https://goo.gl/ve3WBa
```

```
library(dendextend)
```

```
## Registered S3 method overwritten by 'dendextend':
##   method     from
##   rev.hclust vegan

##
## ---------------------
## Welcome to dendextend version 1.15.1
## Type citation('dendextend') for how to cite the package.
##
## Type browseVignettes(package = 'dendextend') for the package vignette.
## The github page is: https://github.com/talgalili/dendextend/
##
## Suggestions and bug-reports can be submitted at: https://github.com/talgalili/dendextend/issues
## Or contact: <tal.galili@gmail.com>
##
##  To suppress this message use:  suppressPackageStartupMessages(library(dendextend))
## ---------------------

##
## Attaching package: 'dendextend'

## The following objects are masked from 'package:ape':
##
##     ladderize, rotate

## The following object is masked from 'package:permute':
##
##     shuffle

## The following object is masked from 'package:stats':
##
##     cutree
```

```
x=as.data.frame(read_csv(paste0(filepath,"TableS1_v2.csv")))
```

```
## Rows: 732 Columns: 51

## -- Column specification --------------------------------------------------------
## Delimiter: ","
## chr  (8): From Bowie, From Dowsett, Exclude, Genus, Superspecies, Species, G...
## dbl (43): Clements, Bioko, Mt. Cameroon, Cameroon Highlands, Bamenda & Adama...

##
## i Use `spec()` to retrieve the full column specification for this data.
## i Specify the column types or set `show_col_types = FALSE` to quiet this message.
```

```
summary(x)
```

```
##   From Bowie        From Dowsett         Exclude             Clements
##  Length:732         Length:732         Length:732         Min.   :  329
```

```
##  Class :character   Class :character   Class :character   1st Qu.:21948
##  Mode  :character   Mode  :character   Mode  :character   Median :23890
##                                                           Mean   :22811
##                                                           3rd Qu.:28628
##                                                           Max.   :31354
##     Genus           Superspecies         Species            Group
##  Length:732         Length:732         Length:732         Length:732
##  Class :character   Class :character   Class :character   Class :character
##  Mode  :character   Mode  :character   Mode  :character   Mode  :character
##
##
##
##   Subspecies            Bioko          Mt. Cameroon     Cameroon Highlands
##  Length:732         Min.   :0.00000   Min.   :0.00000   Min.   :0.00000
##  Class :character   1st Qu.:0.00000   1st Qu.:0.00000   1st Qu.:0.00000
##  Mode  :character   Median :0.00000   Median :0.00000   Median :0.00000
##                     Mean   :0.04918   Mean   :0.06831   Mean   :0.07104
##                     3rd Qu.:0.00000   3rd Qu.:0.00000   3rd Qu.:0.00000
##                     Max.   :1.00000   Max.   :1.00000   Max.   :1.00000
##  Bamenda & Adamawa     Lendu          West Rift         Rwenzori
##  Min.   :0.00000   Min.   :0.00000   Min.   :0.0000   Min.   :0.0000
##  1st Qu.:0.00000   1st Qu.:0.00000   1st Qu.:0.0000   1st Qu.:0.0000
##  Median :0.00000   Median :0.00000   Median :0.0000   Median :0.0000
##  Mean   :0.08743   Mean   :0.09426   Mean   :0.1803   Mean   :0.1339
##  3rd Qu.:0.00000   3rd Qu.:0.00000   3rd Qu.:0.0000   3rd Qu.:0.0000
##  Max.   :1.00000   Max.   :1.00000   Max.   :1.0000   Max.   :1.0000
##    East Rift          Kabobo        West Ethiopia    East Ethiopia
##  Min.   :0.0000   Min.   :0.00000   Min.   :0.0000   Min.   :0.000
##  1st Qu.:0.0000   1st Qu.:0.00000   1st Qu.:0.0000   1st Qu.:0.000
##  Median :0.0000   Median :0.00000   Median :0.0000   Median :0.000
##  Mean   :0.1639   Mean   :0.09016   Mean   :0.1407   Mean   :0.127
##  3rd Qu.:0.0000   3rd Qu.:0.00000   3rd Qu.:0.0000   3rd Qu.:0.000
##  Max.   :1.0000   Max.   :1.00000   Max.   :1.0000   Max.   :1.000
##   S Eth-N Ken         Imatong            Elgon          West Kenya
##  Min.   :0.00000   Min.   :0.00000   Min.   :0.0000   Min.   :0.0000
##  1st Qu.:0.00000   1st Qu.:0.00000   1st Qu.:0.0000   1st Qu.:0.0000
##  Median :0.00000   Median :0.00000   Median :0.0000   Median :0.0000
##  Mean   :0.04645   Mean   :0.08607   Mean   :0.1298   Mean   :0.1475
##  3rd Qu.:0.00000   3rd Qu.:0.00000   3rd Qu.:0.0000   3rd Qu.:0.0000
##  Max.   :1.00000   Max.   :1.00000   Max.   :1.0000   Max.   :1.0000
##  Kenya-Aberdare     Ngorongoro          Meru         Kilimanjaro
##  Min.   :0.0000   Min.   :0.0000   Min.   :0.0000   Min.   :0.000
##  1st Qu.:0.0000   1st Qu.:0.0000   1st Qu.:0.0000   1st Qu.:0.000
##  Median :0.0000   Median :0.0000   Median :0.0000   Median :0.000
##  Mean   :0.1448   Mean   :0.1148   Mean   :0.1148   Mean   :0.112
##  3rd Qu.:0.0000   3rd Qu.:0.0000   3rd Qu.:0.0000   3rd Qu.:0.000
##  Max.   :1.0000   Max.   :1.0000   Max.   :1.0000   Max.   :1.000
##      Taita             Pare            Usambara           Nguu
##  Min.   :0.00000   Min.   :0.00000   Min.   :0.0000   Min.   :0.00000
##  1st Qu.:0.00000   1st Qu.:0.00000   1st Qu.:0.0000   1st Qu.:0.00000
##  Median :0.00000   Median :0.00000   Median :0.0000   Median :0.00000
##  Mean   :0.05874   Mean   :0.08333   Mean   :0.1107   Mean   :0.05601
##  3rd Qu.:0.00000   3rd Qu.:0.00000   3rd Qu.:0.0000   3rd Qu.:0.00000
##  Max.   :1.00000   Max.   :1.00000   Max.   :1.0000   Max.   :1.00000
```

```
##      Nguru           Ukaguru          Rubeho          Uluguru
##  Min.   :0.00000   Min.   :0.0000   Min.   :0.00000   Min.   :0.0000
##  1st Qu.:0.00000   1st Qu.:0.0000   1st Qu.:0.00000   1st Qu.:0.0000
##  Median :0.00000   Median :0.0000   Median :0.00000   Median :0.0000
##  Mean   :0.06284   Mean   :0.0724   Mean   :0.06967   Mean   :0.1052
##  3rd Qu.:0.00000   3rd Qu.:0.0000   3rd Qu.:0.00000   3rd Qu.:0.0000
##  Max.   :1.00000   Max.   :1.0000   Max.   :1.00000   Max.   :1.0000
##     Udzungwa      Southern Highlands     Nyika          Kaningina
##  Min.   :0.0000   Min.   :0.000      Min.   :0.0000   Min.   :0.00000
##  1st Qu.:0.0000   1st Qu.:0.000      1st Qu.:0.0000   1st Qu.:0.00000
##  Median :0.0000   Median :0.000      Median :0.0000   Median :0.00000
##  Mean   :0.1202   Mean   :0.112      Mean   :0.1107   Mean   :0.08197
##  3rd Qu.:0.0000   3rd Qu.:0.000      3rd Qu.:0.0000   3rd Qu.:0.00000
##  Max.   :1.0000   Max.   :1.000      Max.   :1.0000   Max.   :1.00000
##   Dedza-Salima         Zomba              Thyolo            Mulanje
##  Min.   :0.00000   Min.   :0.00000   Min.   :0.00000   Min.   :0.00000
##  1st Qu.:0.00000   1st Qu.:0.00000   1st Qu.:0.00000   1st Qu.:0.00000
##  Median :0.00000   Median :0.00000   Median :0.00000   Median :0.00000
##  Mean   :0.06557   Mean   :0.06831   Mean   :0.06831   Mean   :0.06694
##  3rd Qu.:0.00000   3rd Qu.:0.00000   3rd Qu.:0.00000   3rd Qu.:0.00000
##  Max.   :1.00000   Max.   :1.00000   Max.   :1.00000   Max.   :1.00000
##      Namuli          Gorongosa         Chimanimani       N Drakensberg
##  Min.   :0.00000   Min.   :0.00000   Min.   :0.00000   Min.   :0.0000
##  1st Qu.:0.00000   1st Qu.:0.00000   1st Qu.:0.00000   1st Qu.:0.0000
##  Median :0.00000   Median :0.00000   Median :0.00000   Median :0.0000
##  Mean   :0.05601   Mean   :0.04508   Mean   :0.07377   Mean   :0.0806
##  3rd Qu.:0.00000   3rd Qu.:0.00000   3rd Qu.:0.00000   3rd Qu.:0.0000
##  Max.   :1.00000   Max.   :1.00000   Max.   :1.00000   Max.   :1.0000
##  S Drakensberg    Cape Fold Mountains     Angola
##  Min.   :0.0000   Min.   :0.00000     Min.   :0.0000
##  1st Qu.:0.0000   1st Qu.:0.00000     1st Qu.:0.0000
##  Median :0.0000   Median :0.00000     Median :0.0000
##  Mean   :0.0929   Mean   :0.06694     Mean   :0.0806
##  3rd Qu.:0.0000   3rd Qu.:0.00000     3rd Qu.:0.0000
##  Max.   :1.0000   Max.   :1.00000     Max.   :1.0000
```

```
x[is.na(x)]=0
colSums(x[,-c(1:9)])
```

```
##               Bioko         Mt. Cameroon  Cameroon Highlands   Bamenda & Adamawa
##                  36                   50                  52                  64
##               Lendu           West Rift            Rwenzori           East Rift
##                  69                  132                  98                 120
##              Kabobo       West Ethiopia       East Ethiopia         S Eth-N Ken
##                  66                  103                  93                  34
##             Imatong               Elgon          West Kenya      Kenya-Aberdare
##                  63                   95                 108                 106
##          Ngorongoro                Meru         Kilimanjaro               Taita
##                  84                   84                  82                  43
##                Pare            Usambara                Nguu               Nguru
##                  61                   81                  41                  46
##             Ukaguru              Rubeho             Uluguru            Udzungwa
##                  53                   51                  77                  88
##  Southern Highlands               Nyika           Kaningina        Dedza-Salima
##                  82                   81                  60                  48
```

```
##                Zomba               Thyolo              Mulanje               Namuli
##                   50                   50                   49                   41
##            Gorongosa          Chimanimani       N Drakensberg        S Drakensberg
##                   33                   54                   59                   68
## Cape Fold Mountains               Angola
##                   49                   59
```

```
colnames(x)
```

```
##  [1] "From Bowie"          "From Dowsett"        "Exclude"
##  [4] "Clements"            "Genus"               "Superspecies"
##  [7] "Species"             "Group"               "Subspecies"
## [10] "Bioko"               "Mt. Cameroon"        "Cameroon Highlands"
## [13] "Bamenda & Adamawa"   "Lendu"               "West Rift"
## [16] "Rwenzori"            "East Rift"           "Kabobo"
## [19] "West Ethiopia"       "East Ethiopia"       "S Eth-N Ken"
## [22] "Imatong"             "Elgon"               "West Kenya"
## [25] "Kenya-Aberdare"      "Ngorongoro"          "Meru"
## [28] "Kilimanjaro"         "Taita"               "Pare"
## [31] "Usambara"            "Nguu"                "Nguru"
## [34] "Ukaguru"             "Rubeho"              "Uluguru"
## [37] "Udzungwa"            "Southern Highlands"  "Nyika"
## [40] "Kaningina"           "Dedza-Salima"        "Zomba"
## [43] "Thyolo"              "Mulanje"             "Namuli"
## [46] "Gorongosa"           "Chimanimani"         "N Drakensberg"
## [49] "S Drakensberg"       "Cape Fold Mountains" "Angola"
```

```
x2=x

# reformat names to be hierarchical

x2$Superspecies=paste(x2$Genus,x2$Superspecies)
x2$Species=paste(x2$Superspecies,x2$Species)
x2$Group=paste(x2$Species,x2$Group)
x2$Subspecies=paste(x2$Group,x2$Subspecies)
```

2.2 Clustering Code & Genus

```
# test variables for coding
level="Genus"
ncluster=9
hcluster=9
xdata=x2
author="Cooper"

# removed ncluster variable
# now determines best group number

clustertaxa=function(level,ncluster=NULL,hcluster=NULL,xdata,author){

  if(is.null(ncluster)==T){ncluster=5}
  if(is.null(hcluster)==T){hcluster=5}

  x3=xdata %>%
```

```r
  filter(Exclude!="Exclude") %>%
  select(-`From Bowie`,-`From Dowsett`,-Exclude,-Clements)

xnames=x3[,which(colnames(x3)==level)]

x4=x3%>%select(-Superspecies,-Genus,
               -Species,-Group,-Subspecies)

col.x=colnames(x4)
x4=as.data.frame(unclass(t(x4)))

colnames(x4)=xnames

for(i in 1:ncol(x4)){
  x4[,i]=as.numeric(as.character(x4[,i]))
}

u.names=unique(xnames)

for(i in 1:length(u.names)){
  target=u.names[i]
  if(sum(colnames(x4)==target)<=1){
    index=which(colnames(x4)==target)
    nu.x=x4[,c(index,index)]
    if(i==1){
      x6=nu.x
    }else{
      x6=cbind(x6,nu.x[,1])
    }
    if(i==2){
      x6=x6[,-2]
    }
  }
  if(sum(colnames(x4)==target)>1){
    xx=x4[,which(colnames(x4)==target)]
    nu.x=rowSums(xx)
    nu.x[nu.x>1]=1
    if(i==1){
      x6=nu.x
    }else{
      x6=cbind(x6,nu.x)
    }
    if(i==2){
      x6=x6[,-2]
    }
  }
}
colnames(x6)=u.names

# manual plot for kmeans

#wss=(nrow(x6)-1)*sum(apply(x6,2,var))
#for(v in 2:40){
```

```
#  wss[v]=sum(kmeans(x6,centers=v)$withinss)
#}

#plot(1:40,wss,type="b",xlab="Number of Clusters",
#   ylab="Within groups sum of squares")

set.seed(123)

# ncluster.det=which(wss==min(wss))

# defined from above plot

clust.x=hclust(dist(x6),method="average")

wss=(nrow(x6)-1)*sum(apply(x6,2,var))
x.cut=cutree(clust.x,2:40)

for(v in 2:30){ # reducing number to be plotted
  x.ssq=aggregate(x6,by=list(x.cut[,v]),function(x){sum(scale(x,scale=F)^2)})
  ssq=rowSums(x.ssq[,-1])
  TSS=sum(x.ssq[,-1])
  wss[v]=TSS
}

# use hcluster here as a comparison to the kmeans clusters

plot(x=1:30,y=wss,pch=19,type='b',xlab="Number of Clusters",
   ylab="Within groups sum of squares",main="H-Clust")
abline(v=hcluster)

plot(clust.x,main="Elbow Clust")
rect.hclust(clust.x,k=hcluster)

plot(clust.x,main="#K Clust")
rect.hclust(clust.x,k=ncluster)

x.tree=as.phylo(clust.x)

write.tree(x.tree,file=paste0(filepath,"hclust_",level,"_",author,".tre"))

#for(k in 1:nclusters){
#  xclust=kmeans(x6,nclusters[k],nstart=20)
#  return(xclust)
#}

print(fviz_nbclust(x6,kmeans,nstart=2,method="gap_stat",
             nboot=100,k.max=30)+
  labs(subtitle = "Kmeans: Gap Statistic"))

xclust=kmeans(x=x6,centers=ncluster)
assignments=as.data.frame(xclust$cluster)
write.csv(assignments,
          paste0(filepath,"clusters_",level,
```

```
                    "_K",ncluster,"_",author,".csv"),
            row.names = T,quote = F)
}
```

```
# prepare variables for tests
level="Superspecies"
#nclusters=5

# ensure we are excluding problem

xdata=x2 %>% filter(Exclude==0)
bowie.data=xdata%>%filter(`From Bowie`!=0)
dowsett.data=xdata%>%filter(`From Dowsett`!=0)
```

```
# my data

clustertaxa(level="Genus",xdata=xdata,
            ncluster=10,hcluster=6,
            author="Cooper")
```

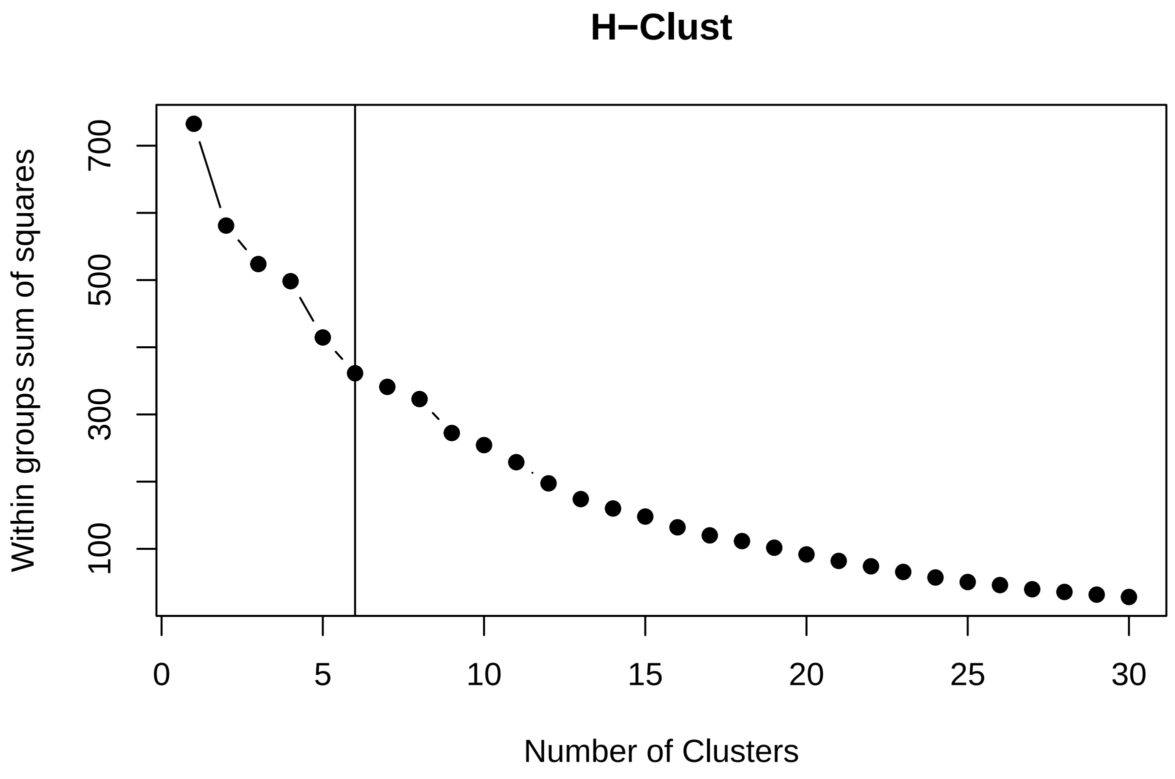

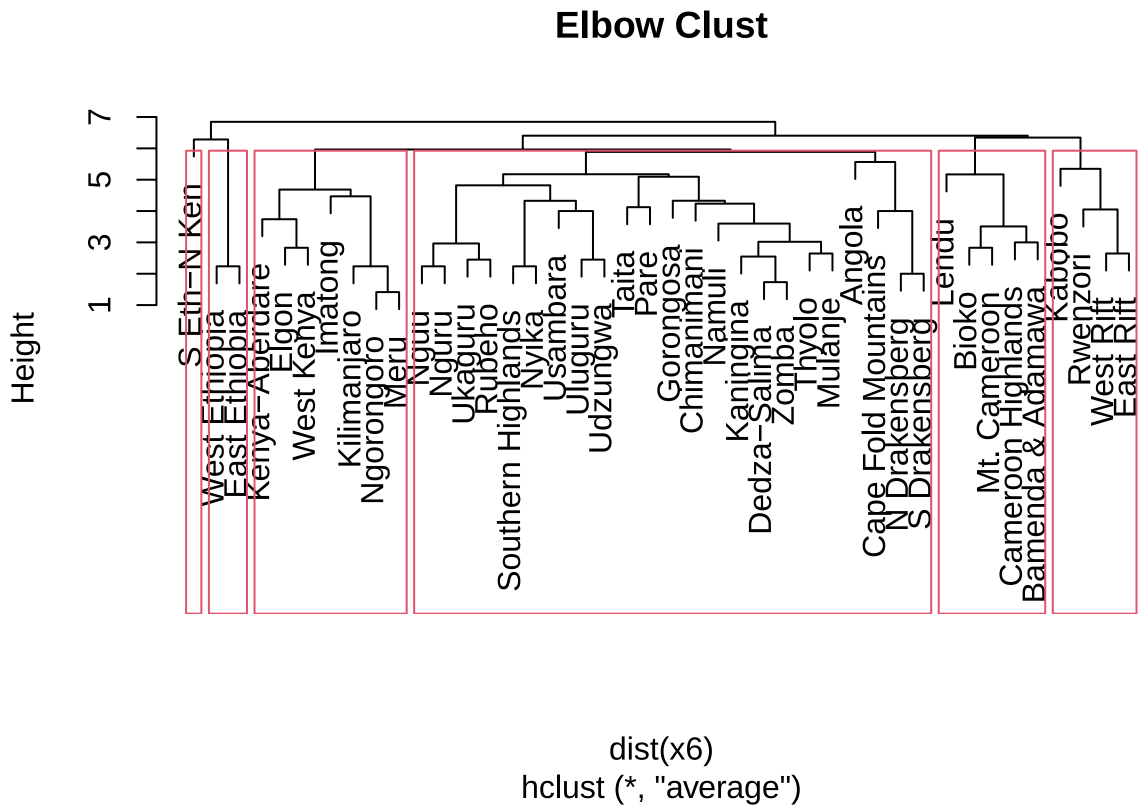
Elbow Clust
Height
1
3
5
7
S Eth−N Ken
West Ethiopia
East Ethiopia
Kenya−Aberdare
Elgon
West Kenya
Imatong
Kilimanjaro
Ngorongoro
Meru
Nguu
Nguru
Ukaguru
Rubeho
Southern Highlands
Nyika
Usambara
Uluguru
Udzungwa
Taita
Pare
Gorongosa
Chimanimani
Namuli
Kaningina
Dedza−Salima
Zomba
Thyolo
Mulanje
Angola
Cape Fold Mountains
N Drakensberg
S Drakensberg
Lendu
Bioko
Mt. Cameroon
Cameroon Highlands
Bamenda & Adamawa
Kabobo
Rwenzori
West Rift
East Rift
dist(x6)
hclust (*, "average")

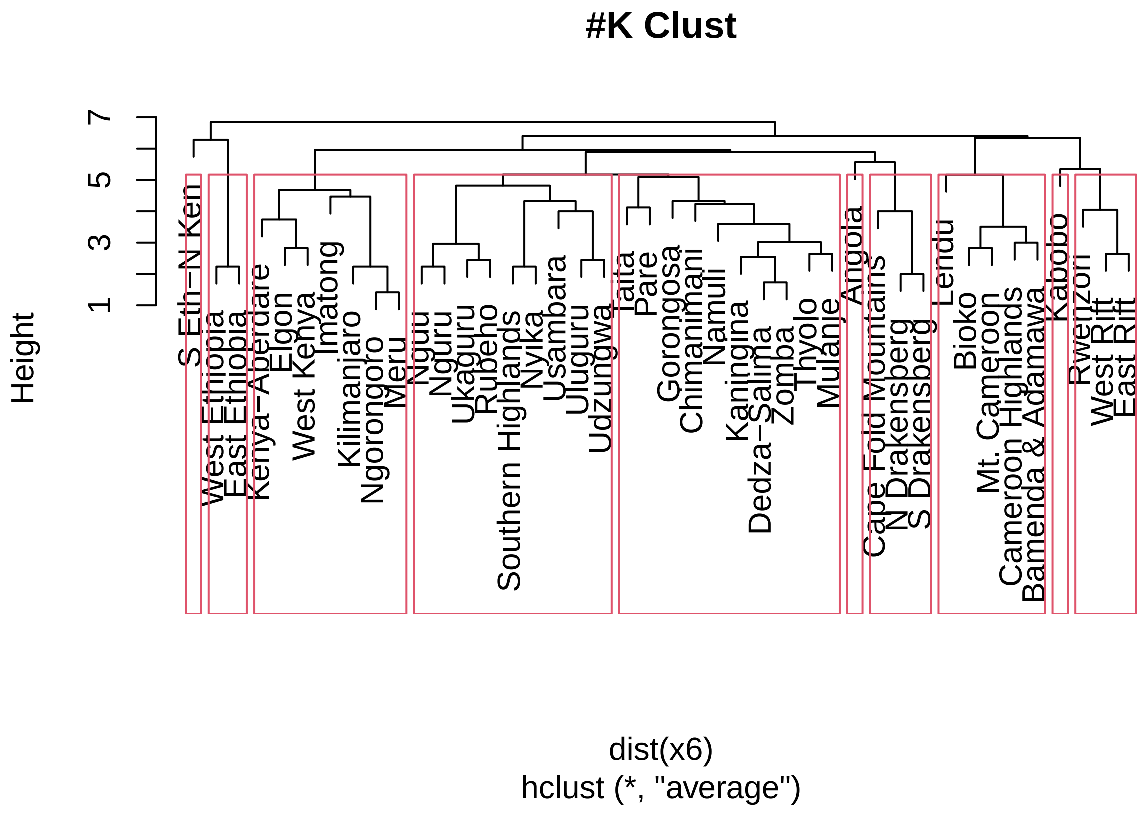
#K Clust
Height
1
3
5
7
S Eth−N Ken
West Ethiopia
East Ethiopia
Kenya−Aberdare
Elgon
West Kenya
Imatong
Kilimanjaro
Ngorongoro
Meru
Nguu
Nguru
Ukaguru
Rubeho
Southern Highlands
Nyika
Usambara
Uluguru
Udzungwa
Taita
Pare
Gorongosa
Chimanimani
Namuli
Kaningina
Dedza−Salima
Zomba
Thyolo
Mulanje
Angola
Cape Fold Mountains
N Drakensberg
S Drakensberg
Lendu
Bioko
Mt. Cameroon
Cameroon Highlands
Bamenda & Adamawa
Kabobo
Rwenzori
West Rift
East Rift
dist(x6)
hclust (*, "average")

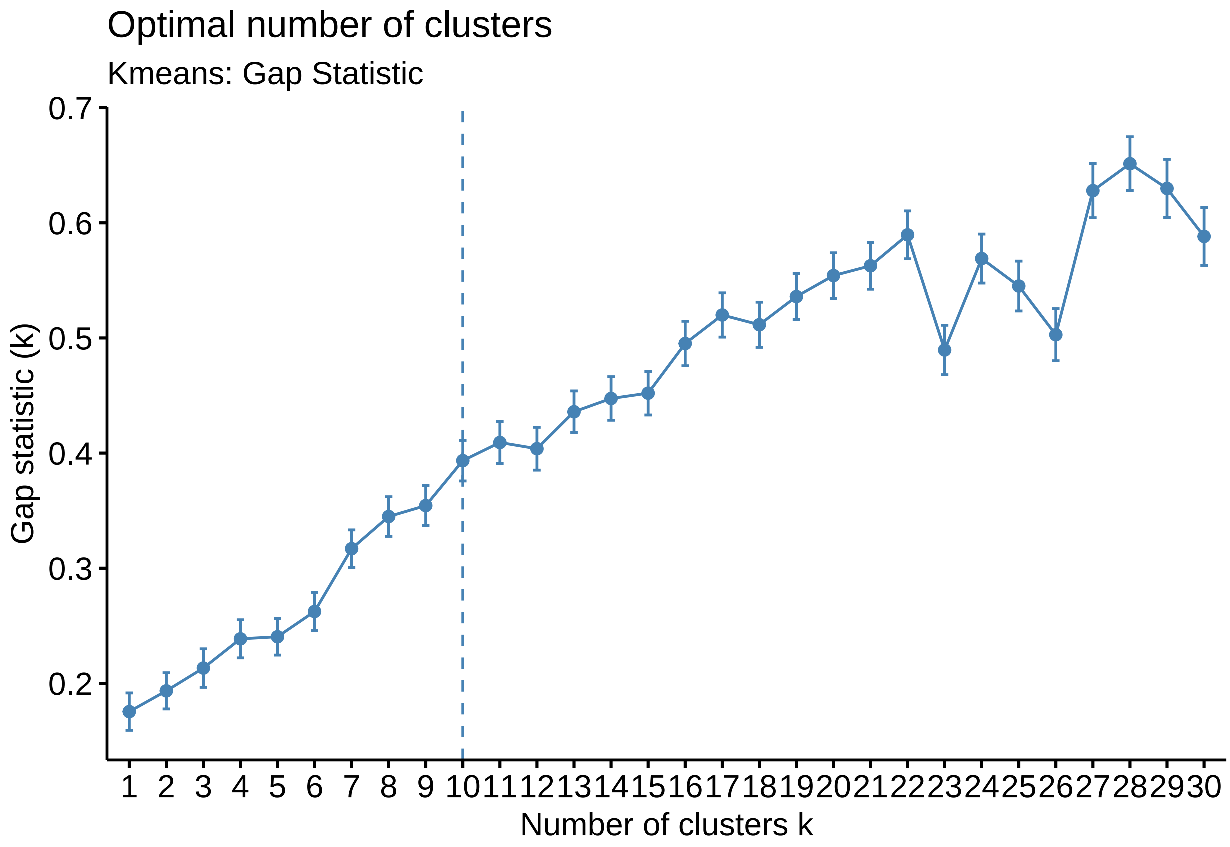


```
# Bowie data

clustertaxa(level="Genus",xdata=bowie.data,
            ncluster=4,hcluster=7,
            author="Bowie")
```

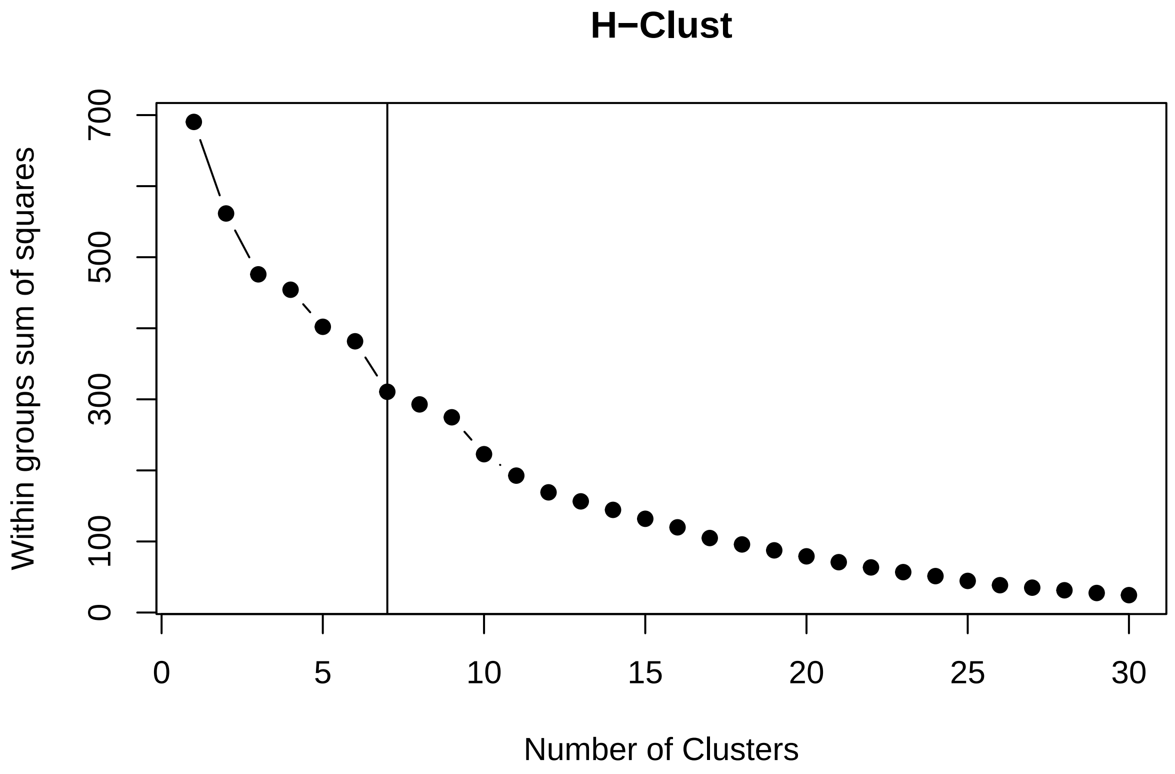
H−Clust
Within groups sum of squares
0
100
300
500
700
0
5
10
15
20
25
30
Number of Clusters

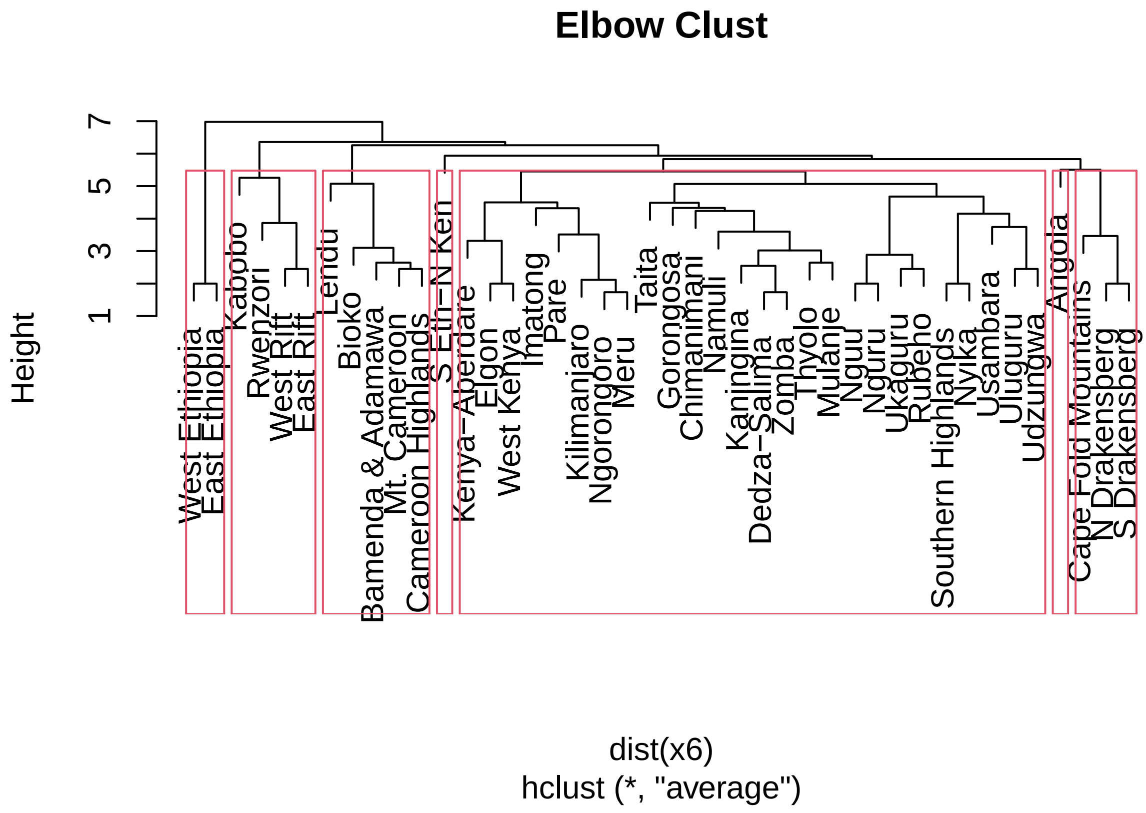
Elbow Clust
Height
West Ethiopia
East Ethiopia
Kabobo
Rwenzori
West Rift
East Rift
Lendu
Bioko
Bamenda & Adamawa
Mt. Cameroon
Cameroon Highlands
S Eth−N Ken
Kenya−Aberdare
Elgon
West Kenya
Imatong
Pare
Kilimanjaro
Ngorongoro
Meru
Taita
Gorongosa
Chimanimani
Namuli
Kaningina
Dedza−Salima
Zomba
Thyolo
Mulanje
Nguu
Nguru
Ukaguru
Rubeho
Southern Highlands
Nyika
Usambara
Uluguru
Udzungwa
Angola
Cape Fold Mountains
N Drakensberg
S Drakensberg
dist(x6)
hclust (*, "average")

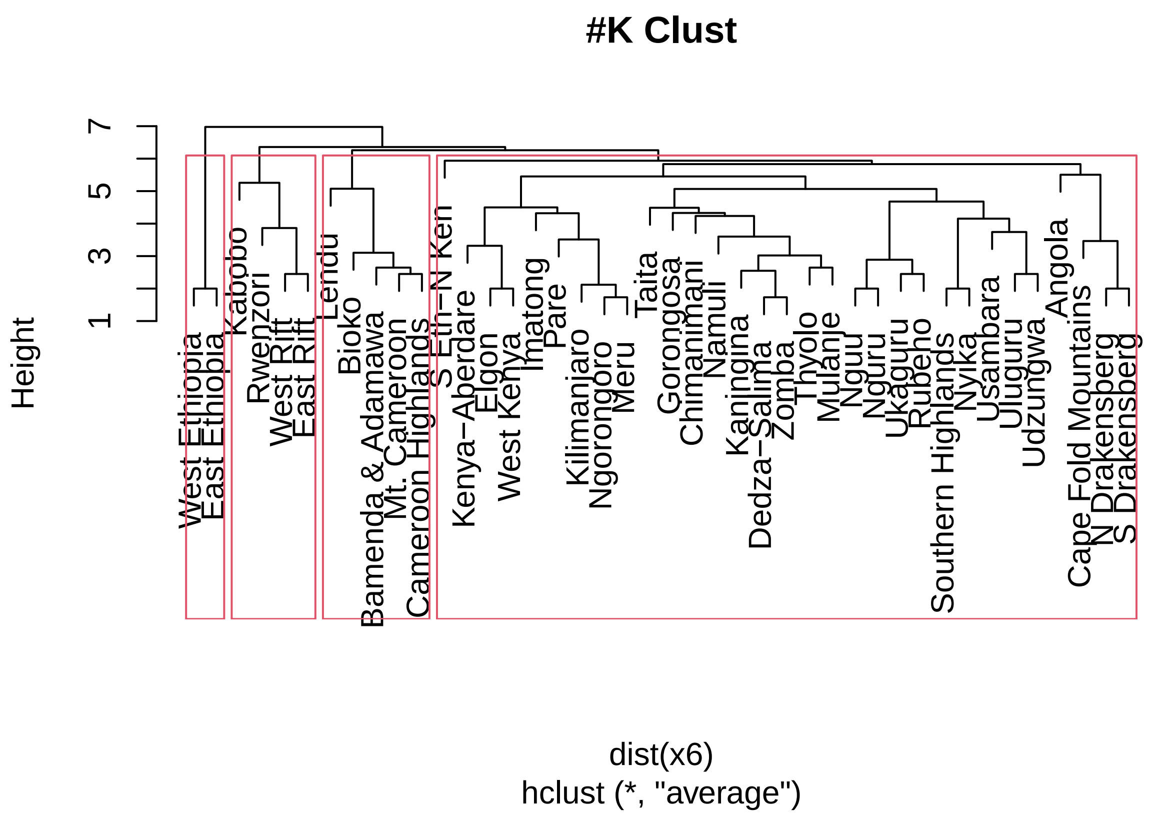
#K Clust
Height
1
3
5
7
West Ethiopia
East Ethiopia
Kabobo
Rwenzori
West Rift
East Rift
Lendu
Bioko
Bamenda & Adamawa
Mt. Cameroon
Cameroon Highlands
S Eth–N Ken
Kenya–Aberdare
Elgon
West Kenya
Imatong
Pare
Kilimanjaro
Ngorongoro
Meru
Taita
Gorongosa
Chimanimani
Namuli
Kaningina
Dedza–Salima
Zomba
Thyolo
Mulanje
Nguu
Nguru
Ukaguru
Rubeho
Southern Highlands
Nyika
Usambara
Uluguru
Udzungwa
Angola
Cape Fold Mountains
N Drakensberg
S Drakensberg
dist(x6)
hclust (*, "average")

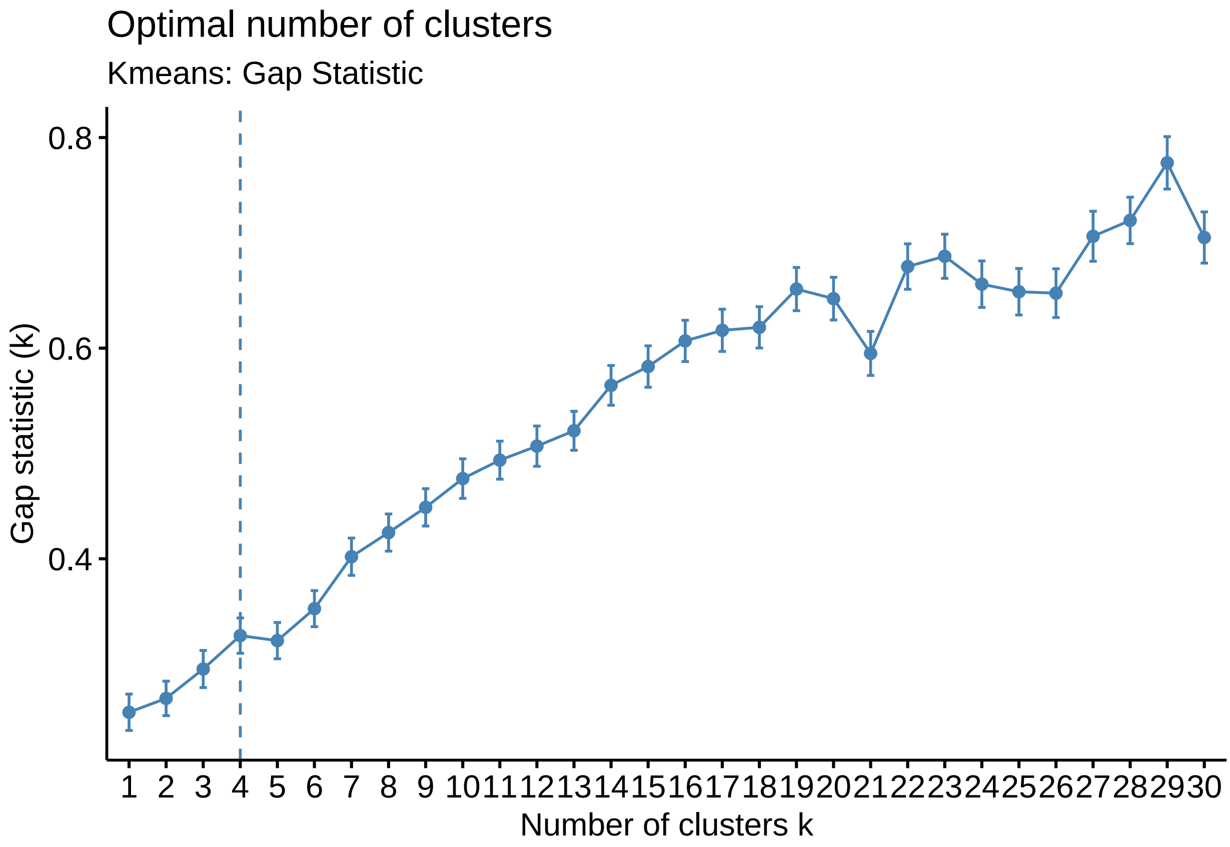


```
# Dowsett data

clustertaxa(level="Genus",xdata=dowsett.data,
            ncluster=4,hcluster=8,
            author="Dowsett")
```

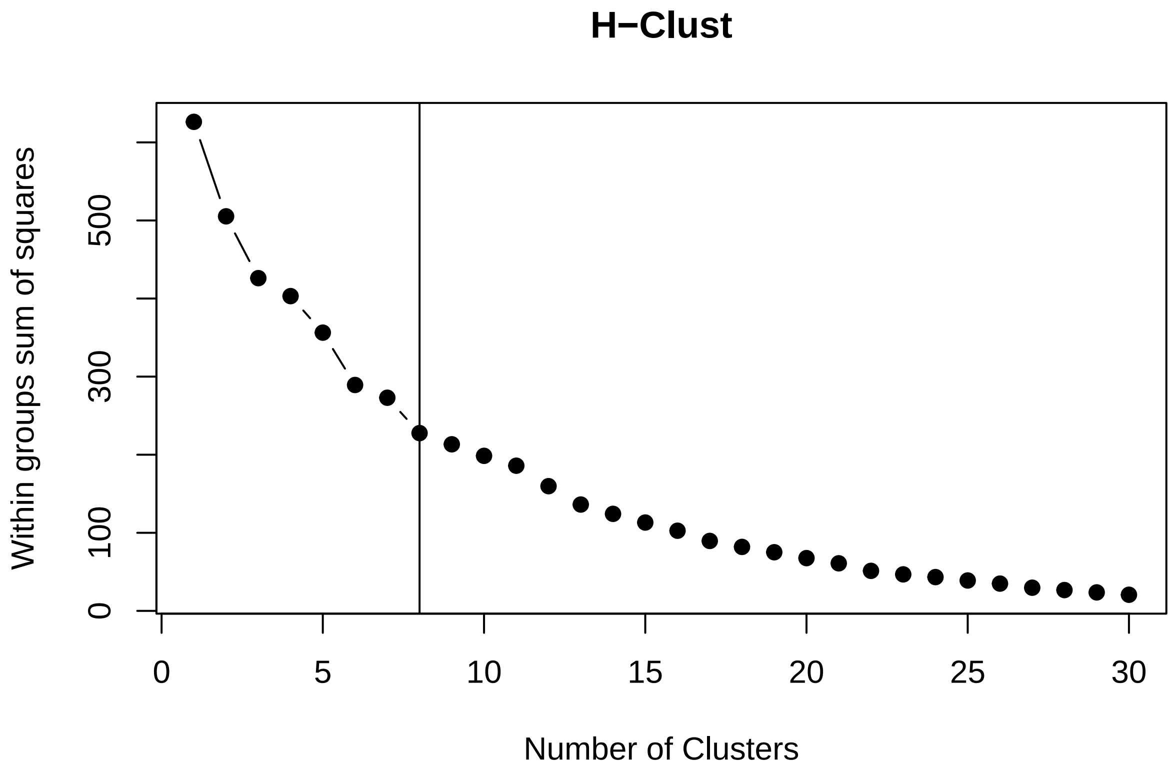
H−Clust
Within groups sum of squares
0
100
300
500
0
5
10
15
20
25
30
Number of Clusters

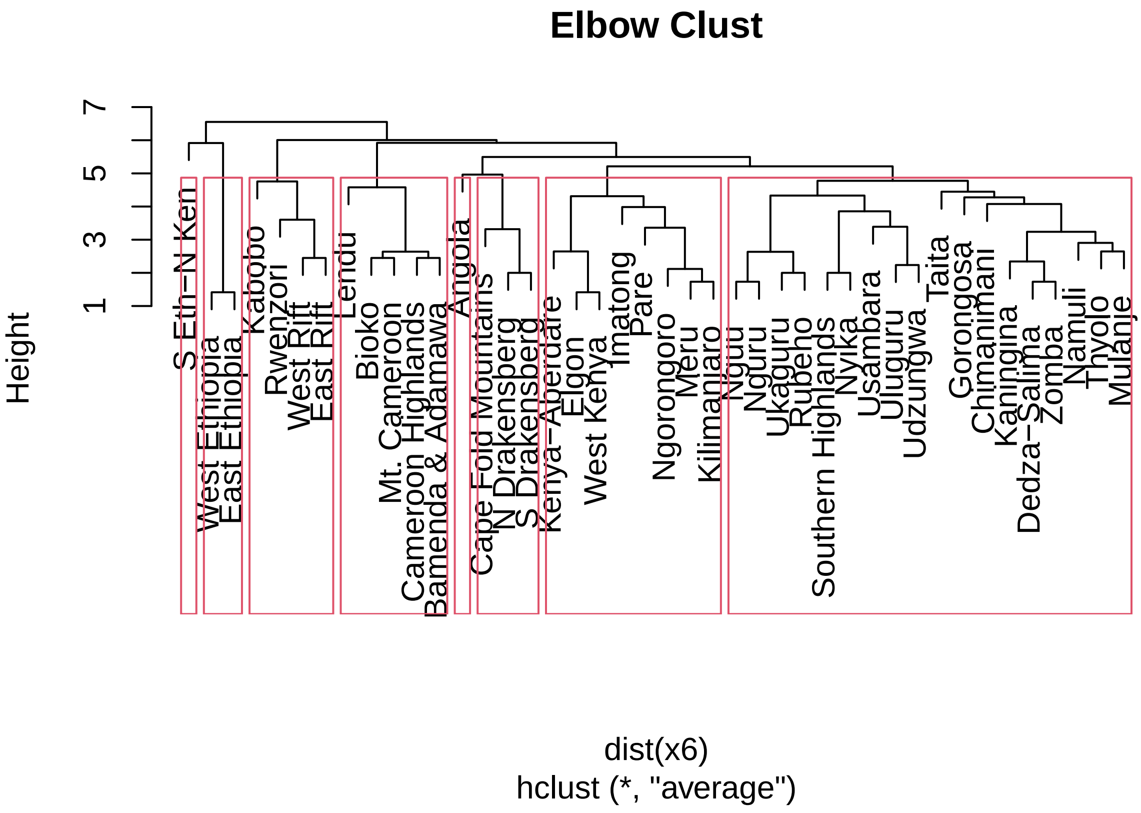
Elbow Clust
Height
1
3
5
7
S Eth−N Ken
West Ethiopia
East Ethiopia
Kabobo
Rwenzori
West Rift
East Rift
Lendu
Bioko
Mt. Cameroon
Cameroon Highlands
Bamenda & Adamawa
Angola
Cape Fold Mountains
N Drakensberg
S Drakensberg
Kenya−Aberdare
Elgon
West Kenya
Imatong
Pare
Ngorongoro
Meru
Kilimanjaro
Nguu
Nguru
Ukaguru
Rubeho
Southern Highlands
Nyika
Usambara
Uluguru
Udzungwa
Taita
Gorongosa
Chimanimani
Kaningina
Dedza−Salima
Zomba
Namuli
Thyolo
Mulanje
dist(x6)
hclust (*, "average")

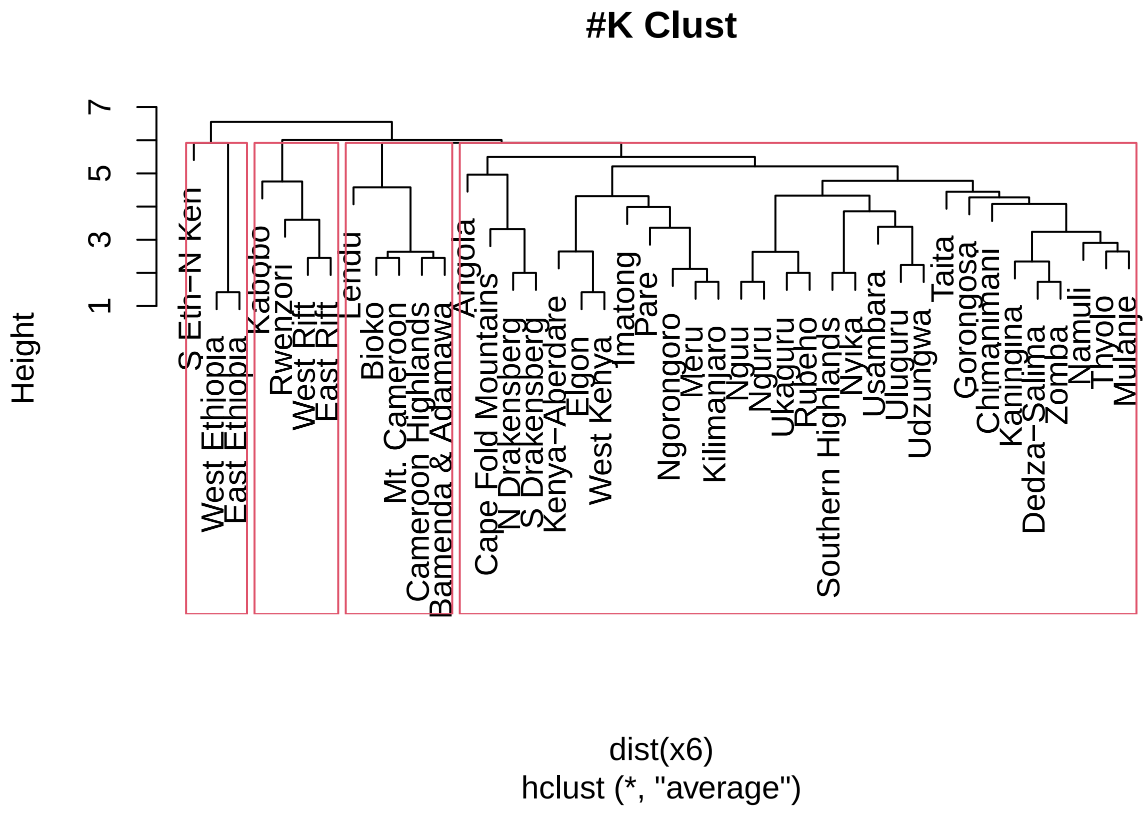
#K Clust
Height
1
3
5
7
S Eth−N Ken
West Ethiopia
East Ethiopia
Kabobo
Rwenzori
West Rift
East Rift
Lendu
Bioko
Mt. Cameroon
Cameroon Highlands
Bamenda & Adamawa
Angola
Cape Fold Mountains
N Drakensberg
S Drakensberg
Kenya−Aberdare
Elgon
West Kenya
Imatong
Pare
Ngorongoro
Meru
Kilimanjaro
Nguu
Nguru
Ukaguru
Rubeho
Southern Highlands
Nyika
Usambara
Uluguru
Udzungwa
Taita
Gorongosa
Chimanimani
Kaningina
Dedza−Salima
Zomba
Namuli
Thyolo
Mulanje
dist(x6)
hclust (*, "average")

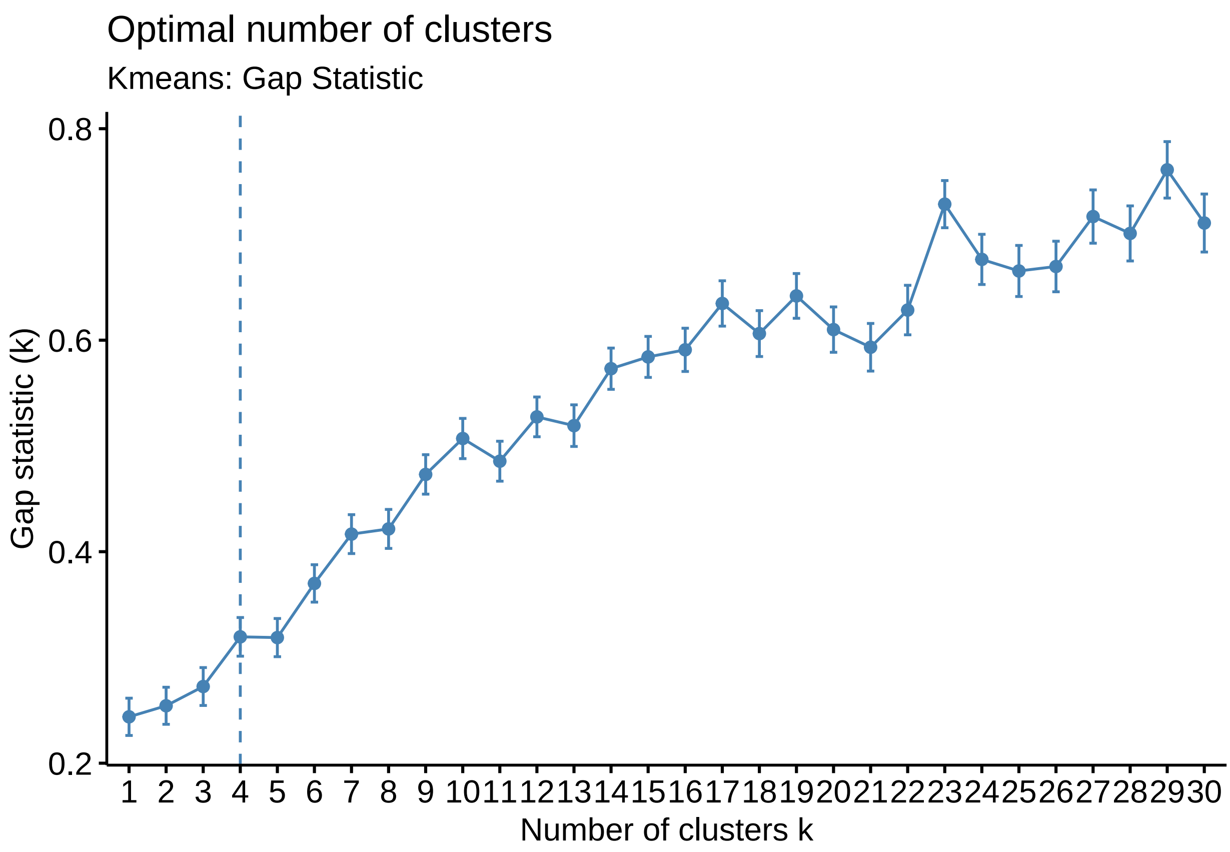

2.3 Superspecies Clusters

```
# this study

clustertaxa(level="Superspecies",xdata=xdata,
            ncluster=10,hcluster=7,
            author="Cooper")
```

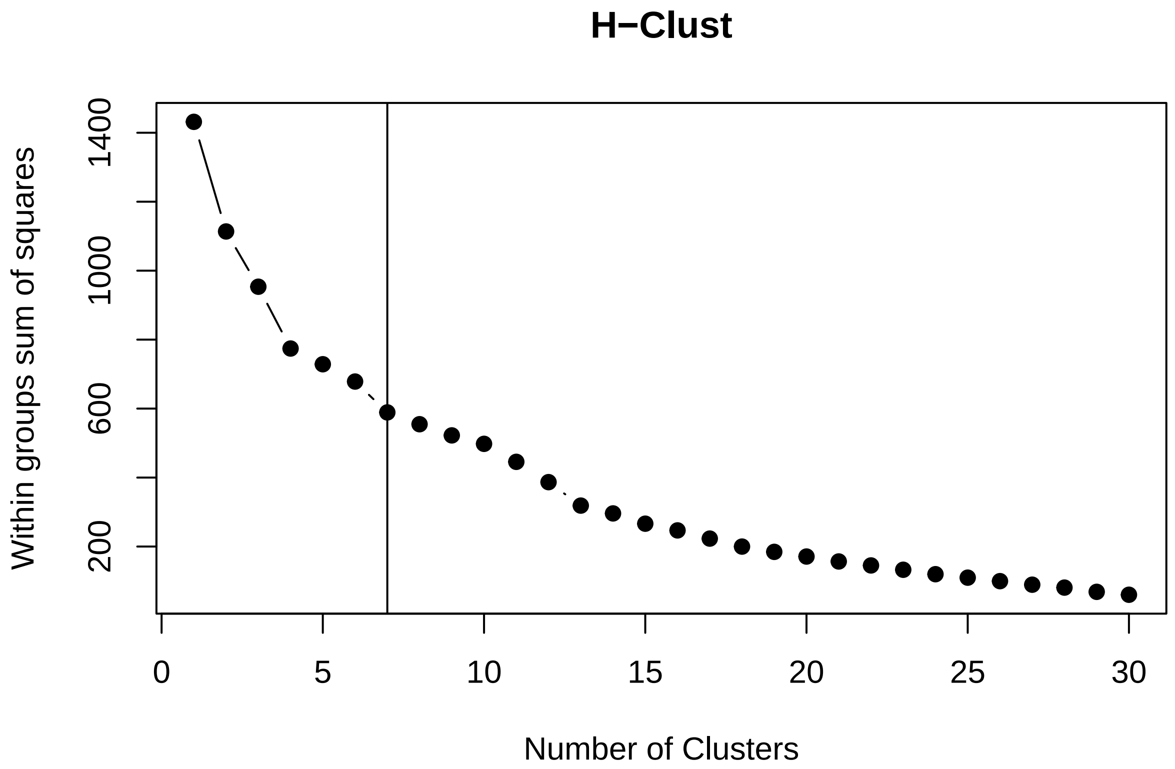
H−Clust
Within groups sum of squares
200
600
1000
1400
0
5
10
15
20
25
30
Number of Clusters

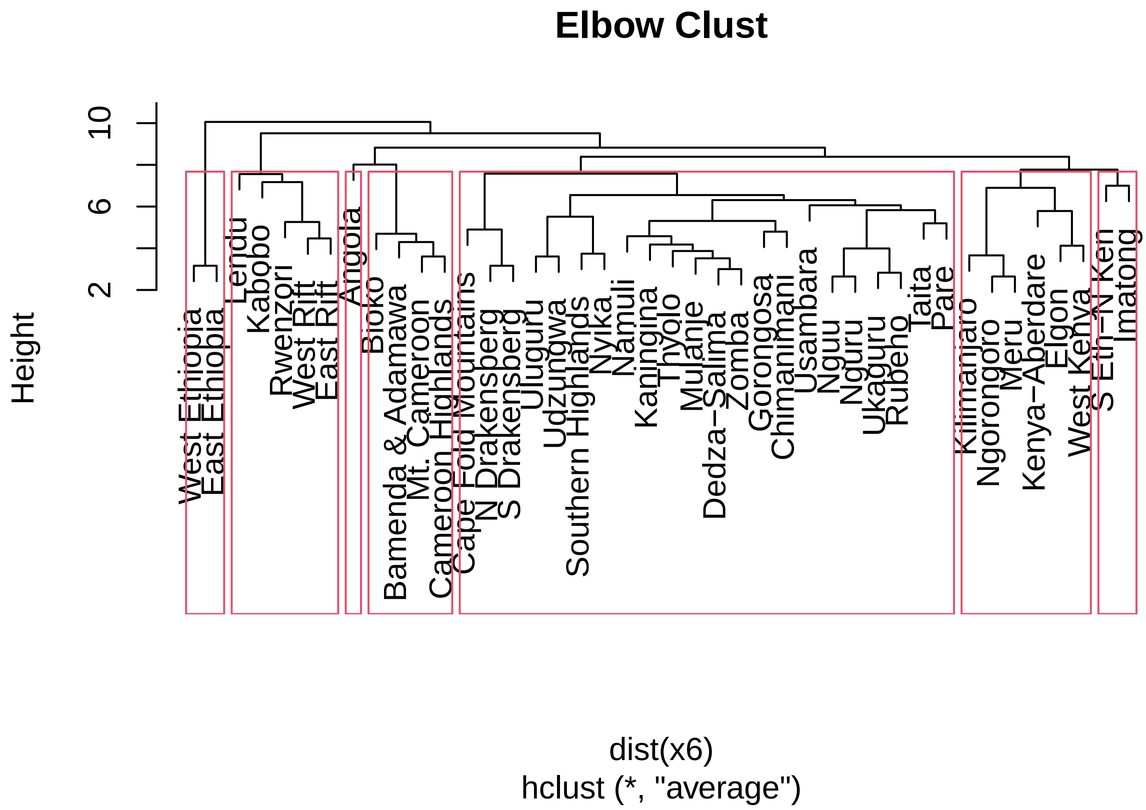

Elbow Clust
Height
10
6
2
West Ethiopia
East Ethiopia
Lendu
Kabobo
Rwenzori
West Rift
East Rift
Angola
Bioko
Bamenda & Adamawa
Mt. Cameroon
Cameroon Highlands
Cape Fold Mountains
N Drakensberg
S Drakensberg
Uluguru
Udzungwa
Southern Highlands
Nyika
Namuli
Kaningina
Thyolo
Mulanje
Dedza−Salima
Zomba
Gorongosa
Chimanimani
Usambara
Nguu
Nguru
Ukaguru
Rubeho
Taita
Pare
Kilimanjaro
Ngorongoro
Meru
Kenya−Aberdare
Elgon
West Kenya
S Eth−N Ken
Imatong
dist(x6)
hclust (*, "average")

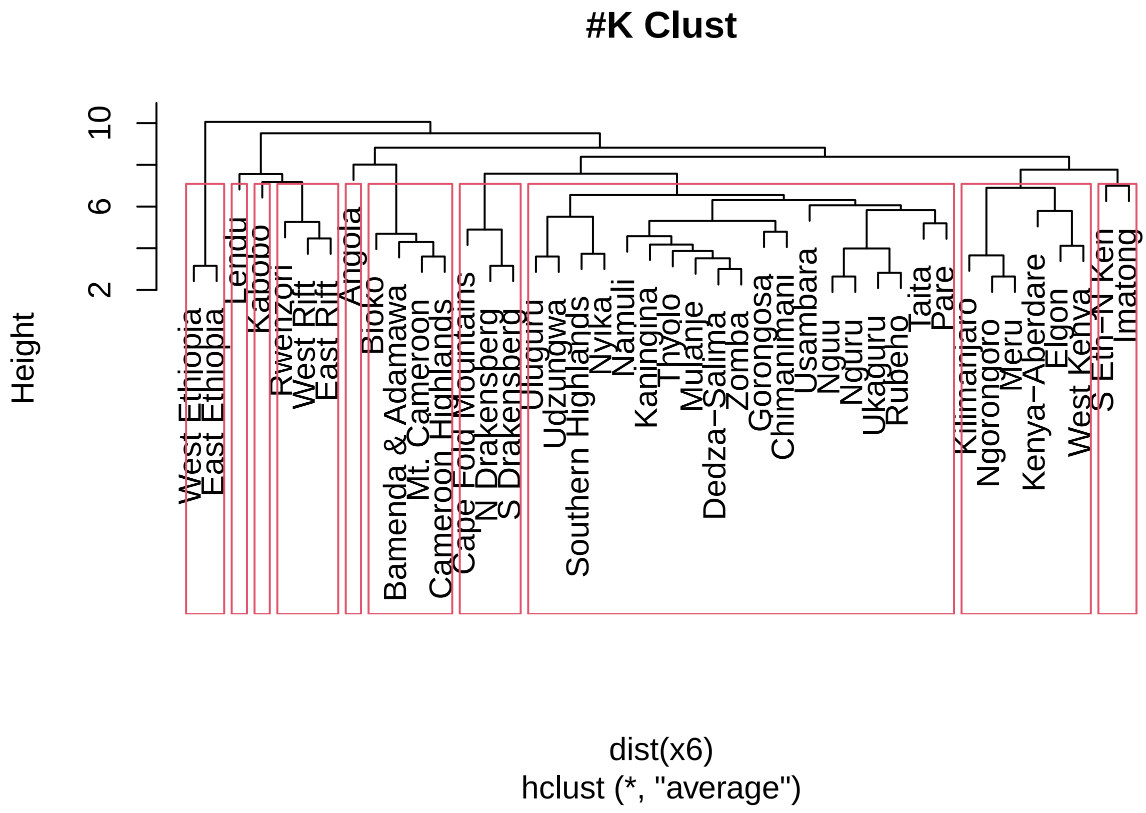
#K Clust
Height
10
6
2
West Ethiopia
East Ethiopia
Lendu
Kabobo
Rwenzori
West Rift
East Rift
Angola
Bioko
Bamenda & Adamawa
Mt. Cameroon
Cameroon Highlands
Cape Fold Mountains
N Drakensberg
S Drakensberg
Uluguru
Udzungwa
Southern Highlands
Nyika
Namuli
Kaningina
Thyolo
Mulanje
Dedza−Salima
Zomba
Gorongosa
Chimanimani
Usambara
Nguu
Nguru
Ukaguru
Rubeho
Taita
Pare
Kilimanjaro
Ngorongoro
Meru
Kenya−Aberdare
Elgon
West Kenya
S Eth−N Ken
Imatong
dist(x6)
hclust (*, "average")

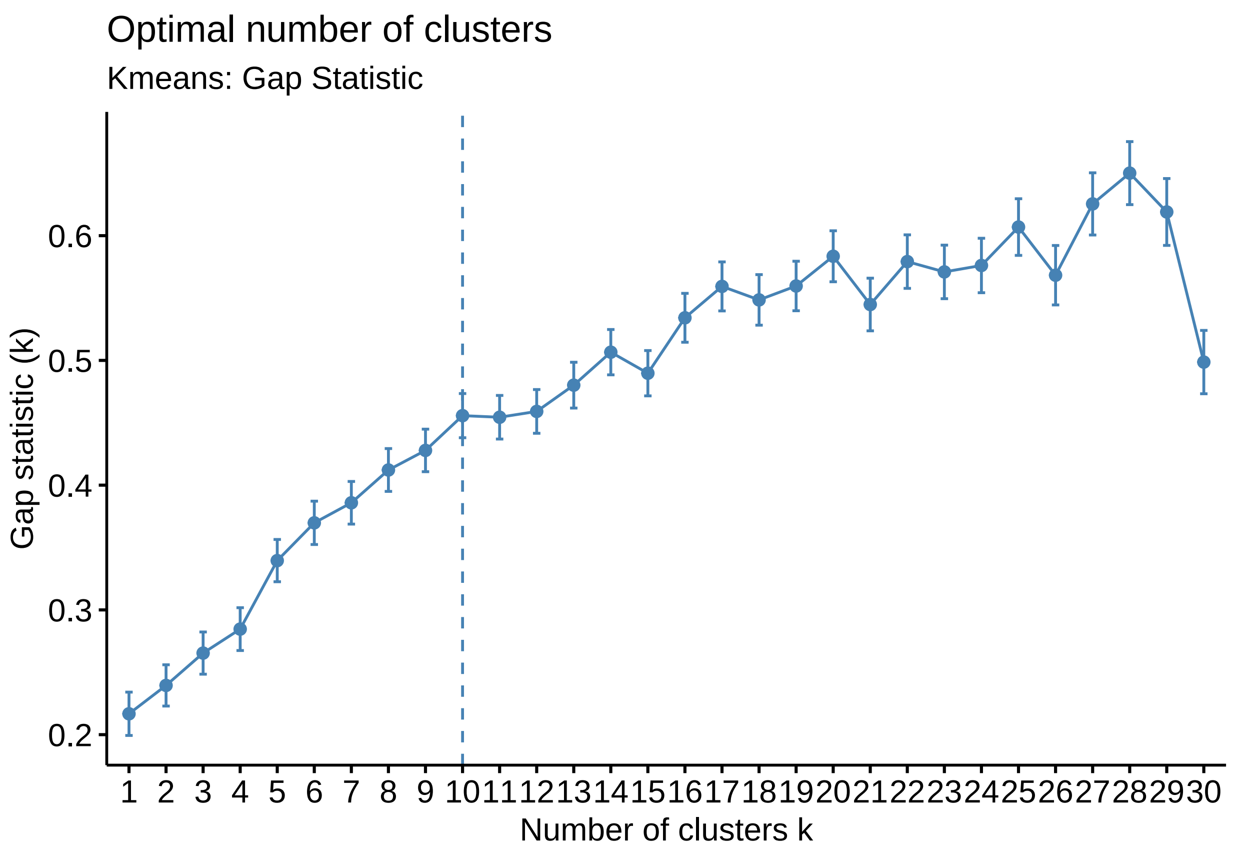


```
# Bowie data

clustertaxa(level="Superspecies",xdata=bowie.data,
            ncluster=20,hcluster=5,
            author="Bowie")
```

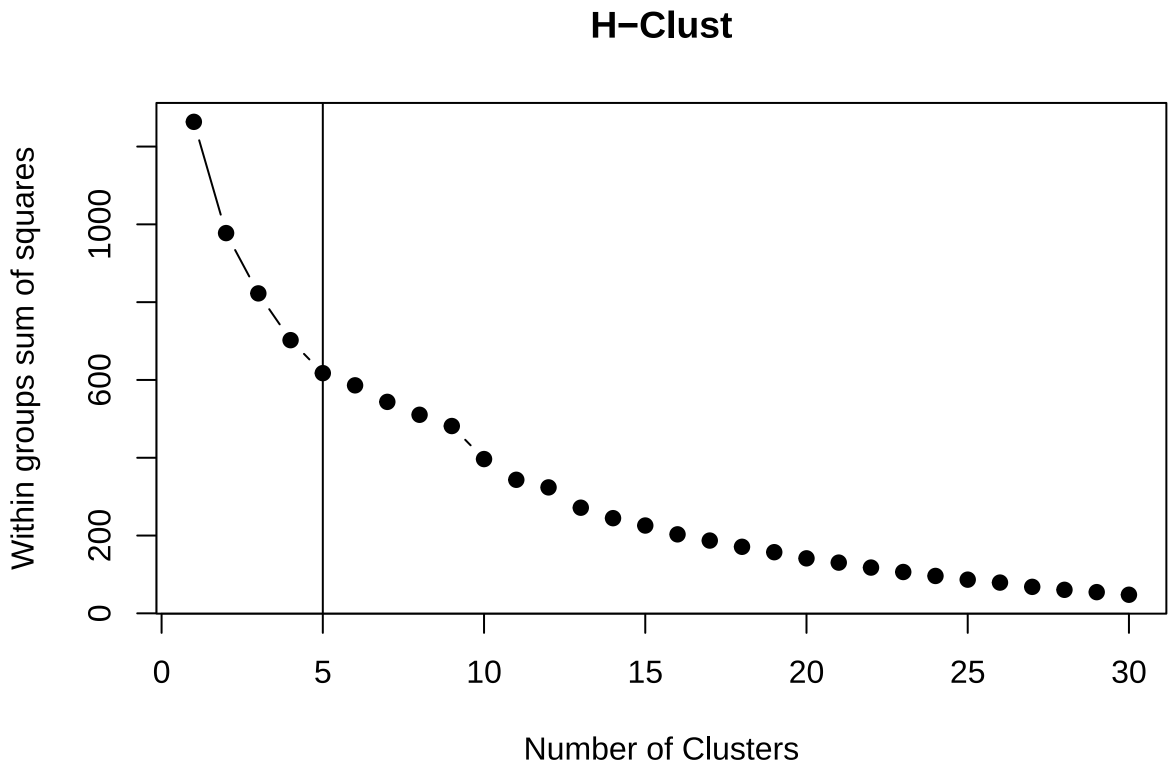
H−Clust
Within groups sum of squares
0
200
600
1000
0
5
10
15
20
25
30
Number of Clusters

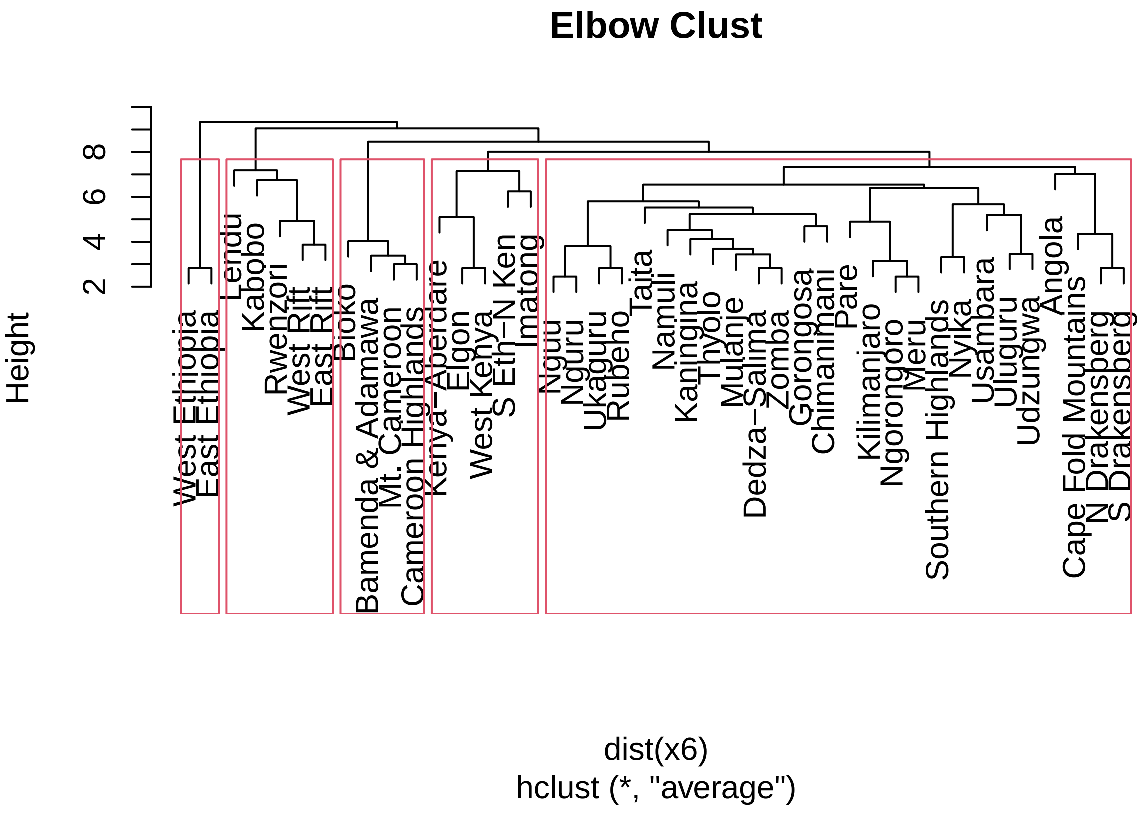
Elbow Clust
Height
2
4
6
8
West Ethiopia
East Ethiopia
Lendu
Kabobo
Rwenzori
West Rift
East Rift
Bioko
Bamenda & Adamawa
Mt. Cameroon
Cameroon Highlands
Kenya−Aberdare
Elgon
West Kenya
S Eth−N Ken
Imatong
Nguu
Nguru
Ukaguru
Rubeho
Taita
Namuli
Kaningina
Thyolo
Mulanje
Dedza−Salima
Zomba
Gorongosa
Chimanimani
Pare
Kilimanjaro
Ngorongoro
Meru
Southern Highlands
Nyika
Usambara
Uluguru
Udzungwa
Angola
Cape Fold Mountains
N Drakensberg
S Drakensberg
dist(x6)
hclust (*, "average")

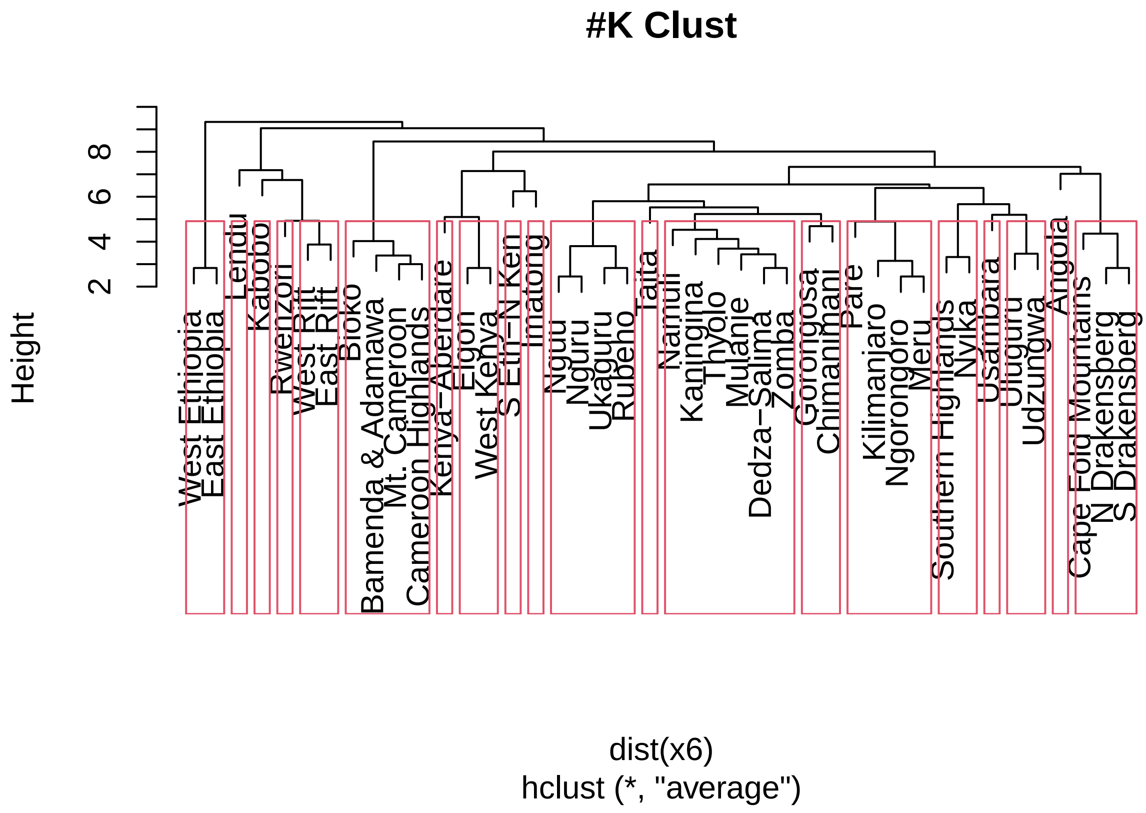

#K Clust
Height
dist(x6)
hclust (*, "average")

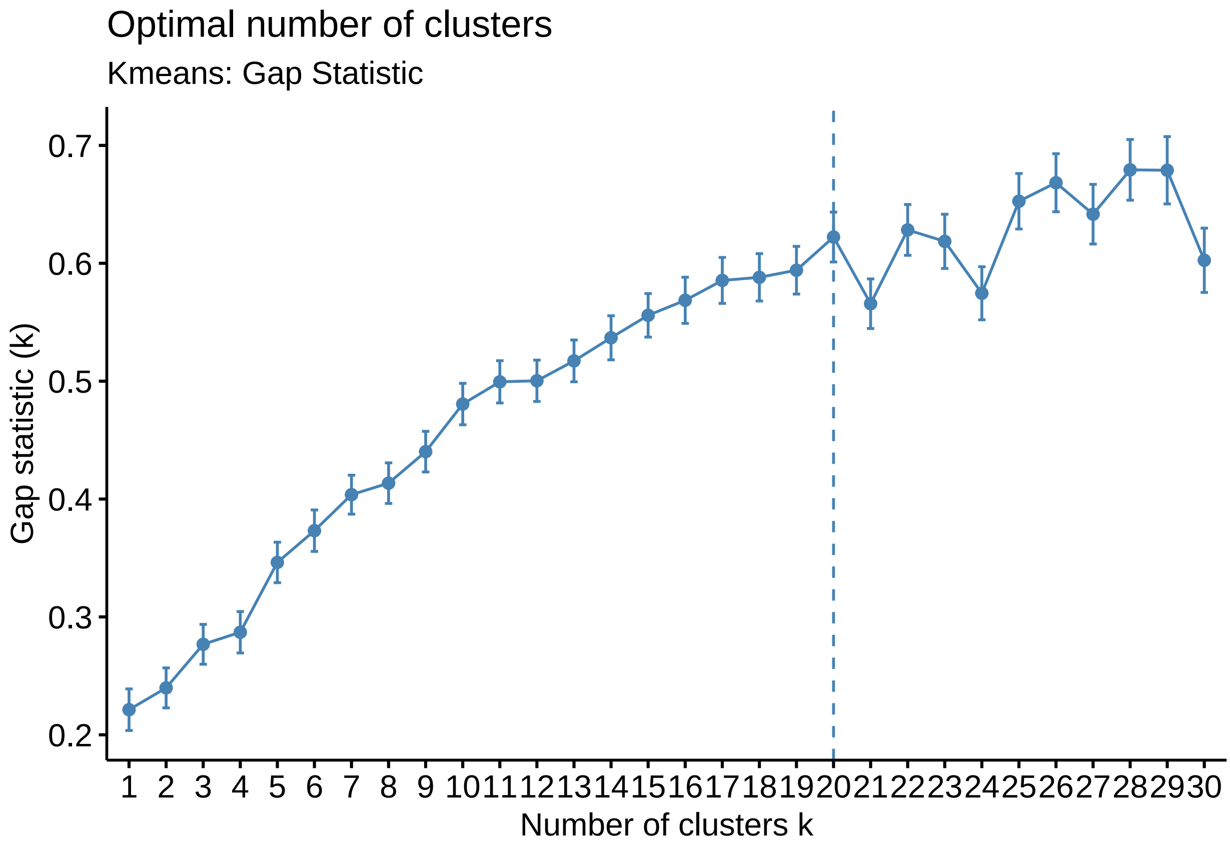


```
clustertaxa(level="Superspecies",xdata=dowsett.data,
            ncluster=13,hcluster=4,
            author="Dowsett")
```

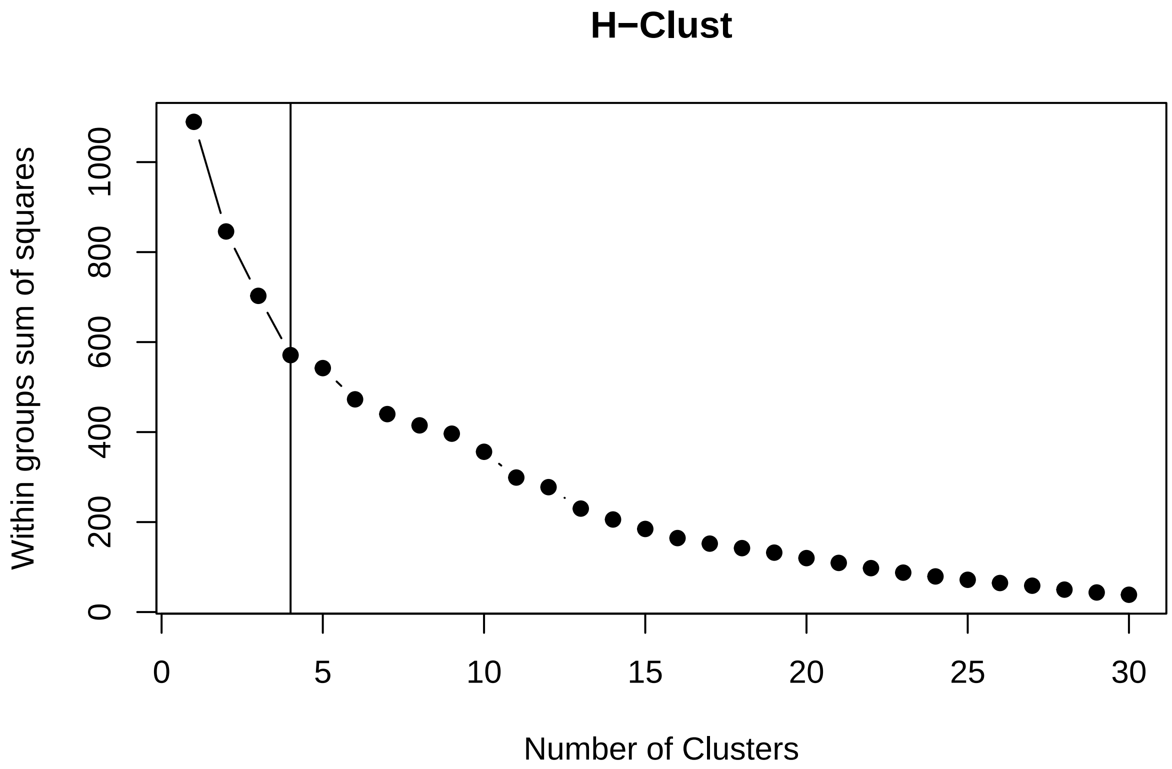
H−Clust
Within groups sum of squares
0
200
400
600
800
1000
0
5
10
15
20
25
30
Number of Clusters

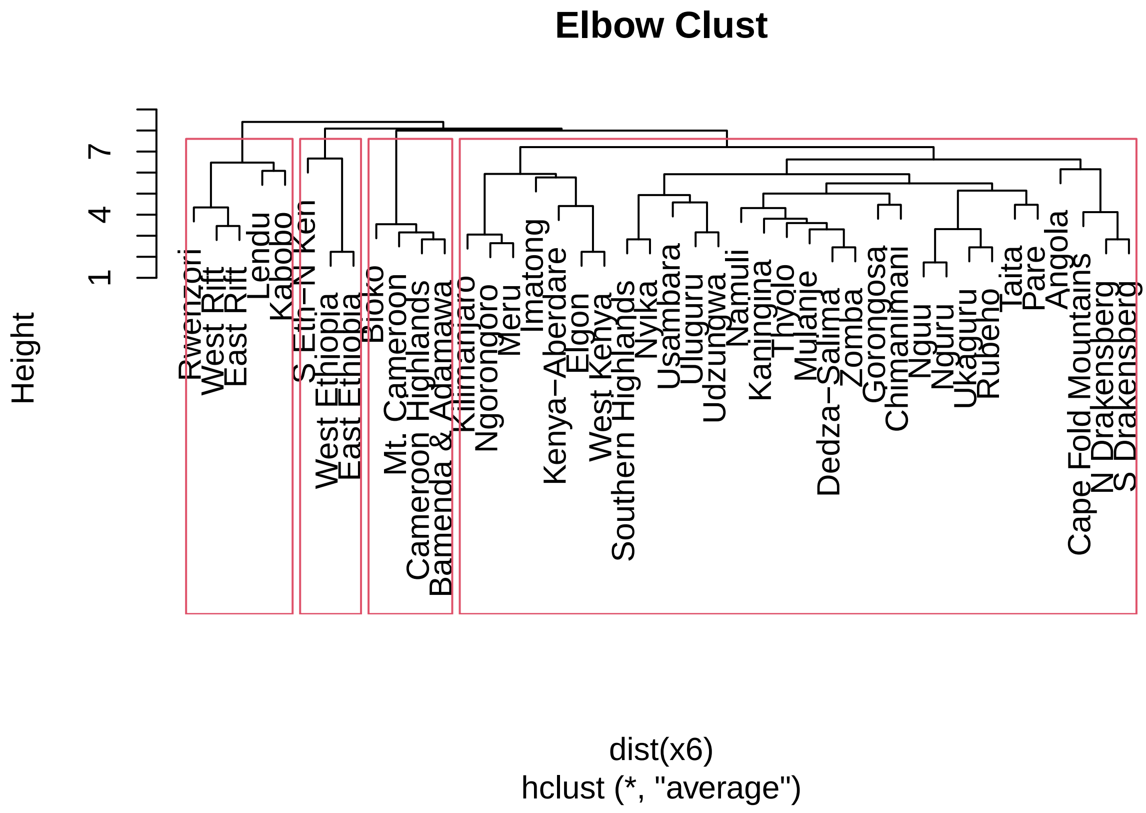
Elbow Clust
Height
1
4
7
Rwenzori
West Rift
East Rift
Lendu
Kabobo
S Eth−N Ken
West Ethiopia
East Ethiopia
Bioko
Mt. Cameroon
Cameroon Highlands
Bamenda & Adamawa
Kilimanjaro
Ngorongoro
Meru
Imatong
Kenya−Aberdare
Elgon
West Kenya
Southern Highlands
Nyika
Usambara
Uluguru
Udzungwa
Namuli
Kaningina
Thyolo
Mulanje
Dedza−Salima
Zomba
Gorongosa
Chimanimani
Nguu
Nguru
Ukaguru
Rubeho
Taita
Pare
Angola
Cape Fold Mountains
N Drakensberg
S Drakensberg
dist(x6)
hclust (*, "average")

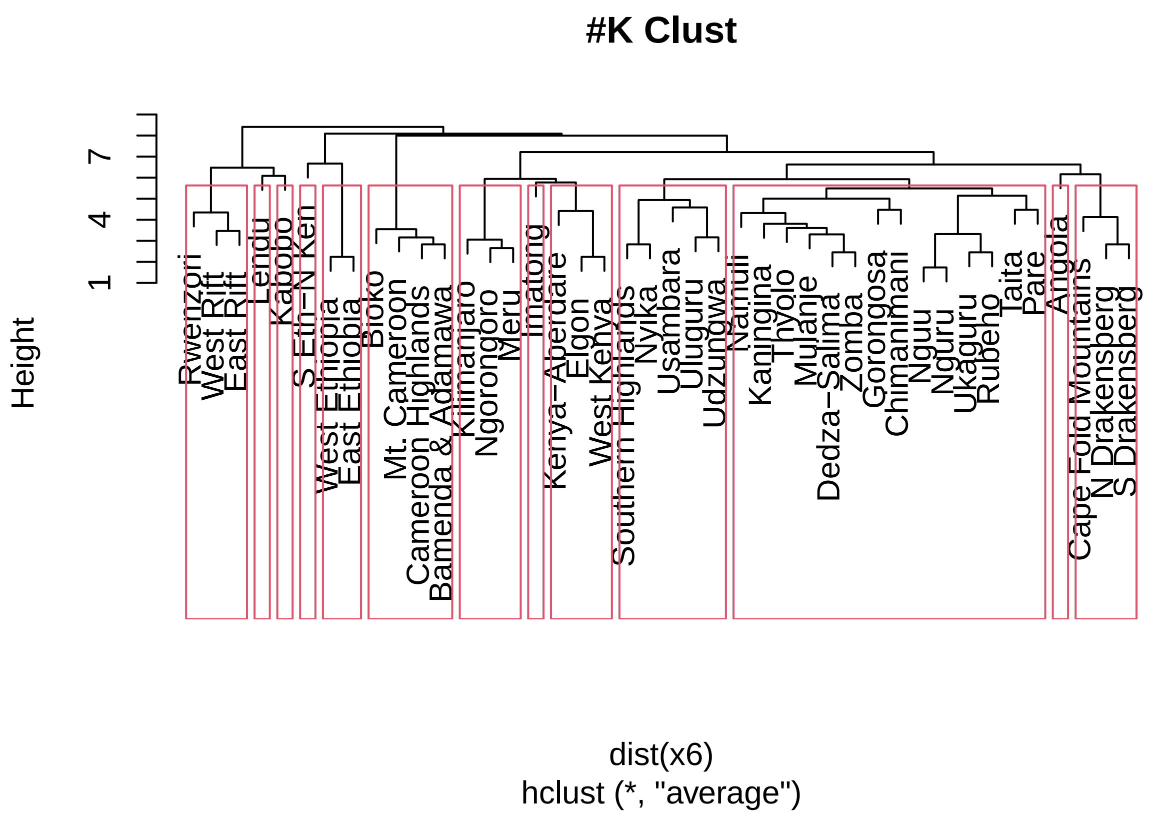
#K Clust
Height
1
4
7
Rwenzori
West Rift
East Rift
Lendu
Kabobo
S Eth−N Ken
West Ethiopia
East Ethiopia
Bioko
Mt. Cameroon
Cameroon Highlands
Bamenda & Adamawa
Kilimanjaro
Ngorongoro
Meru
Imatong
Kenya−Aberdare
Elgon
West Kenya
Southern Highlands
Nyika
Usambara
Uluguru
Udzungwa
Namuli
Kaningina
Thyolo
Mulanje
Dedza−Salima
Zomba
Gorongosa
Chimanimani
Nguu
Nguru
Ukaguru
Rubeho
Taita
Pare
Angola
Cape Fold Mountains
N Drakensberg
S Drakensberg
dist(x6)
hclust (*, "average")

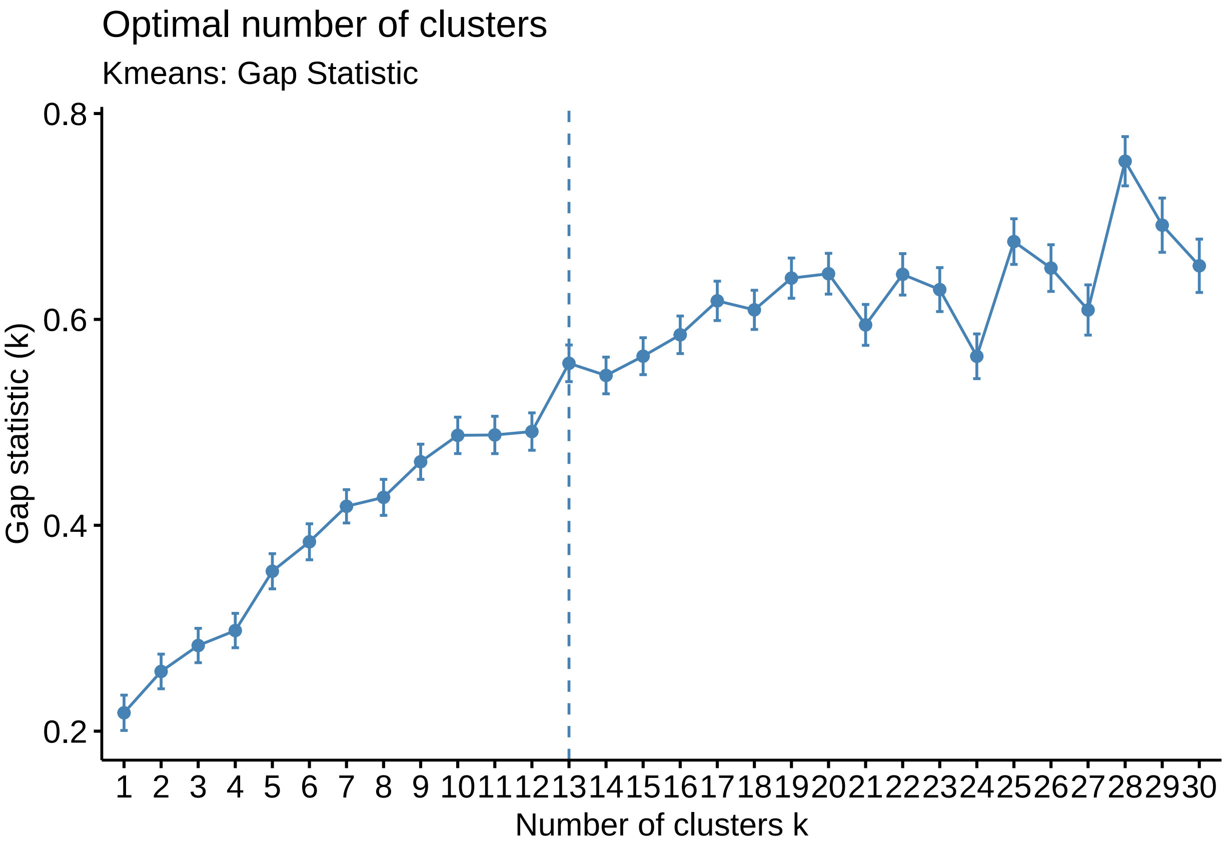

2.4 Species Cluster

```
clustertaxa(level="Species",xdata=xdata,
            ncluster=10,hcluster=4,
            author="Cooper")
```

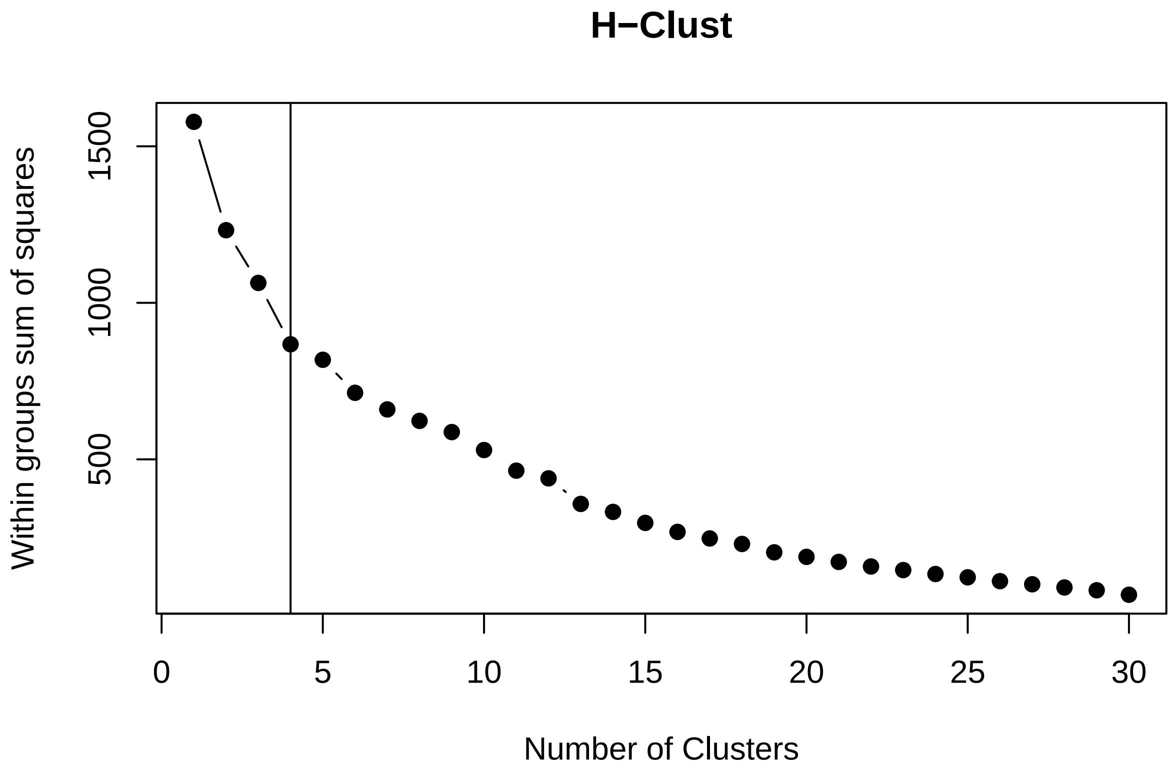
H−Clust
Within groups sum of squares
500
1000
1500
0
5
10
15
20
25
30
Number of Clusters

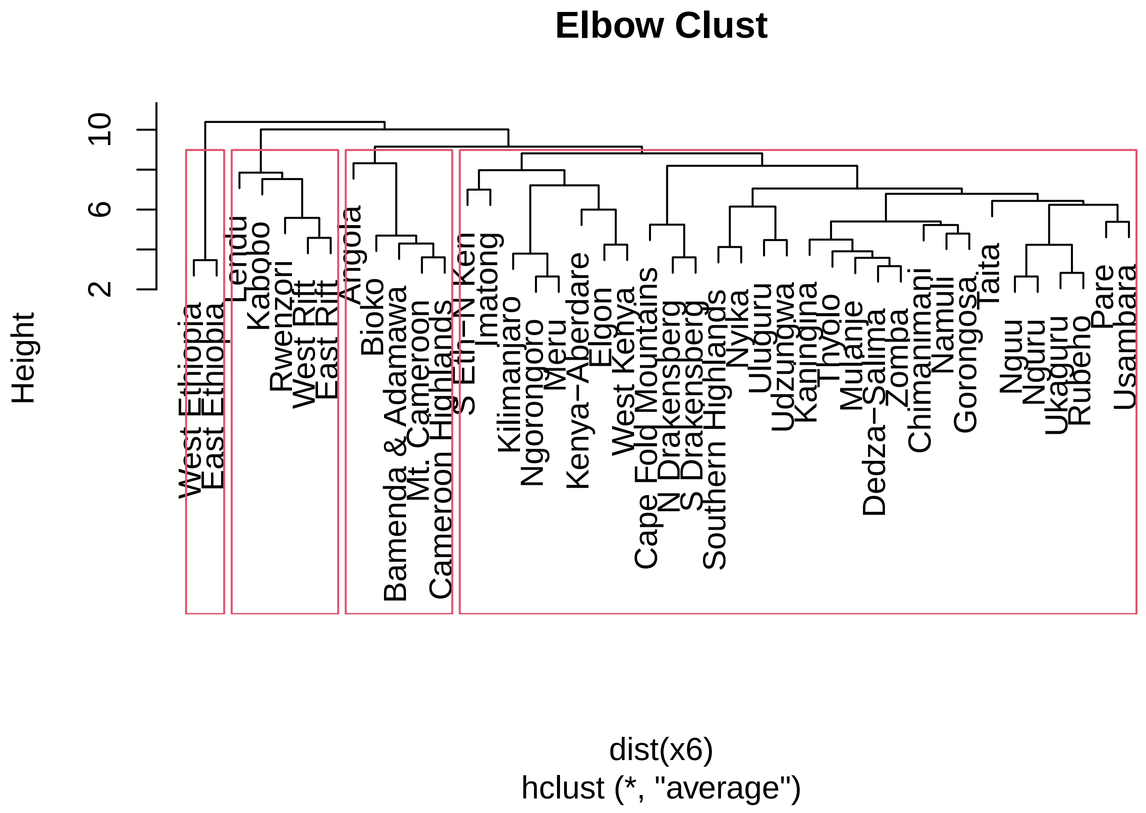
Elbow Clust
Height
10
6
2
West Ethiopia
East Ethiopia
Lendu
Kabobo
Rwenzori
West Rift
East Rift
Angola
Bioko
Bamenda & Adamawa
Mt. Cameroon
Cameroon Highlands
S Eth–N Ken
Imatong
Kilimanjaro
Ngorongoro
Meru
Kenya–Aberdare
Elgon
West Kenya
Cape Fold Mountains
N Drakensberg
S Drakensberg
Southern Highlands
Nyika
Uluguru
Udzungwa
Kaningina
Thyolo
Mulanje
Dedza–Salima
Zomba
Chimanimani
Namuli
Gorongosa
Taita
Nguu
Nguru
Ukaguru
Rubeho
Pare
Usambara
dist(x6)
hclust (*, "average")

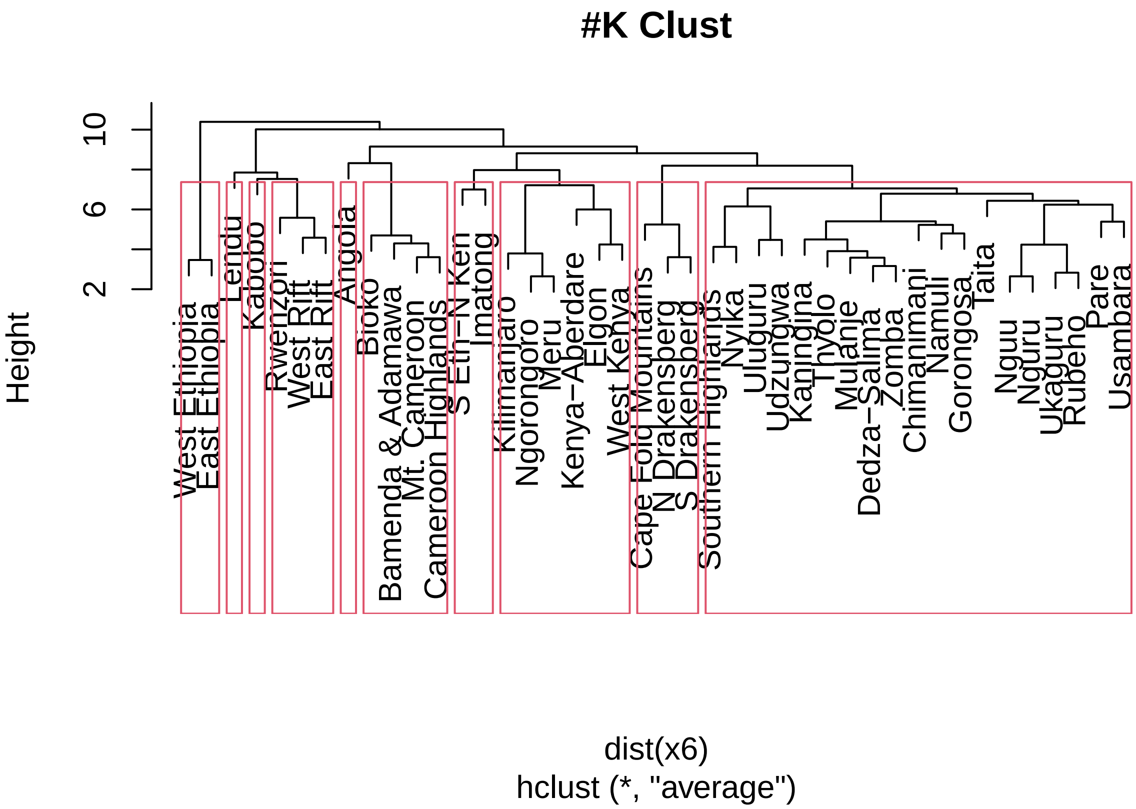
#K Clust
Height
dist(x6)
hclust (*, "average")
West Ethiopia
East Ethiopia
Lendu
Kabobo
Rwenzori
West Rift
East Rift
Angola
Bioko
Bamenda & Adamawa
Mt. Cameroon
Cameroon Highlands
S Eth–N Ken
Imatong
Kilimanjaro
Ngorongoro
Meru
Kenya–Aberdare
Elgon
West Kenya
Cape Fold Mountains
N Drakensberg
S Drakensberg
Southern Highlands
Nyika
Uluguru
Udzungwa
Kaningina
Thyolo
Mulanje
Dedza–Salima
Zomba
Chimanimani
Namuli
Gorongosa
Taita
Nguu
Nguru
Ukaguru
Rubeho
Pare
Usambara

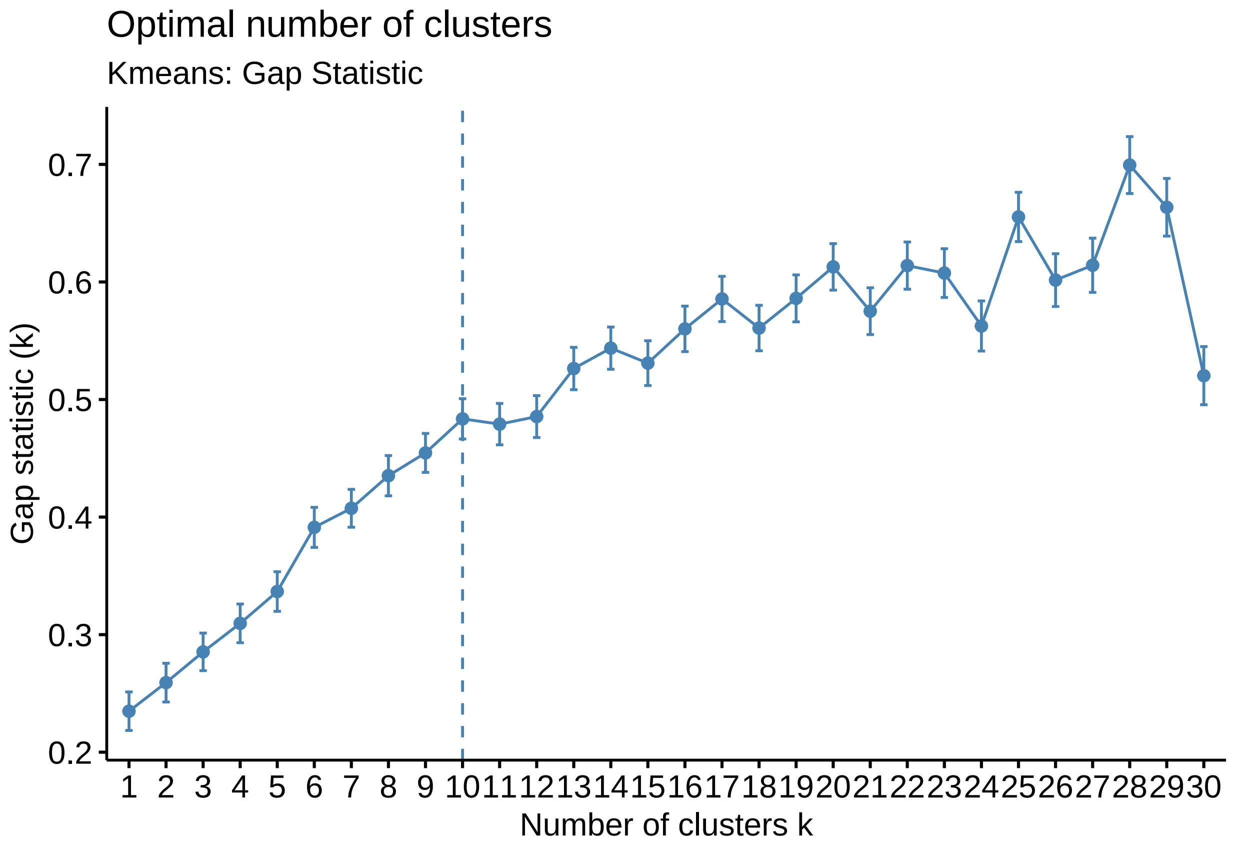


```
clustertaxa(level="Species",xdata=bowie.data,
            ncluster=10,hcluster=4,
            author="Bowie")
```

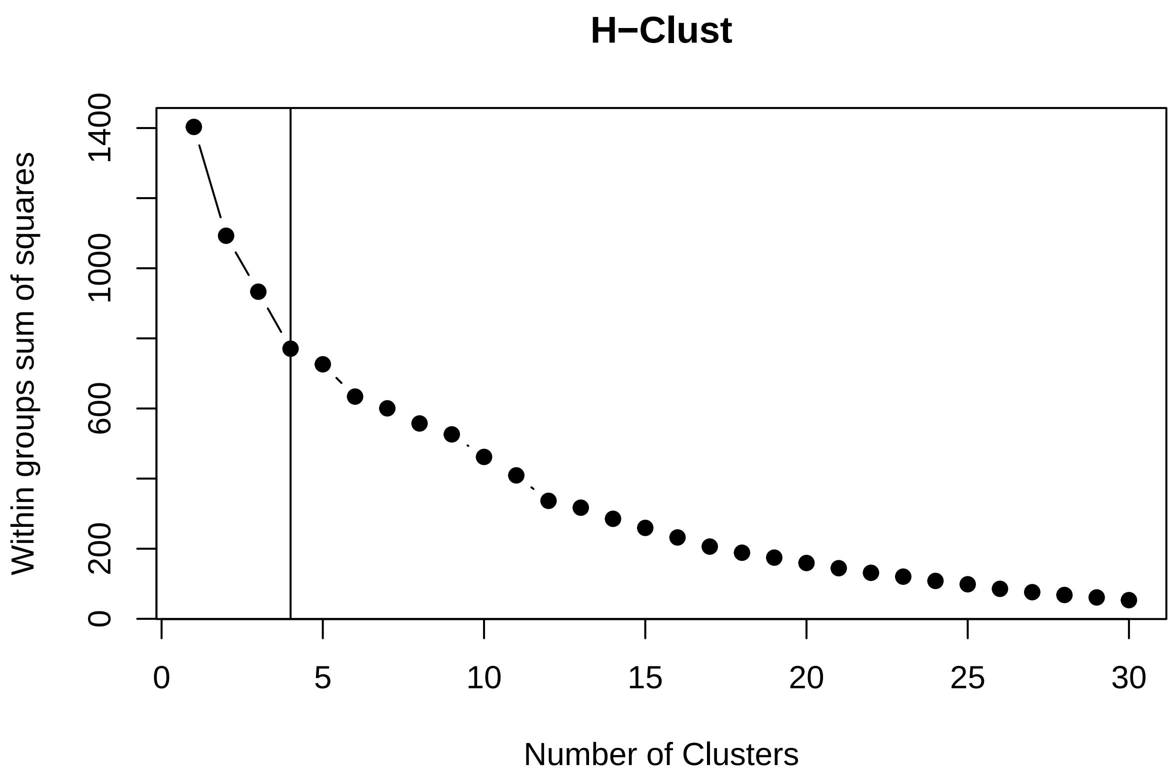

H−Clust
Within groups sum of squares
0
200
600
1000
1400
0
5
10
15
20
25
30
Number of Clusters

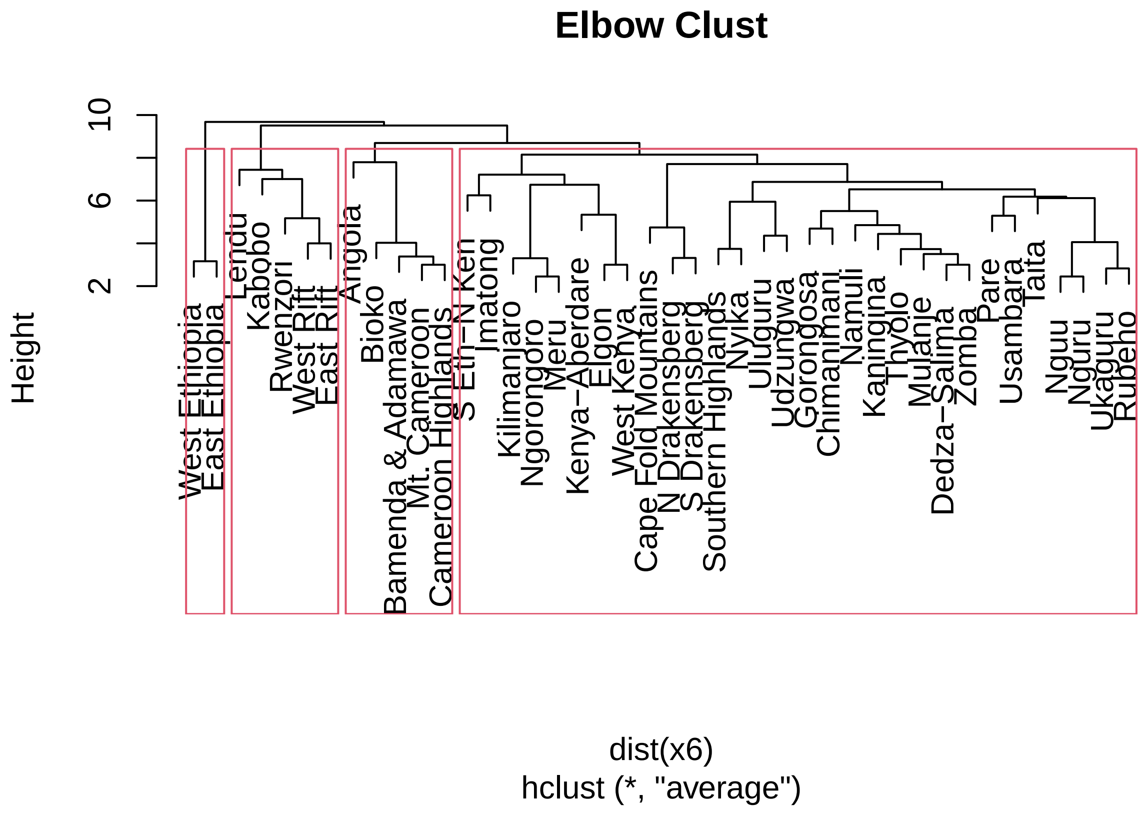
Elbow Clust
Height
10
6
2
West Ethiopia
East Ethiopia
Lendu
Kabobo
Rwenzori
West Rift
East Rift
Angola
Bioko
Bamenda & Adamawa
Mt. Cameroon
Cameroon Highlands
S Eth−N Ken
Imatong
Kilimanjaro
Ngorongoro
Meru
Kenya−Aberdare
Elgon
West Kenya
Cape Fold Mountains
N Drakensberg
S Drakensberg
Southern Highlands
Nyika
Uluguru
Udzungwa
Gorongosa
Chimanimani
Namuli
Kaningina
Thyolo
Mulanje
Dedza−Salima
Zomba
Pare
Usambara
Taita
Nguu
Nguru
Ukaguru
Rubeho
dist(x6)
hclust (*, "average")

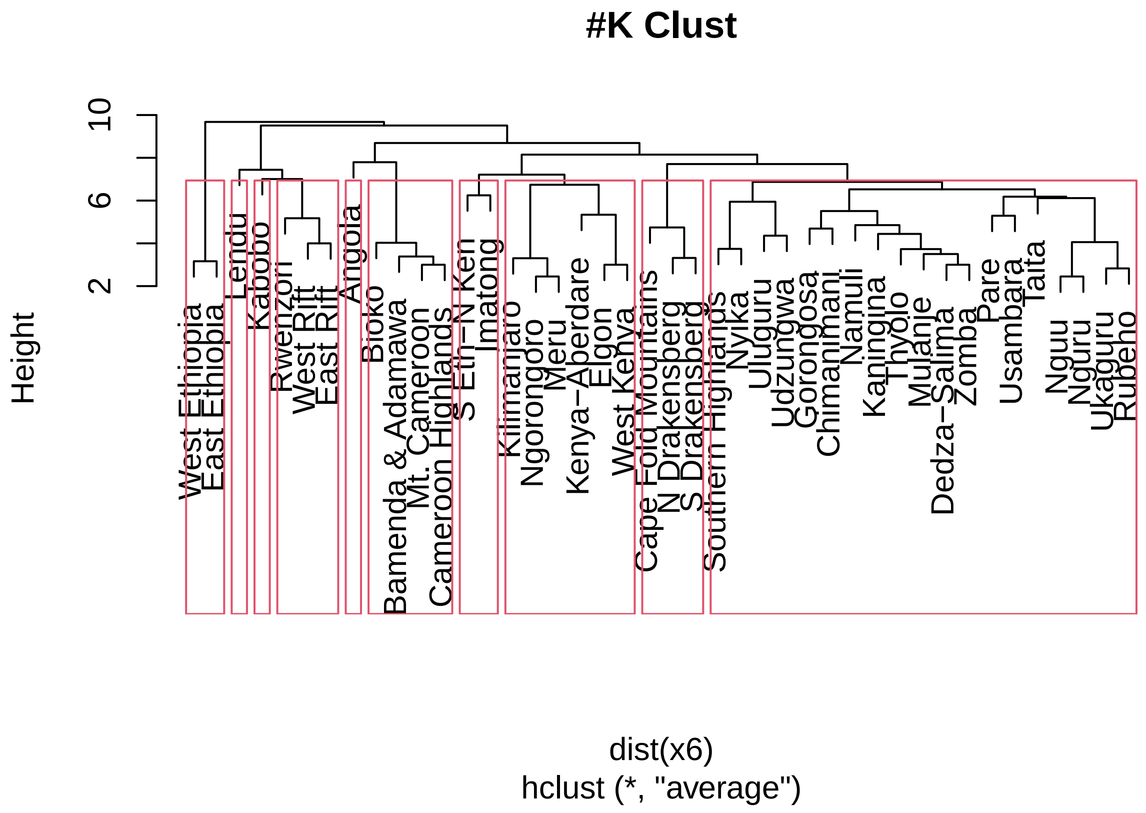

#K Clust
Height
10
6
2
West Ethiopia
East Ethiopia
Lendu
Kabobo
Rwenzori
West Rift
East Rift
Angola
Bioko
Bamenda & Adamawa
Mt. Cameroon
Cameroon Highlands
S Eth–N Ken
Imatong
Kilimanjaro
Ngorongoro
Meru
Kenya–Aberdare
Elgon
West Kenya
Cape Fold Mountains
N Drakensberg
S Drakensberg
Southern Highlands
Nyika
Uluguru
Udzungwa
Gorongosa
Chimanimani
Namuli
Kaningina
Thyolo
Mulanje
Dedza–Salima
Zomba
Pare
Usambara
Taita
Nguu
Nguru
Ukaguru
Rubeho
dist(x6)
hclust (*, "average")

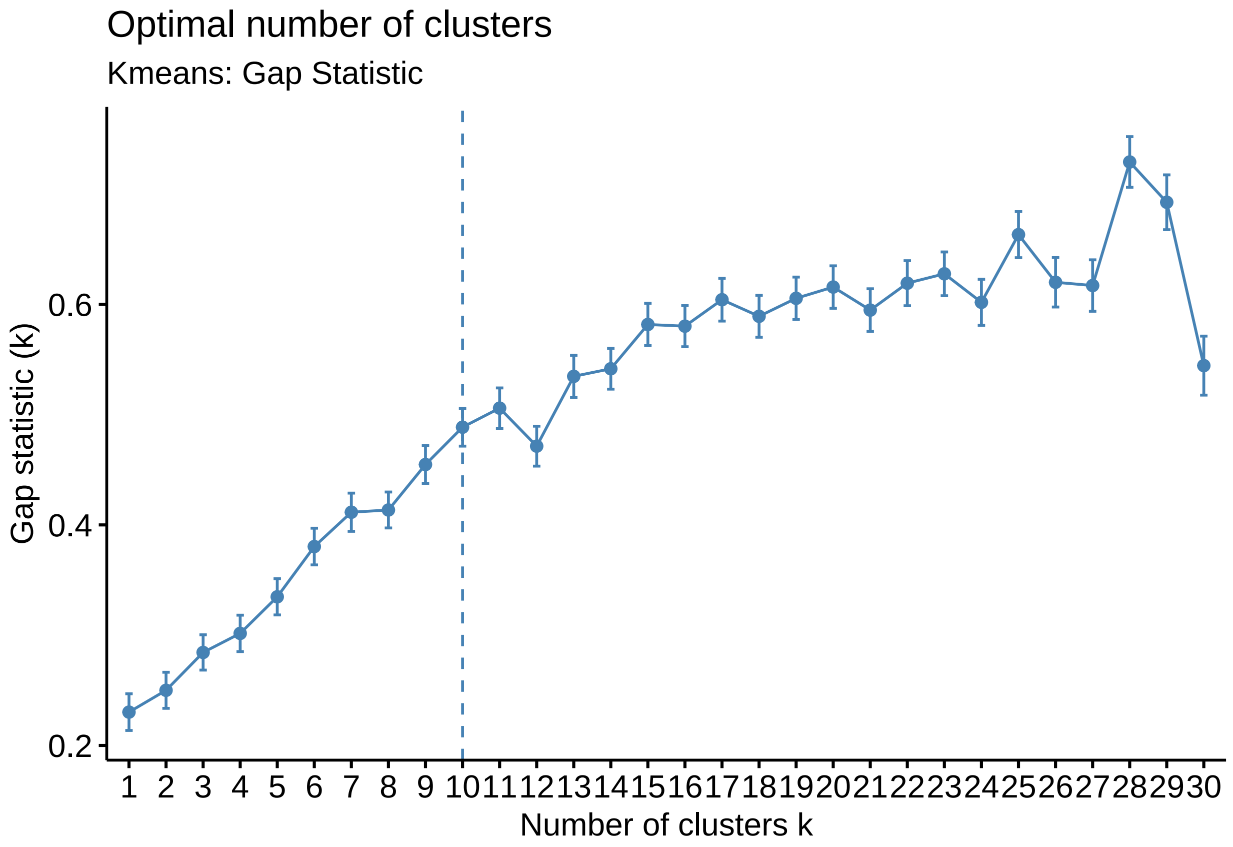


```
clustertaxa(level="Species",xdata=dowsett.data,
            ncluster=5,hcluster=5,
            author="Dowsett")
```

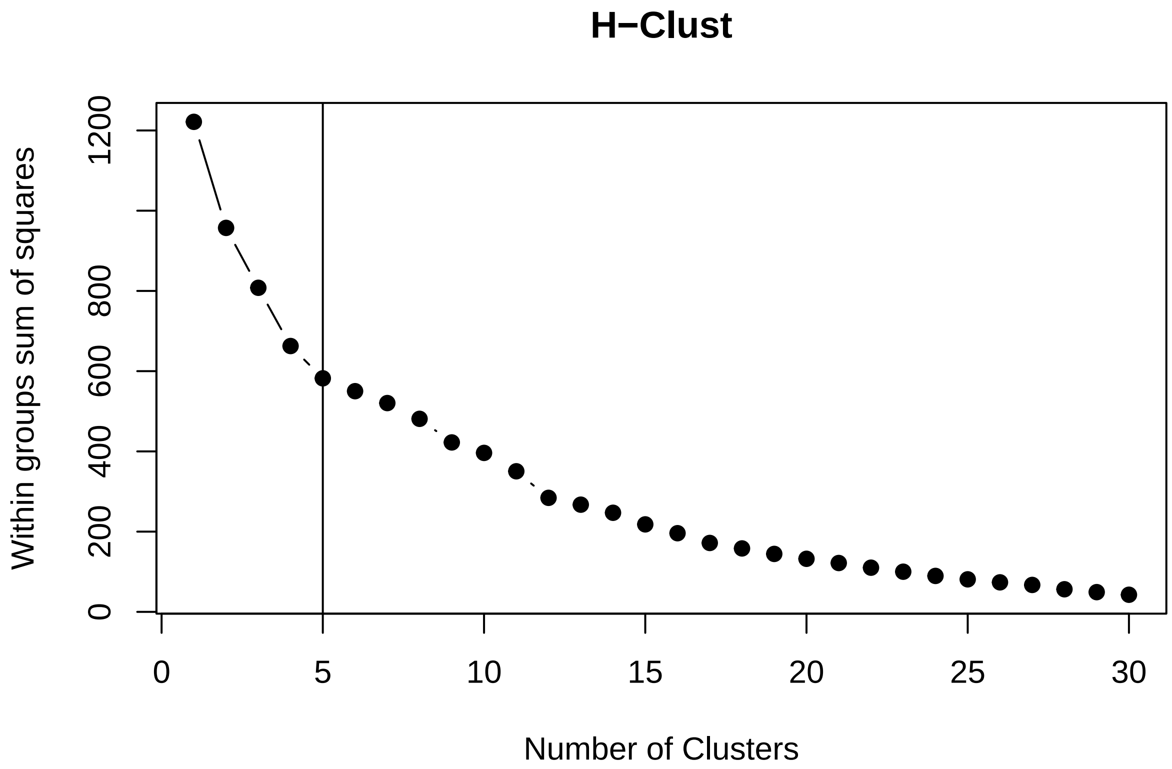
H−Clust
Within groups sum of squares
0
200
400
600
800
1200
0
5
10
15
20
25
30
Number of Clusters

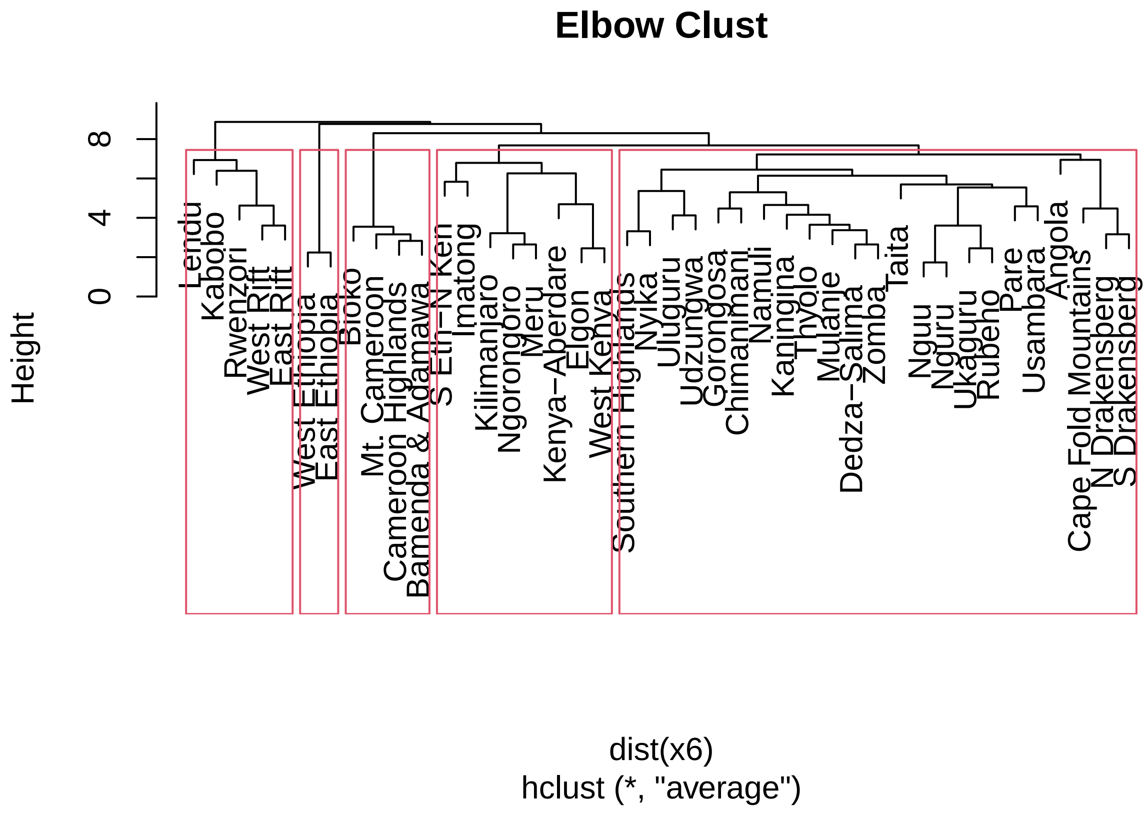
Elbow Clust
Height
0
4
8
Lendu
Kabobo
Rwenzori
West Rift
East Rift
West Ethiopia
East Ethiopia
Bioko
Mt. Cameroon
Cameroon Highlands
Bamenda & Adamawa
S Eth−N Ken
Imatong
Kilimanjaro
Ngorongoro
Meru
Kenya−Aberdare
Elgon
West Kenya
Southern Highlands
Nyika
Uluguru
Udzungwa
Gorongosa
Chimanimani
Namuli
Kaningina
Thyolo
Mulanje
Dedza−Salima
Zomba
Taita
Nguu
Nguru
Ukaguru
Rubeho
Pare
Usambara
Angola
Cape Fold Mountains
N Drakensberg
S Drakensberg
dist(x6)
hclust (*, "average")

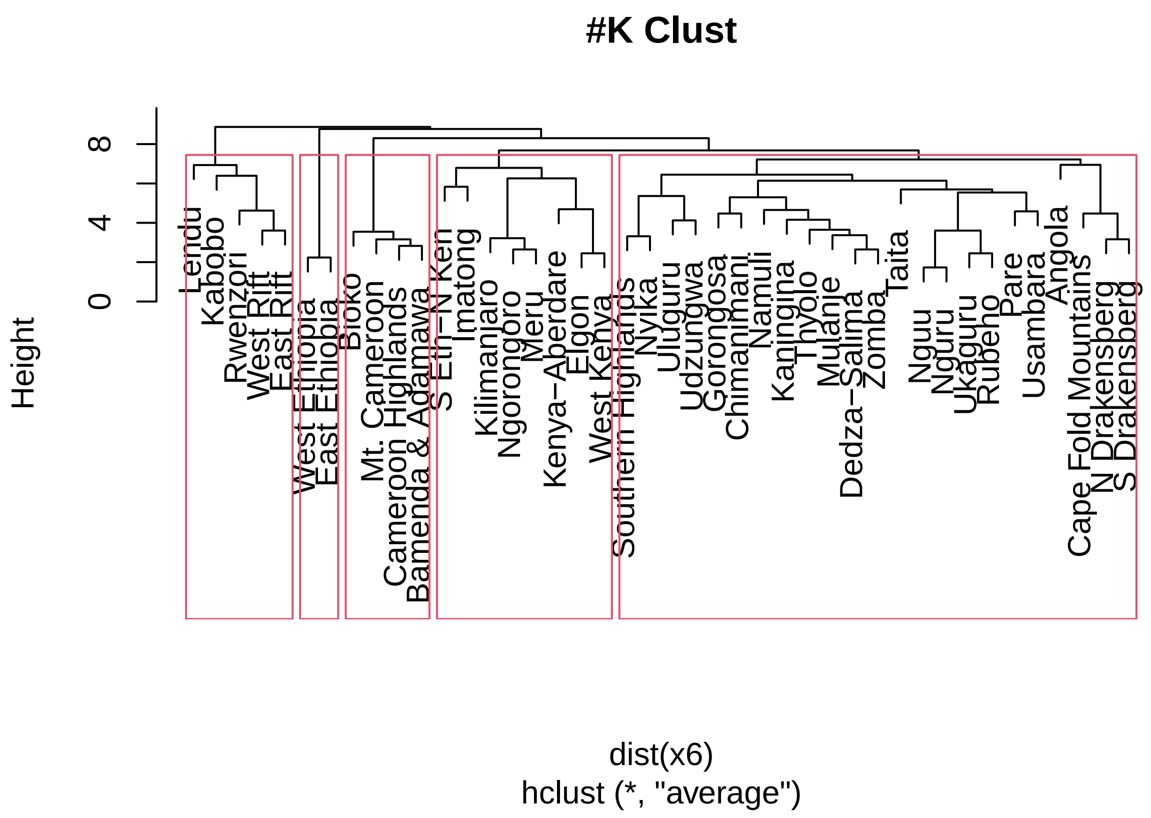
#K Clust
Height
0
4
8
Lendu
Kabobo
Rwenzori
West Rift
East Rift
West Ethiopia
East Ethiopia
Bioko
Mt. Cameroon
Cameroon Highlands
Bamenda & Adamawa
S Eth−N Ken
Imatong
Kilimanjaro
Ngorongoro
Meru
Kenya−Aberdare
Elgon
West Kenya
Southern Highlands
Nyika
Uluguru
Udzungwa
Gorongosa
Chimanimani
Namuli
Kaningina
Thyolo
Mulanje
Dedza−Salima
Zomba
Taita
Nguu
Nguru
Ukaguru
Rubeho
Pare
Usambara
Angola
Cape Fold Mountains
N Drakensberg
S Drakensberg
dist(x6)
hclust (*, "average")

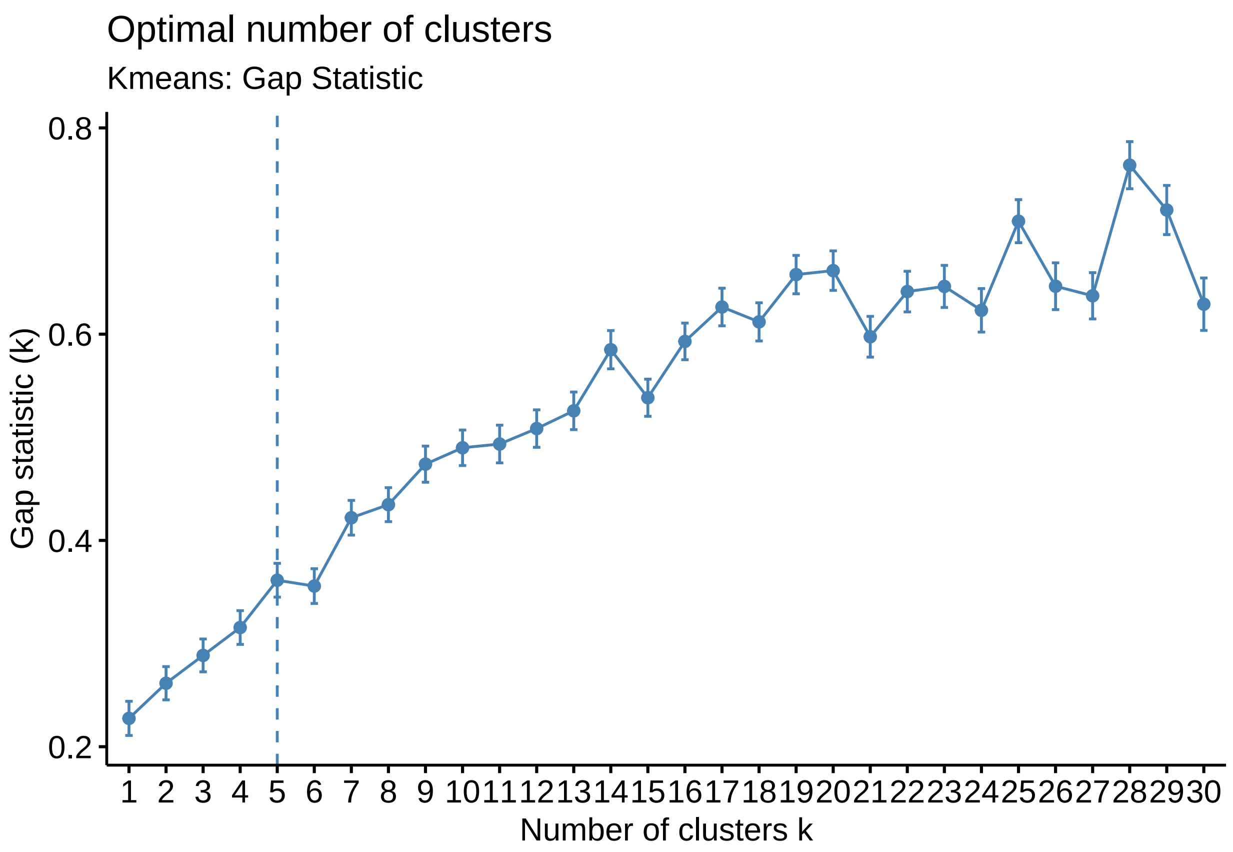

2.5 Group

```
clustertaxa(level="Group",xdata=xdata,
            ncluster=16,hcluster=4,
            author="Cooper")
```

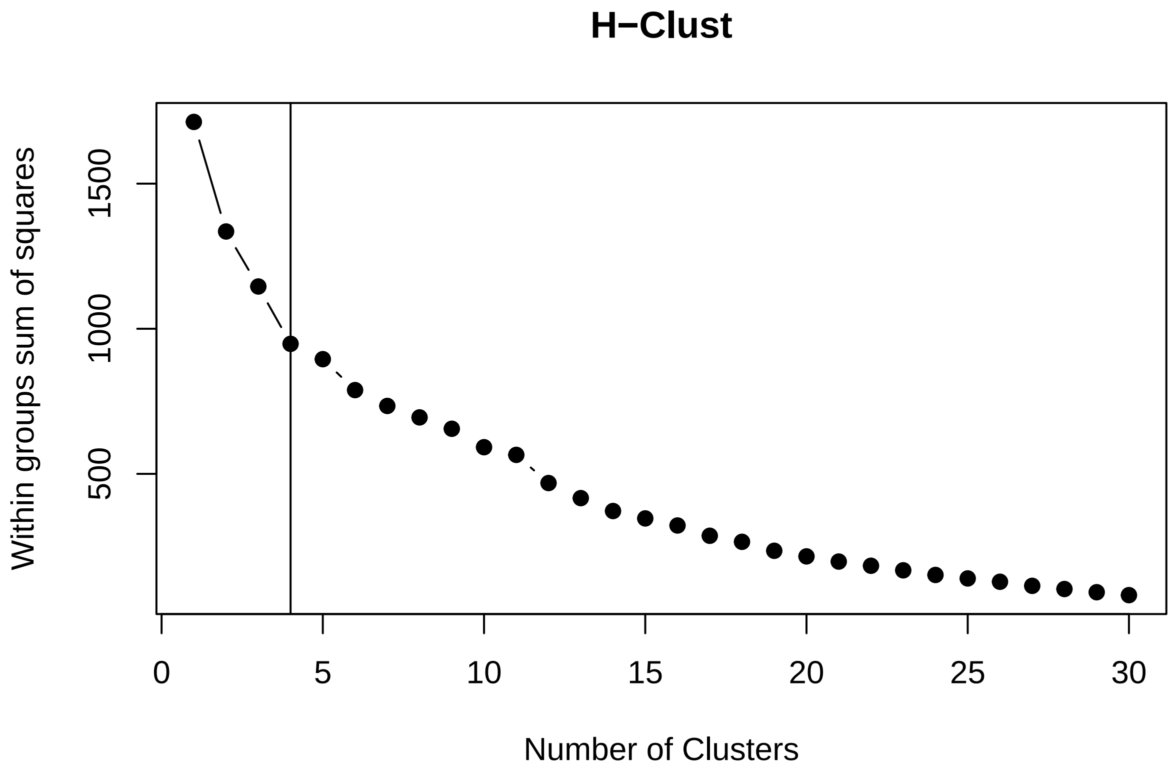
H−Clust
Within groups sum of squares
500
1000
1500
0
5
10
15
20
25
30
Number of Clusters

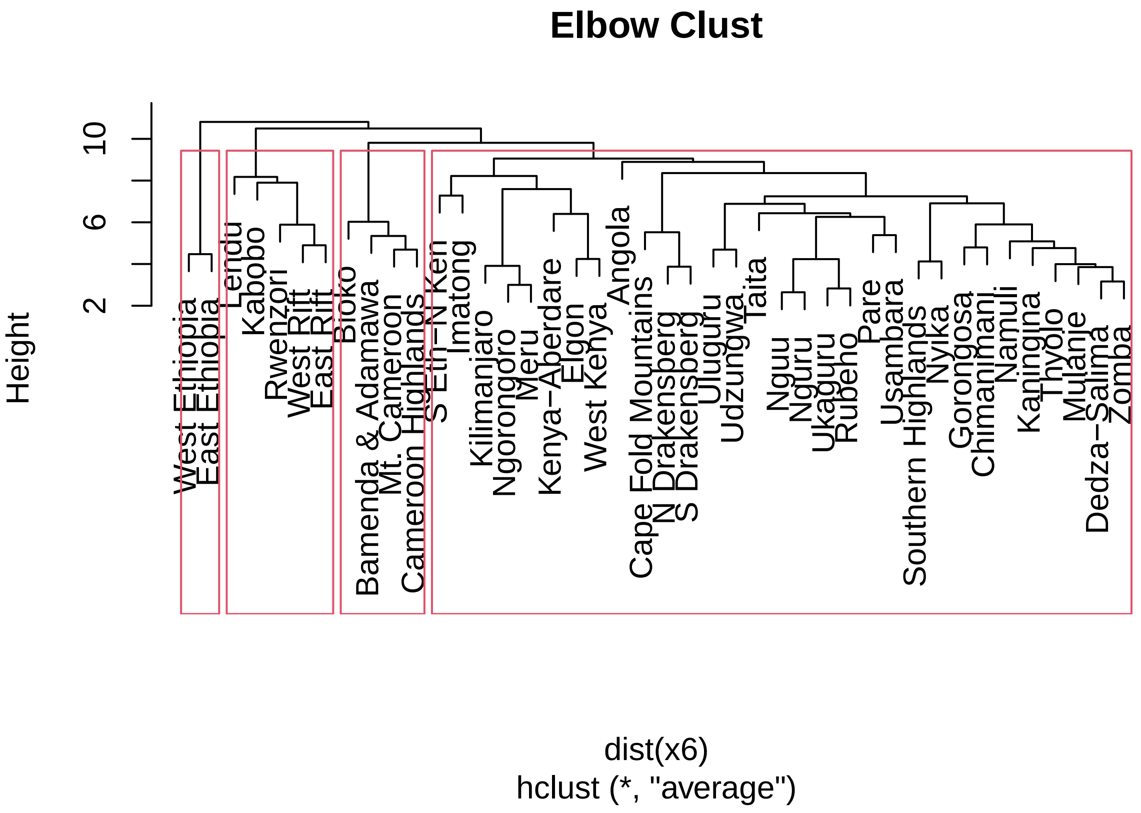

Elbow Clust
Height
10
6
2
West Ethiopia
East Ethiopia
Lendu
Kabobo
Rwenzori
West Rift
East Rift
Bioko
Bamenda & Adamawa
Mt. Cameroon
Cameroon Highlands
S Eth−N Ken
Imatong
Kilimanjaro
Ngorongoro
Meru
Kenya−Aberdare
Elgon
West Kenya
Angola
Cape Fold Mountains
N Drakensberg
S Drakensberg
Uluguru
Udzungwa
Taita
Nguu
Nguru
Ukaguru
Rubeho
Pare
Usambara
Southern Highlands
Nyika
Gorongosa
Chimanimani
Namuli
Kaningina
Thyolo
Mulanje
Dedza−Salima
Zomba
dist(x6)
hclust (*, "average")

#K Clust

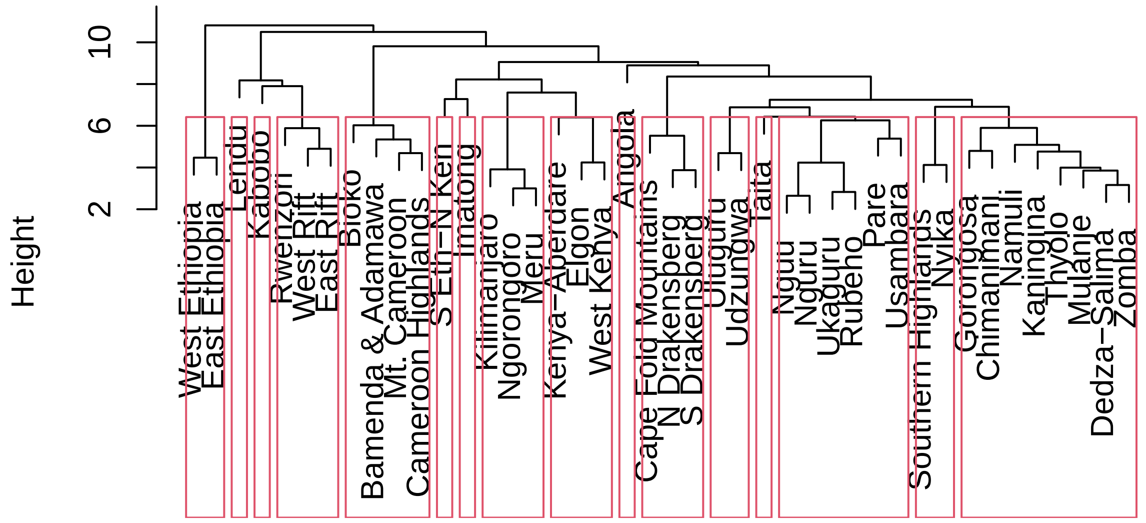

dist(x6)
hclust (*, "average")

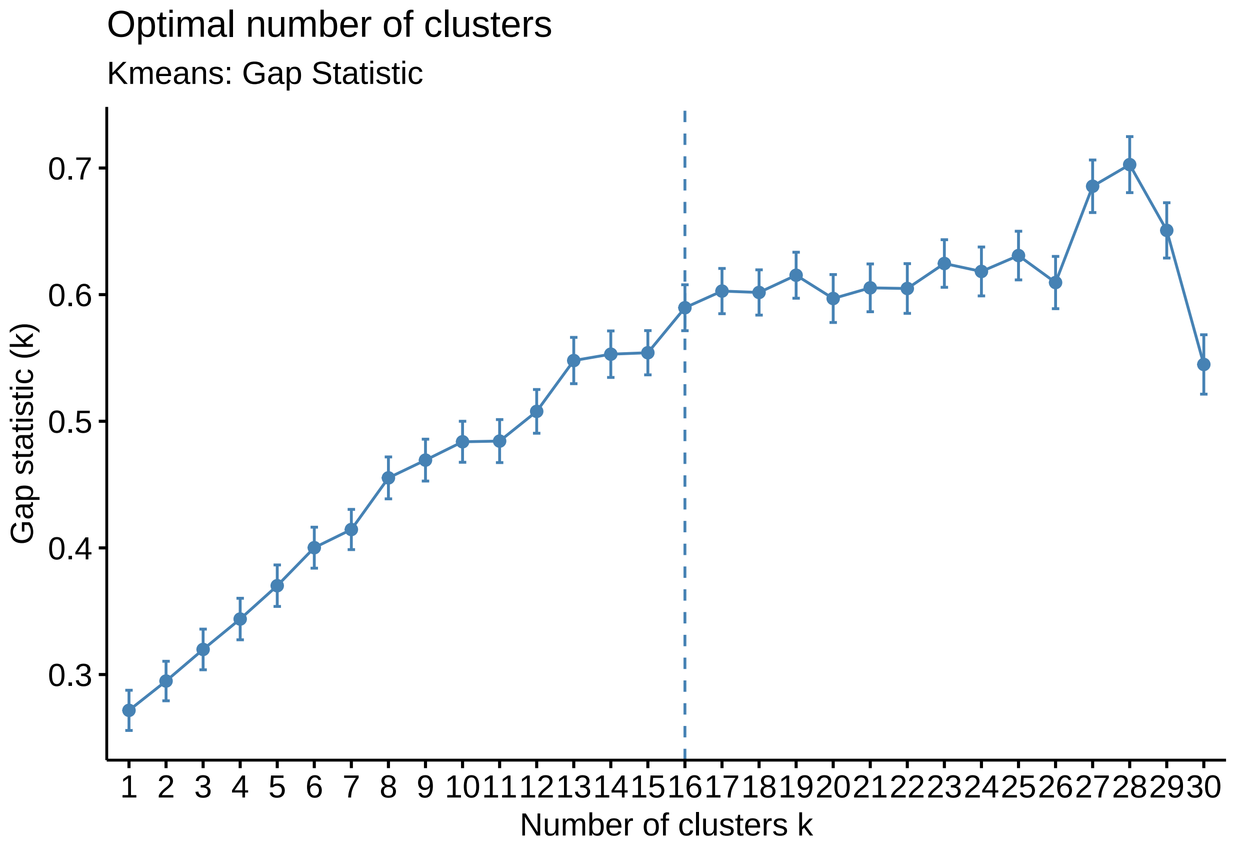


```
clustertaxa(level="Group",xdata=bowie.data,
            ncluster=3,hcluster=6,
            author="Bowie")
```

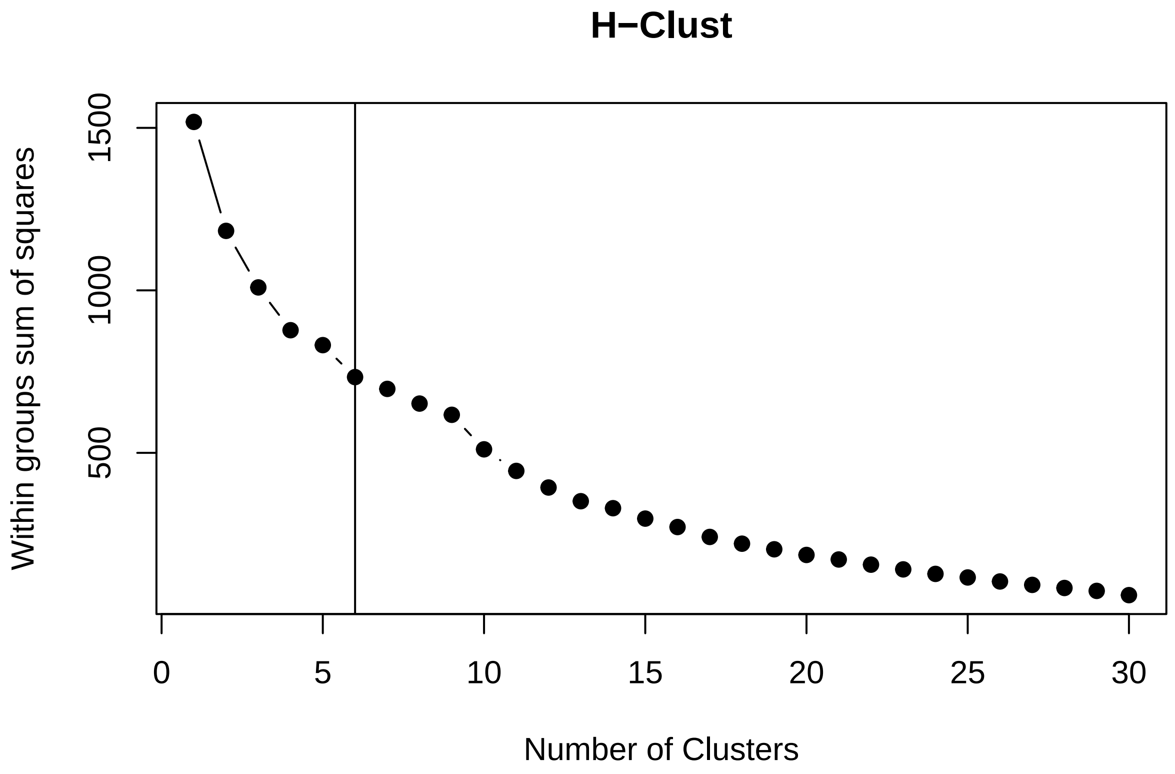
H−Clust
Within groups sum of squares
1500
1000
500
0
5
10
15
20
25
30
Number of Clusters

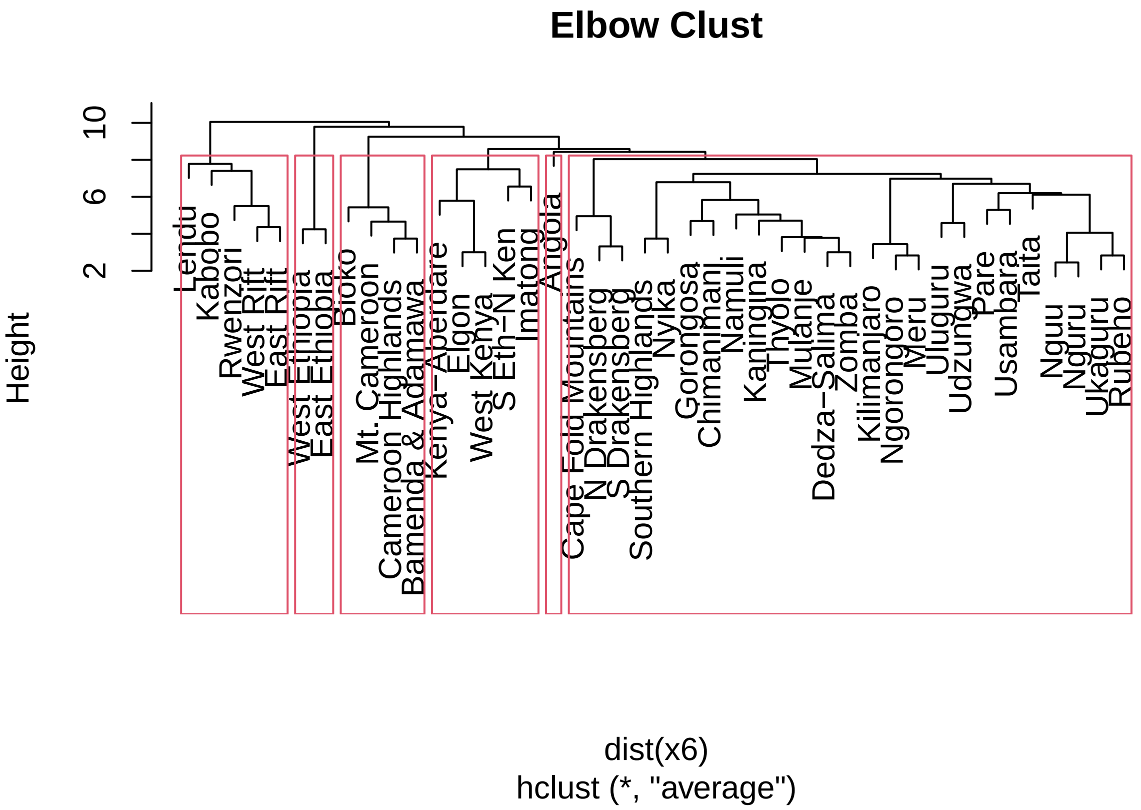
Elbow Clust
Height
10
6
2
Lendu
Kabobo
Rwenzori
West Rift
East Rift
West Ethiopia
East Ethiopia
Bioko
Mt. Cameroon
Cameroon Highlands
Bamenda & Adamawa
Kenya−Aberdare
Elgon
West Kenya
S Eth−N Ken
Imatong
Angola
Cape Fold Mountains
N Drakensberg
S Drakensberg
Southern Highlands
Nyika
Gorongosa
Chimanimani
Namuli
Kaningina
Thyolo
Mulanje
Dedza−Salima
Zomba
Kilimanjaro
Ngorongoro
Meru
Uluguru
Udzungwa
Pare
Usambara
Taita
Nguu
Nguru
Ukaguru
Rubeho
dist(x6)
hclust (*, "average")

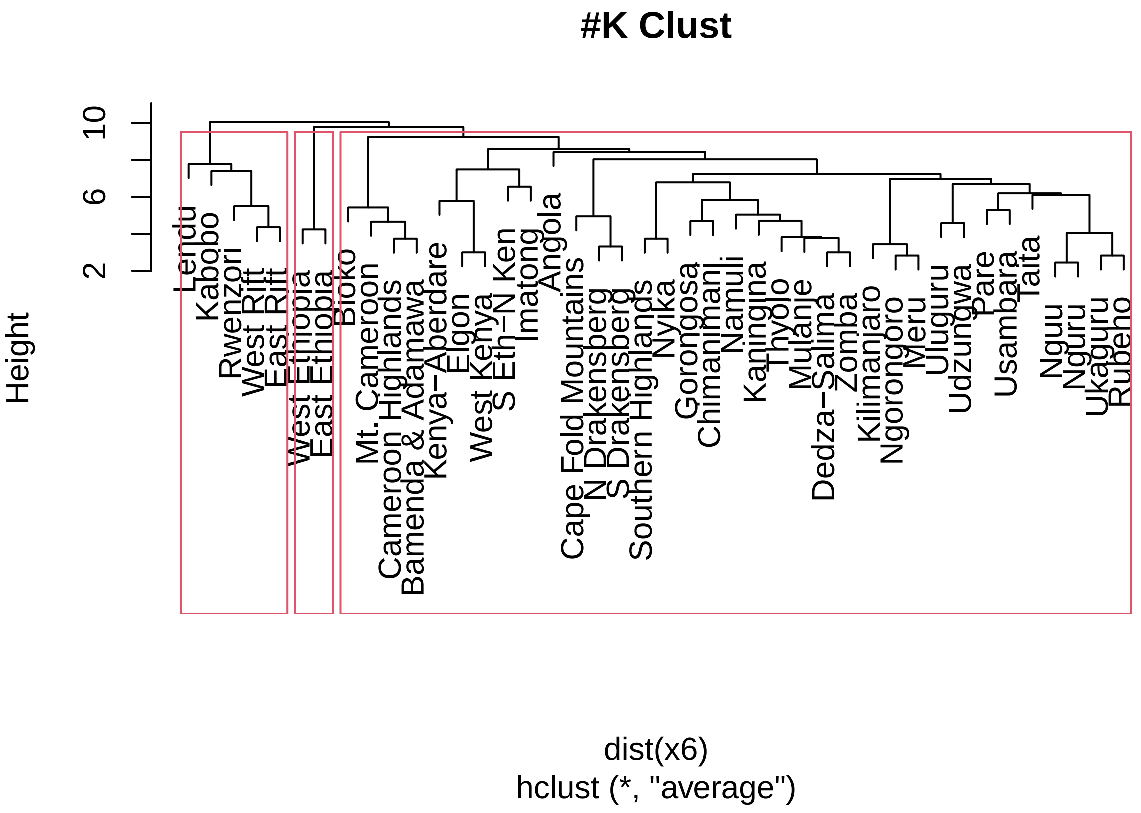
#K Clust
Height
2
6
10
Lendu
Kabobo
Rwenzori
West Rift
East Rift
West Ethiopia
East Ethiopia
Bioko
Mt. Cameroon
Cameroon Highlands
Bamenda & Adamawa
Kenya−Aberdare
Elgon
West Kenya
S Eth−N Ken
Imatong
Angola
Cape Fold Mountains
N Drakensberg
S Drakensberg
Southern Highlands
Nyika
Gorongosa
Chimanimani
Namuli
Kaningina
Thyolo
Mulanje
Dedza−Salima
Zomba
Kilimanjaro
Ngorongoro
Meru
Uluguru
Udzungwa
Pare
Usambara
Taita
Nguu
Nguru
Ukaguru
Rubeho
dist(x6)
hclust (*, "average")

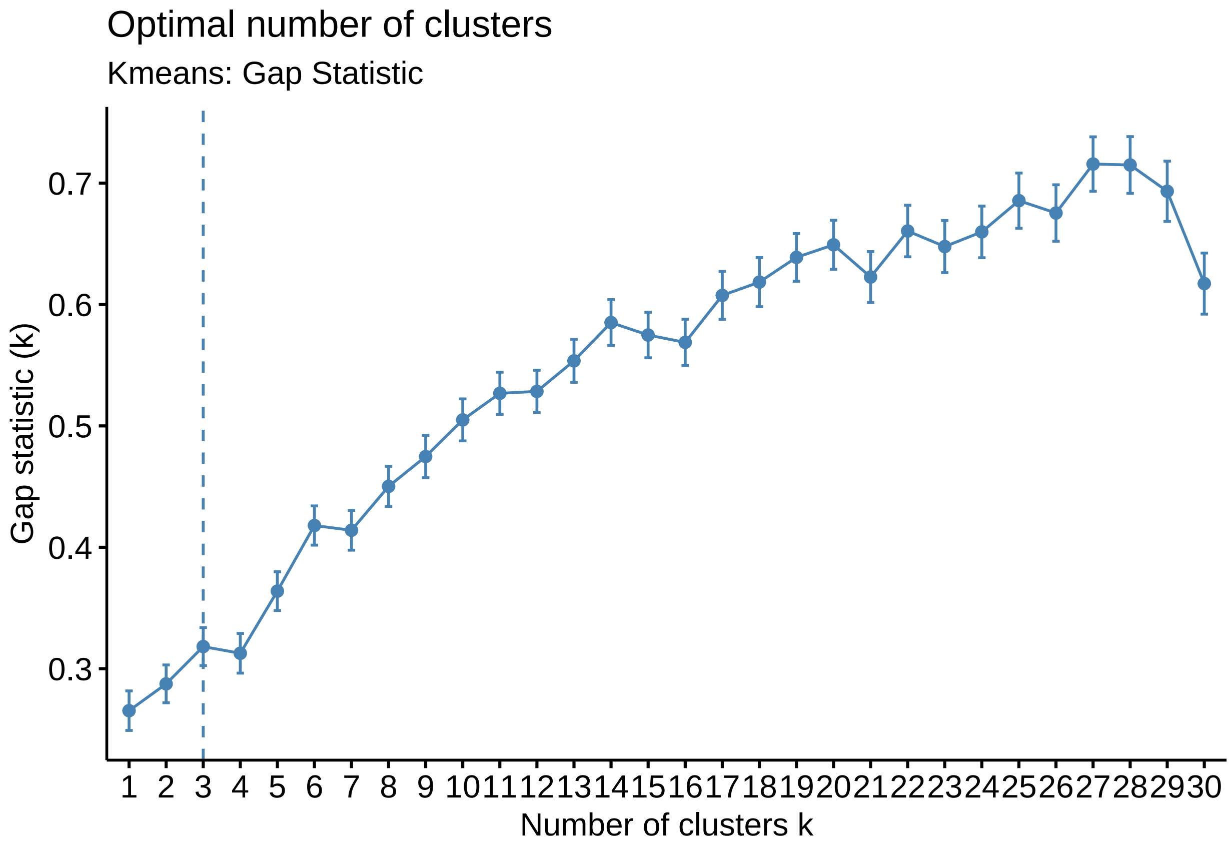


```
clustertaxa(level="Group",xdata=dowsett.data,
            ncluster=10,hcluster=5,
            author="Dowsett")
```

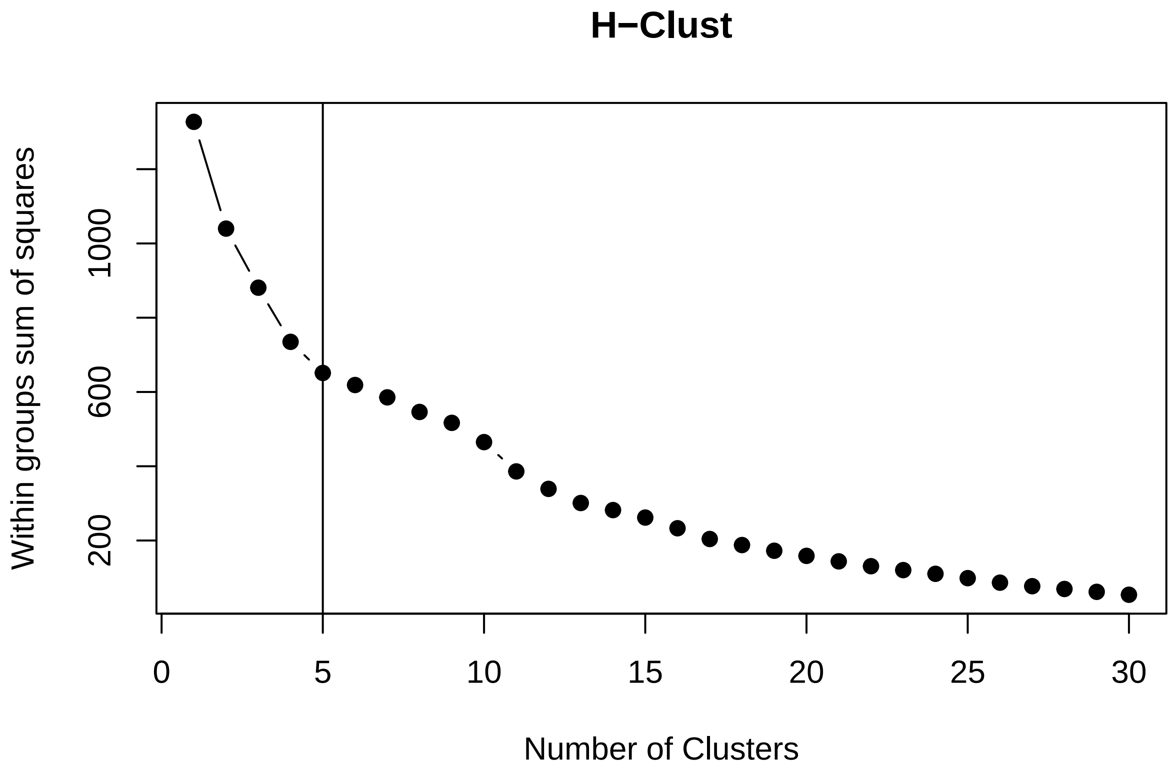
H−Clust
Within groups sum of squares
200
600
1000
0
5
10
15
20
25
30
Number of Clusters

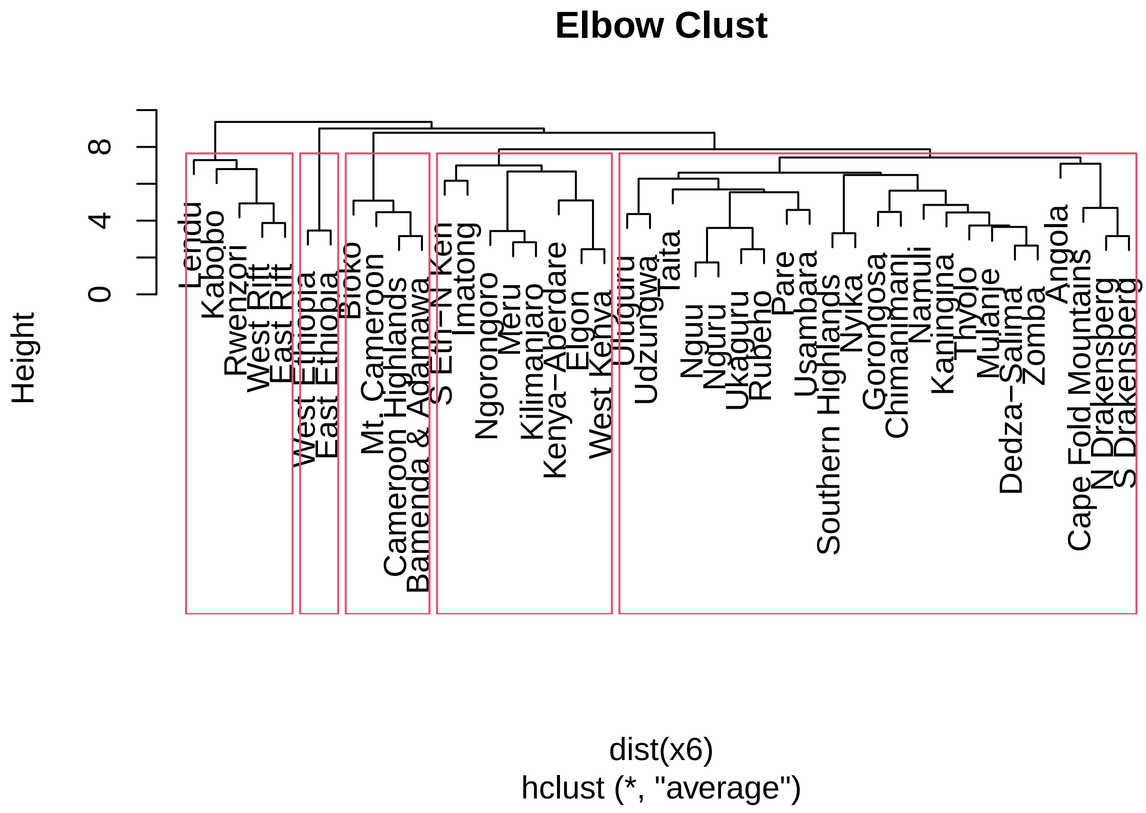

Elbow Clust
Height
0
4
8
Lendu
Kabobo
Rwenzori
West Rift
East Rift
West Ethiopia
East Ethiopia
Bioko
Mt. Cameroon
Cameroon Highlands
Bamenda & Adamawa
S Eth−N Ken
Imatong
Ngorongoro
Meru
Kilimanjaro
Kenya−Aberdare
Elgon
West Kenya
Uluguru
Udzungwa
Taita
Nguu
Nguru
Ukaguru
Rubeho
Pare
Usambara
Southern Highlands
Nyika
Gorongosa
Chimanimani
Namuli
Kaningina
Thyolo
Mulanje
Dedza−Salima
Zomba
Angola
Cape Fold Mountains
N Drakensberg
S Drakensberg
dist(x6)
hclust (*, "average")

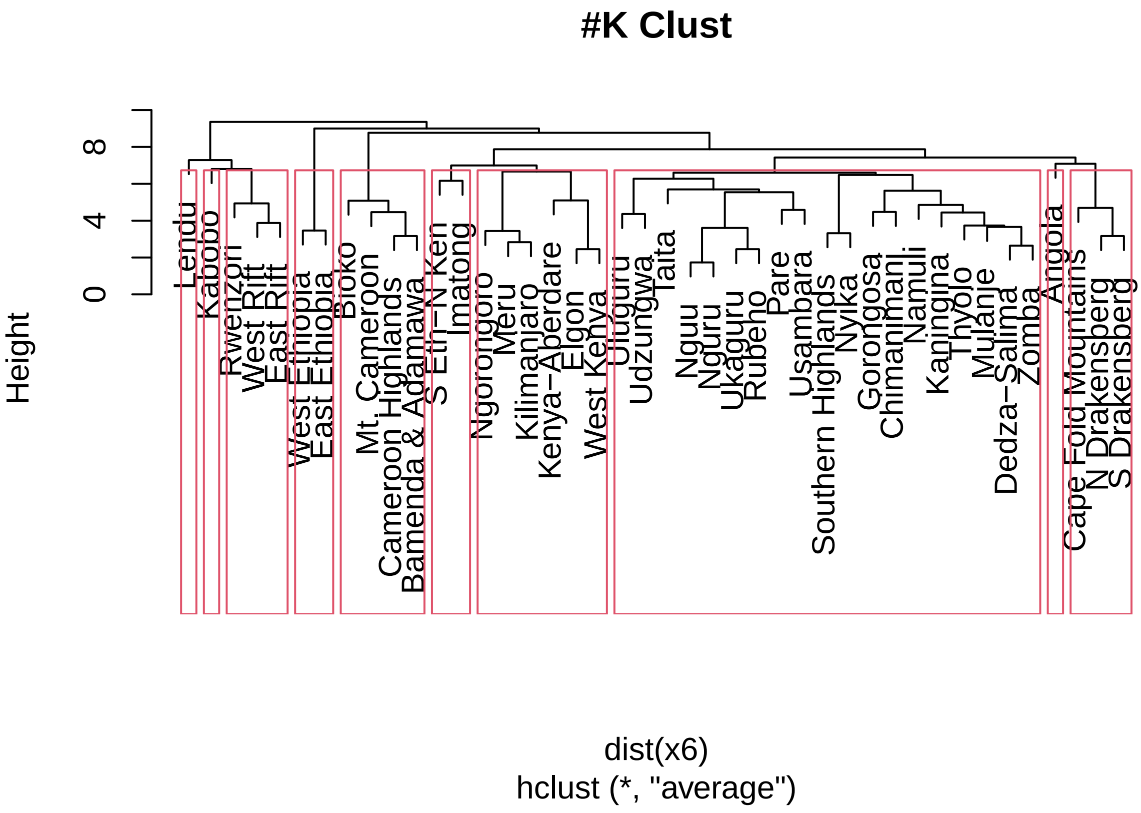
#K Clust
Height
0
4
8
Lendu
Kabobo
Rwenzori
West Rift
East Rift
West Ethiopia
East Ethiopia
Bioko
Mt. Cameroon
Cameroon Highlands
Bamenda & Adamawa
S Eth–N Ken
Imatong
Ngorongoro
Meru
Kilimanjaro
Kenya–Aberdare
Elgon
West Kenya
Uluguru
Udzungwa
Taita
Nguu
Nguru
Ukaguru
Rubeho
Pare
Usambara
Southern Highlands
Nyika
Gorongosa
Chimanimani
Namuli
Kaningina
Thyolo
Mulanje
Dedza–Salima
Zomba
Angola
Cape Fold Mountains
N Drakensberg
S Drakensberg
dist(x6)
hclust (*, "average")

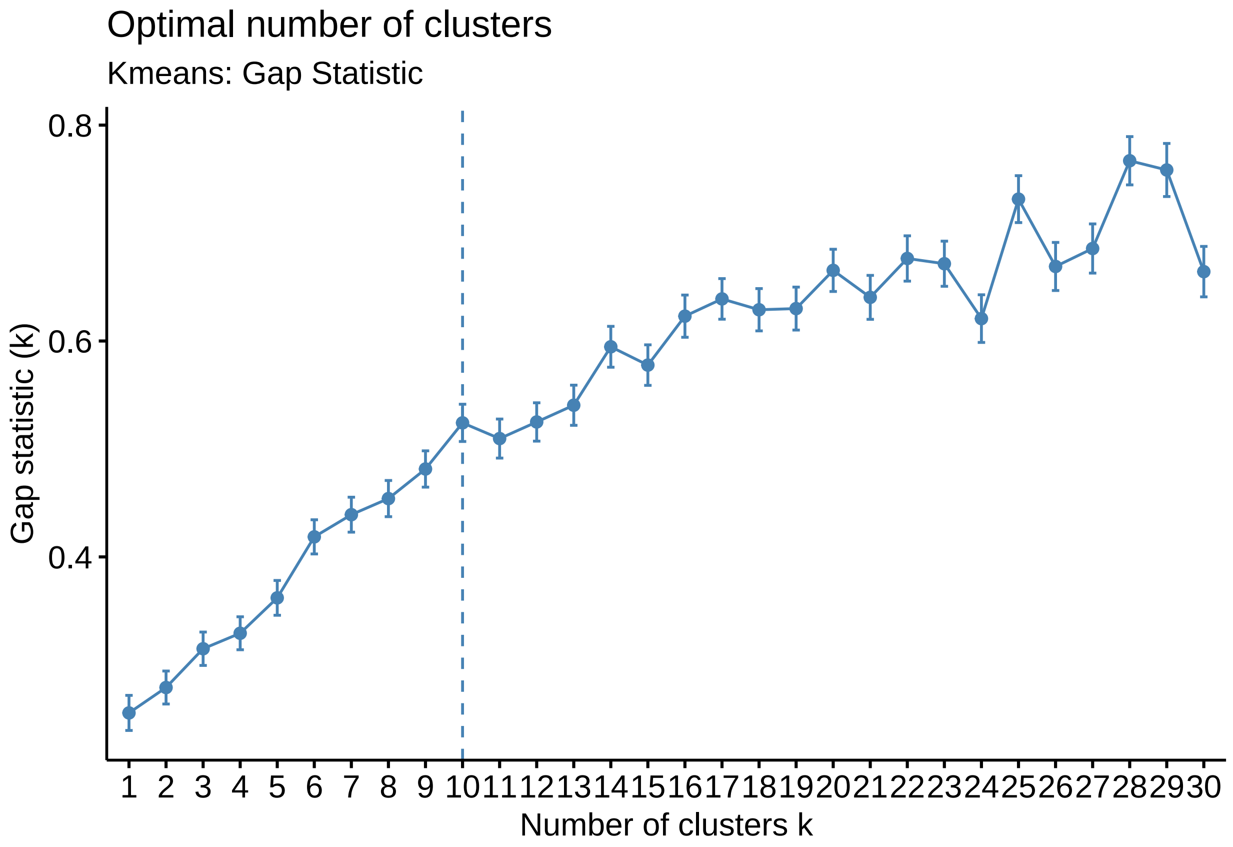

2.6 Subspecies

```
clustertaxa(level="Subspecies",xdata=xdata,
            ncluster=8,hcluster=6,
            author="Cooper")
```

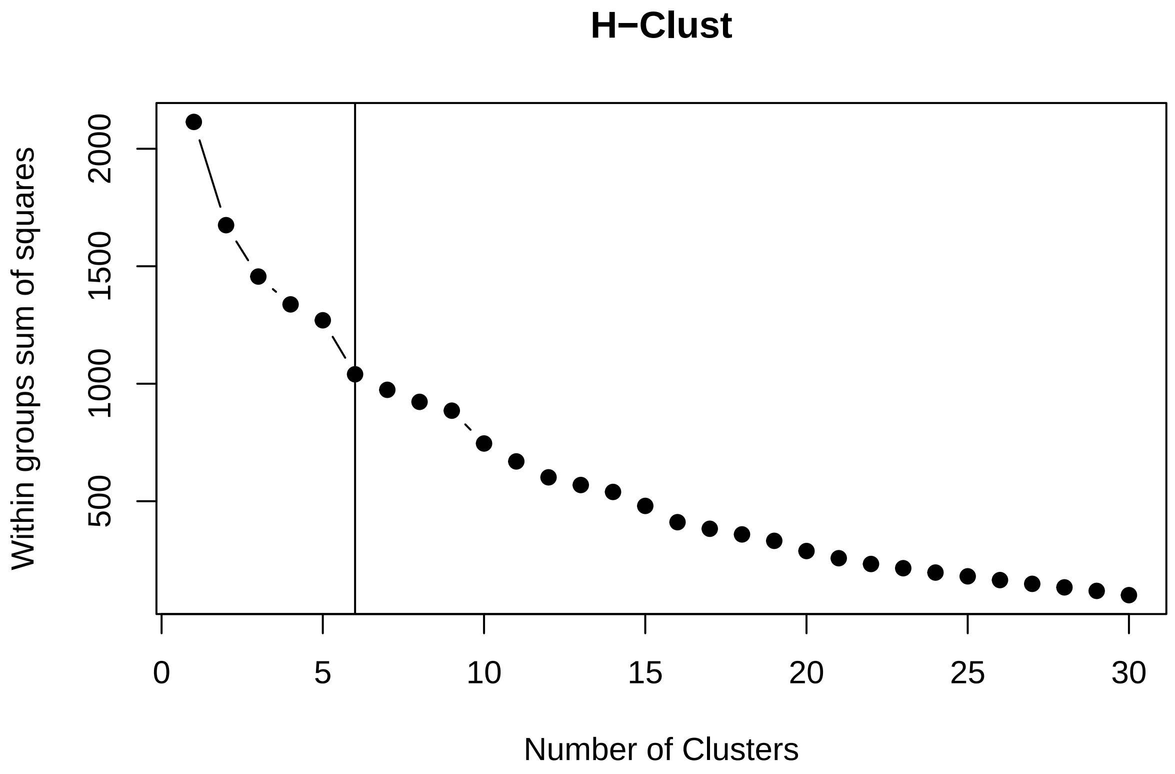
H−Clust
Within groups sum of squares
500
1000
1500
2000
0
5
10
15
20
25
30
Number of Clusters

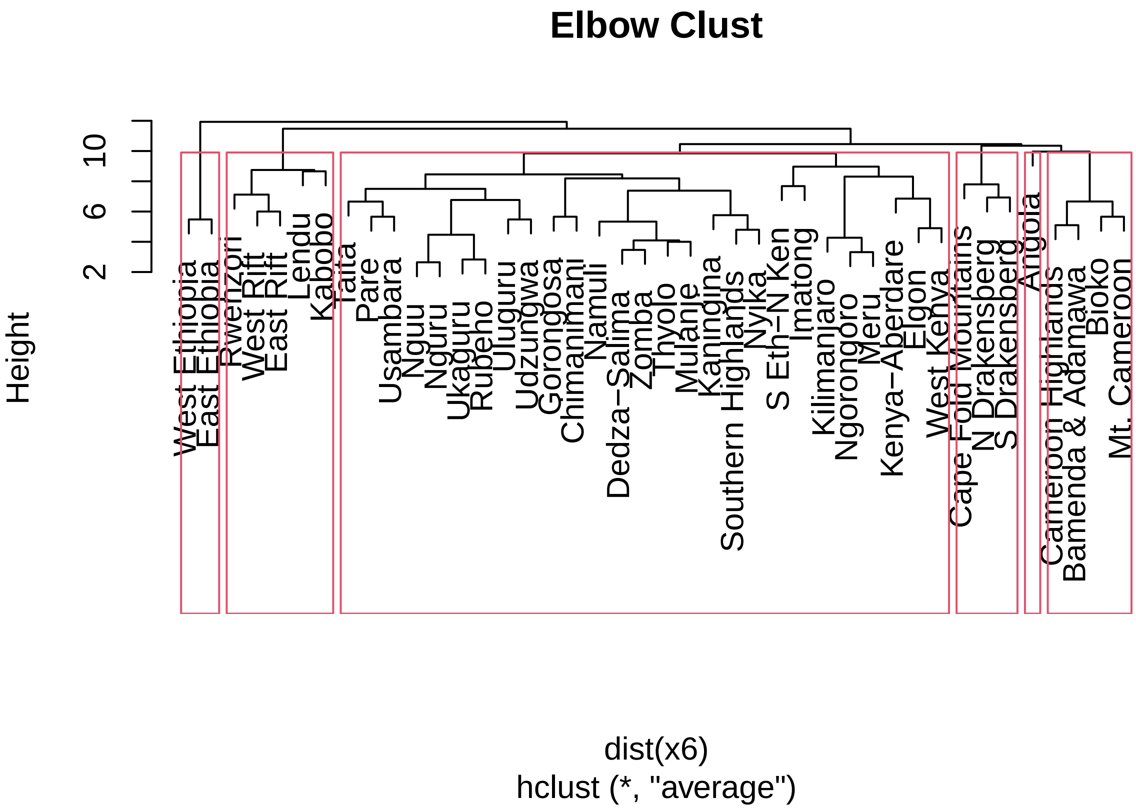
Elbow Clust
Height
10
6
2
West Ethiopia
East Ethiopia
Rwenzori
West Rift
East Rift
Lendu
Kabobo
Taita
Pare
Usambara
Nguu
Nguru
Ukaguru
Rubeho
Uluguru
Udzungwa
Gorongosa
Chimanimani
Namuli
Dedza−Salima
Zomba
Thyolo
Mulanje
Kaningina
Southern Highlands
Nyika
S Eth−N Ken
Imatong
Kilimanjaro
Ngorongoro
Meru
Kenya−Aberdare
Elgon
West Kenya
Cape Fold Mountains
N Drakensberg
S Drakensberg
Angola
Cameroon Highlands
Bamenda & Adamawa
Bioko
Mt. Cameroon
dist(x6)
hclust (*, "average")

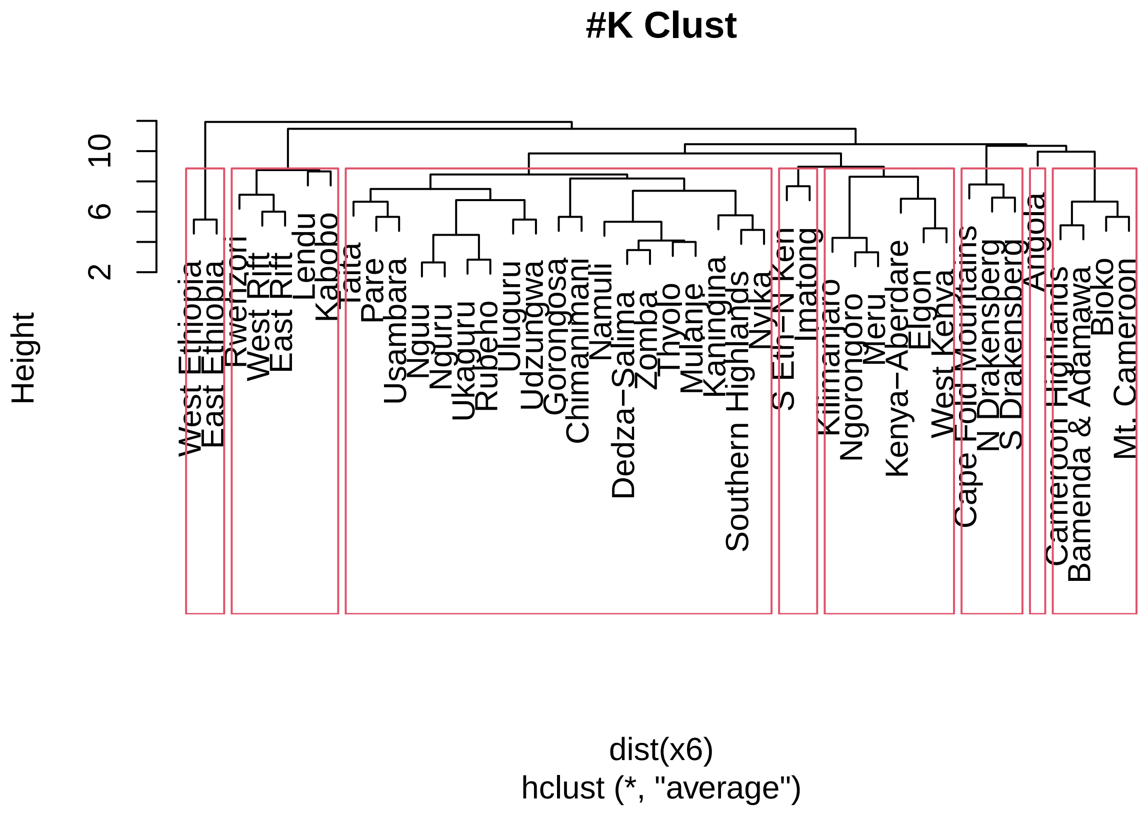
#K Clust
Height
2
6
10
West Ethiopia
East Ethiopia
Rwenzori
West Rift
East Rift
Lendu
Kabobo
Taita
Pare
Usambara
Nguu
Nguru
Ukaguru
Rubeho
Uluguru
Udzungwa
Gorongosa
Chimanimani
Namuli
Dedza−Salima
Zomba
Thyolo
Mulanje
Kaningina
Southern Highlands
Nyika
S Eth−N Ken
Imatong
Kilimanjaro
Ngorongoro
Meru
Kenya−Aberdare
Elgon
West Kenya
Cape Fold Mountains
N Drakensberg
S Drakensberg
Angola
Cameroon Highlands
Bamenda & Adamawa
Bioko
Mt. Cameroon
dist(x6)
hclust (*, "average")

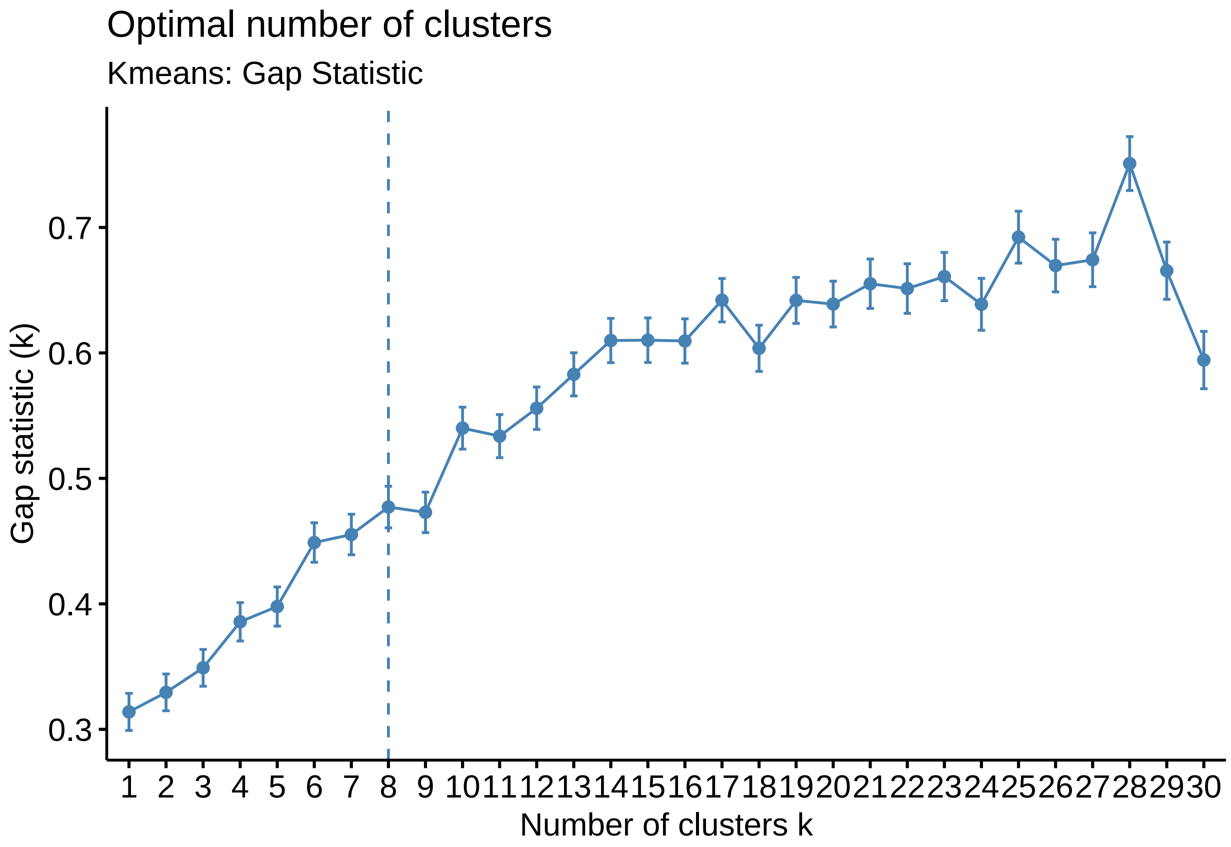


```
clustertaxa(level="Subspecies",xdata=bowie.data,
            ncluster=11,hcluster=8,
            author="Bowie")
```

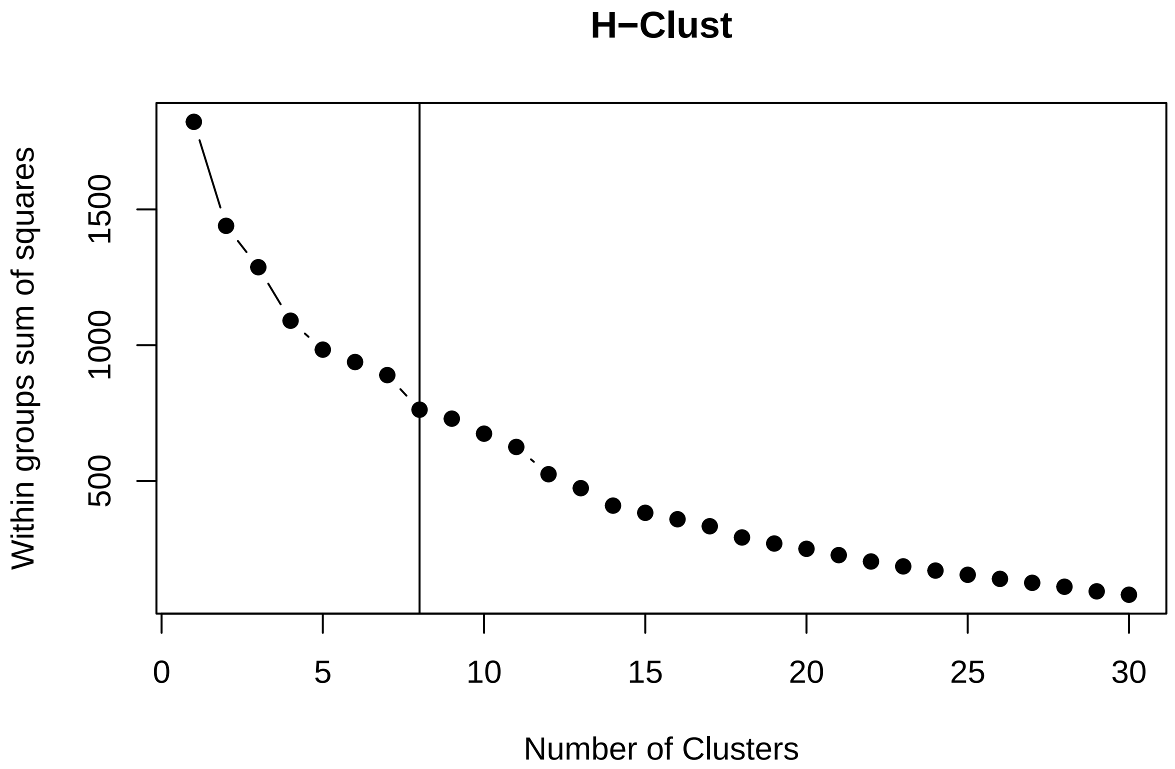
H−Clust
Within groups sum of squares
500
1000
1500
0
5
10
15
20
25
30
Number of Clusters

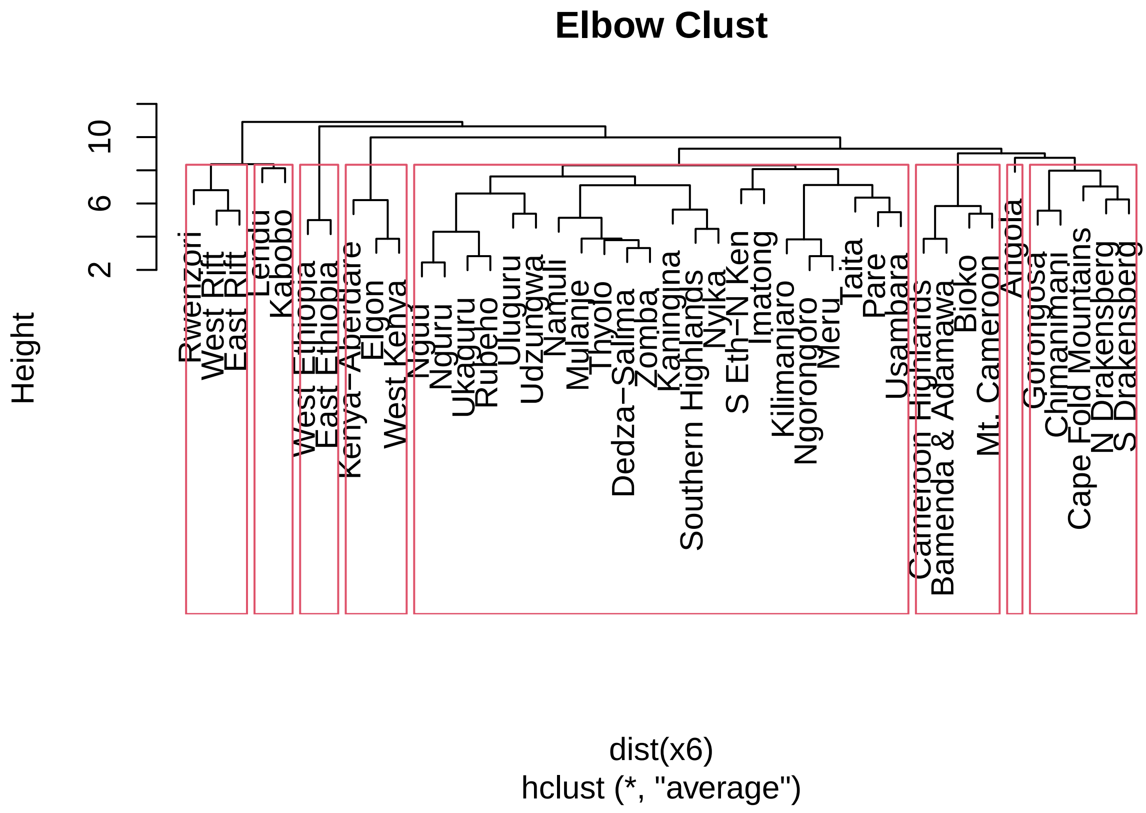

Elbow Clust
Height
2
6
10
Rwenzori
West Rift
East Rift
Lendu
Kabobo
West Ethiopia
East Ethiopia
Kenya−Aberdare
Elgon
West Kenya
Nguu
Nguru
Ukaguru
Rubeho
Uluguru
Udzungwa
Namuli
Mulanje
Thyolo
Dedza−Salima
Zomba
Kaningina
Southern Highlands
Nyika
S Eth−N Ken
Imatong
Kilimanjaro
Ngorongoro
Meru
Taita
Pare
Usambara
Cameroon Highlands
Bamenda & Adamawa
Bioko
Mt. Cameroon
Angola
Gorongosa
Chimanimani
Cape Fold Mountains
N Drakensberg
S Drakensberg
dist(x6)
hclust (*, "average")

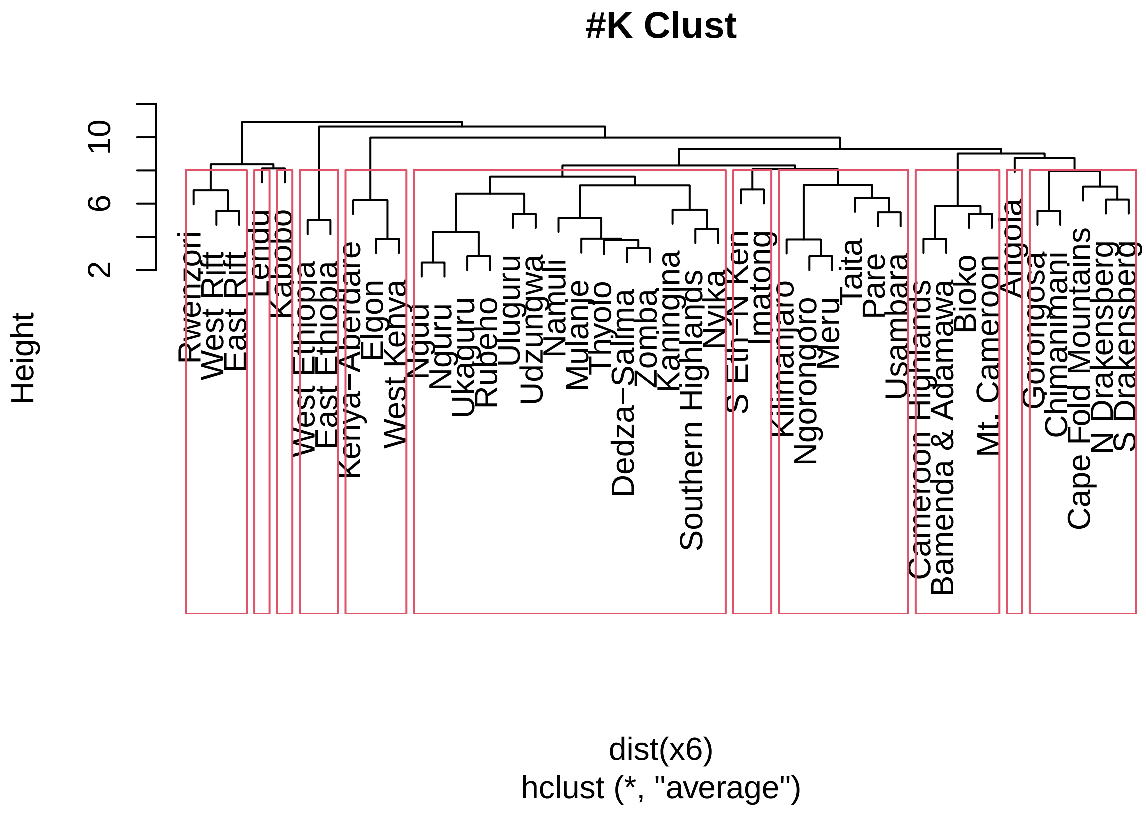
#K Clust
Height
2
6
10
Rwenzori
West Rift
East Rift
Lendu
Kabobo
West Ethiopia
East Ethiopia
Kenya−Aberdare
Elgon
West Kenya
Nguu
Nguru
Ukaguru
Rubeho
Uluguru
Udzungwa
Namuli
Mulanje
Thyolo
Dedza−Salima
Zomba
Kaningina
Southern Highlands
Nyika
S Eth−N Ken
Imatong
Kilimanjaro
Ngorongoro
Meru
Taita
Pare
Usambara
Cameroon Highlands
Bamenda & Adamawa
Bioko
Mt. Cameroon
Angola
Gorongosa
Chimanimani
Cape Fold Mountains
N Drakensberg
S Drakensberg
dist(x6)
hclust (*, "average")

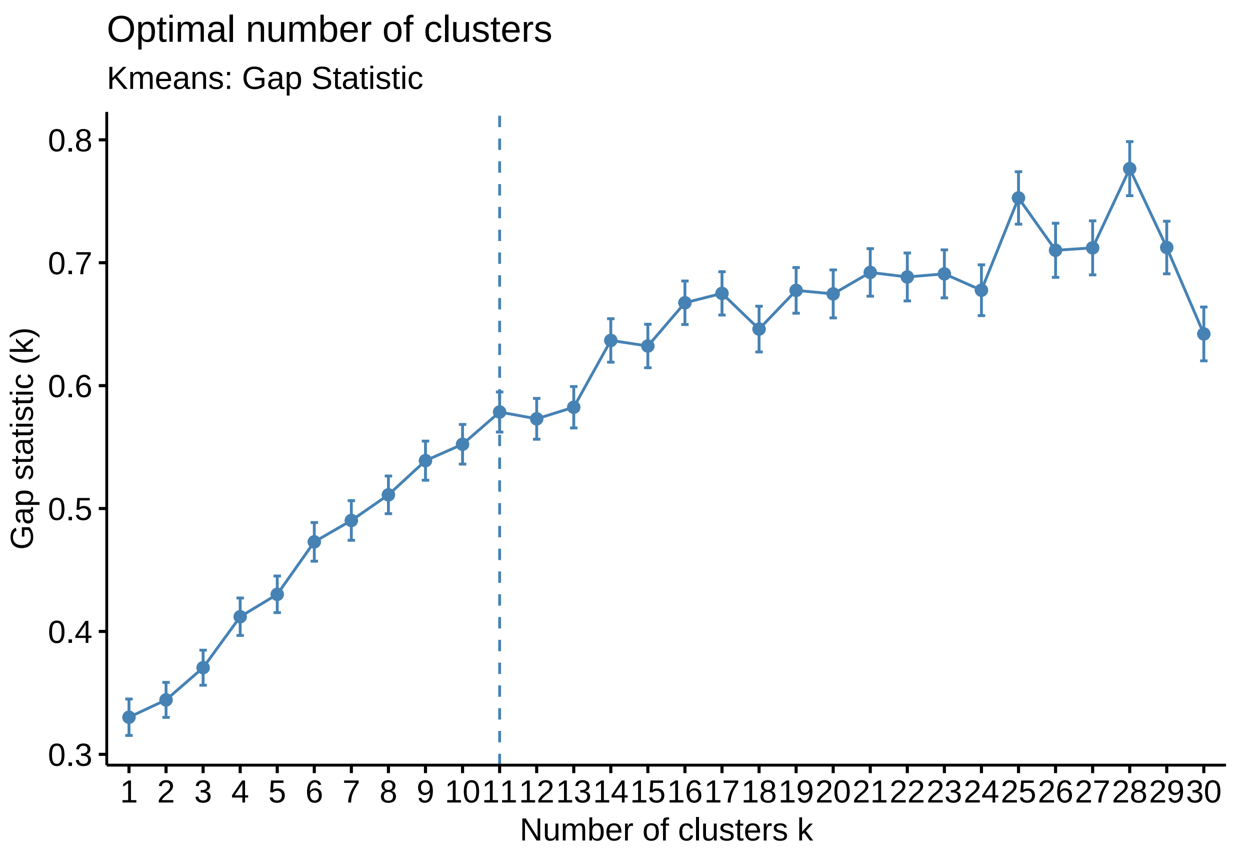


```
clustertaxa(level="Subspecies",xdata=dowsett.data,
            ncluster=12,hcluster=5,
            author="Dowsett")
```

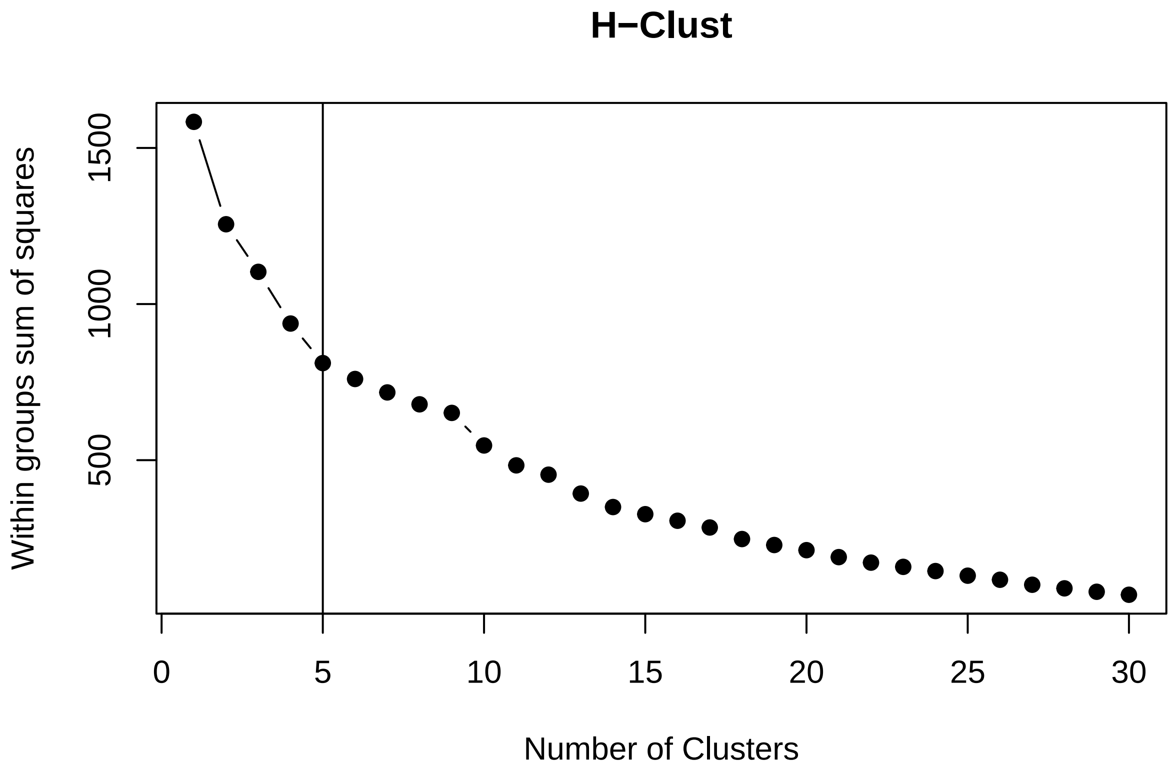
H−Clust
Within groups sum of squares
500
1000
1500
0
5
10
15
20
25
30
Number of Clusters

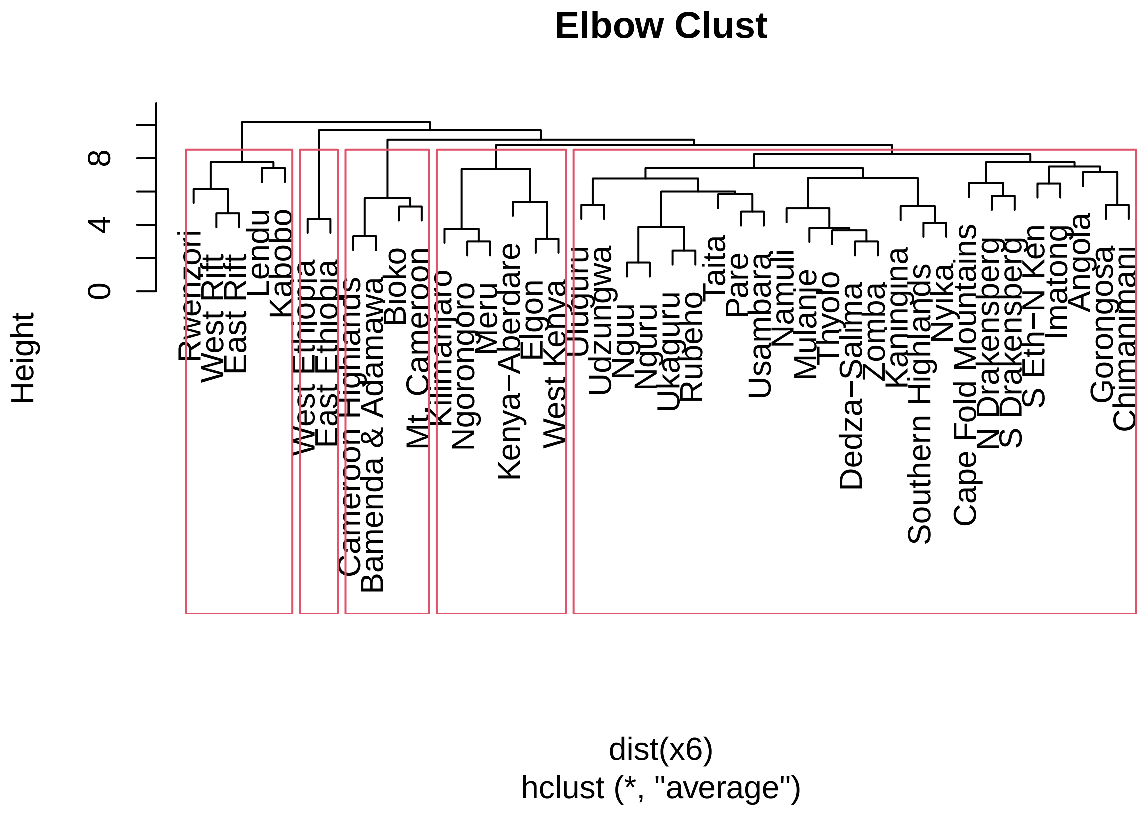
Elbow Clust
Height
0
4
8
Rwenzori
West Rift
East Rift
Lendu
Kabobo
West Ethiopia
East Ethiopia
Cameroon Highlands
Bamenda & Adamawa
Bioko
Mt. Cameroon
Kilimanjaro
Ngorongoro
Meru
Kenya−Aberdare
Elgon
West Kenya
Uluguru
Udzungwa
Nguu
Nguru
Ukaguru
Rubeho
Taita
Pare
Usambara
Namuli
Mulanje
Thyolo
Dedza−Salima
Zomba
Kaningina
Southern Highlands
Nyika
Cape Fold Mountains
N Drakensberg
S Drakensberg
S Eth−N Ken
Imatong
Angola
Gorongosa
Chimanimani
dist(x6)
hclust (*, "average")

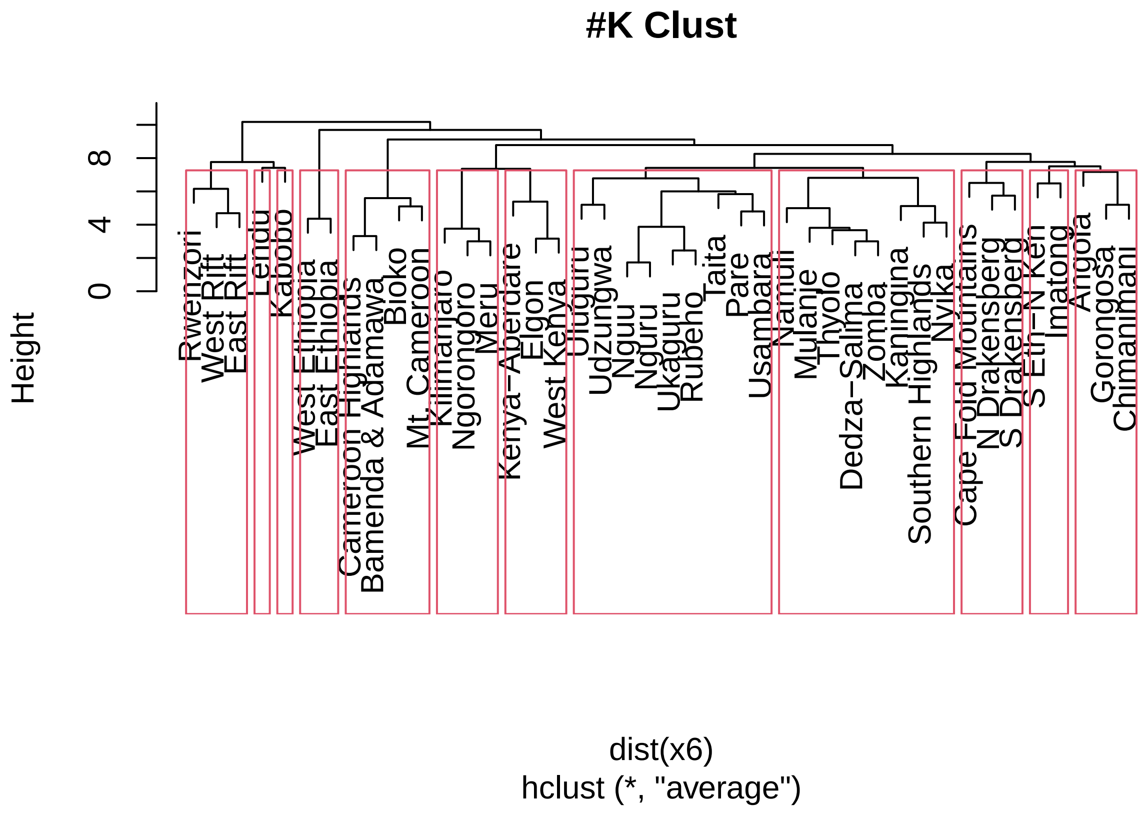
#K Clust
Height
0
4
8
Rwenzori
West Rift
East Rift
Lendu
Kabobo
West Ethiopia
East Ethiopia
Cameroon Highlands
Bamenda & Adamawa
Bioko
Mt. Cameroon
Kilimanjaro
Ngorongoro
Meru
Kenya−Aberdare
Elgon
West Kenya
Uluguru
Udzungwa
Nguu
Nguru
Ukaguru
Rubeho
Taita
Pare
Usambara
Namuli
Mulanje
Thyolo
Dedza−Salima
Zomba
Kaningina
Southern Highlands
Nyika
Cape Fold Mountains
N Drakensberg
S Drakensberg
S Eth−N Ken
Imatong
Angola
Gorongosa
Chimanimani
dist(x6)
hclust (*, "average")

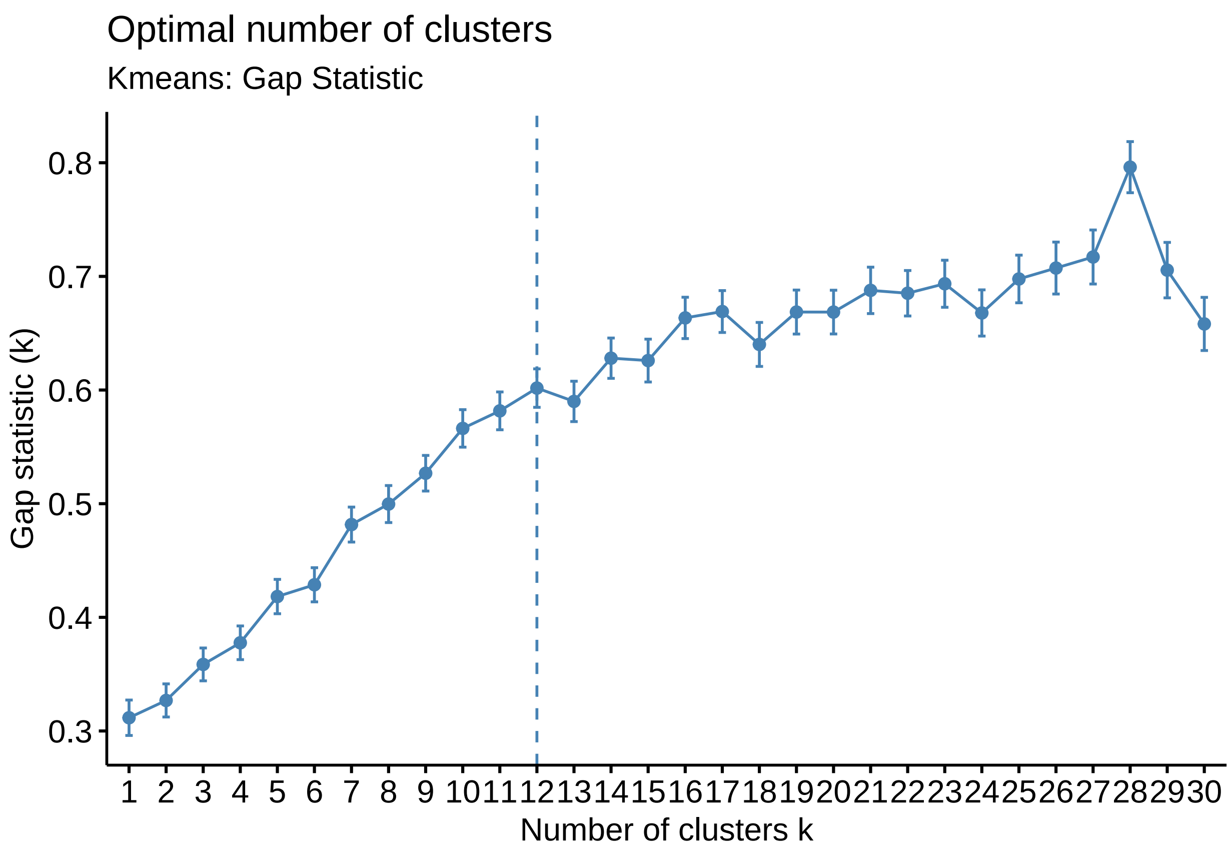

2.7 Number of groups across assessments

The following is the "ideal" number of groups (partitions) within each dendrogram for each taxonomic treatise.

KMeans

Source	Genus	Superspecies	Species	Group	Subspecies	μ	σ
Dowsett (1986)	4	13	5	10	12	8.8	4.1
Bowie (2003)	4	20	10	3	11	9.6	6.8
This study	10	10	10	16	8	10.8	3.0
Overall μ	6	14.3	8.3	9.6	10.3	9.73	
Overall σ	3.5	5.1	2.9	6.5	2.1		

```
Dowsett=cbind(1:length(Dowsett),"Dowsett",Dowsett)
Bowie=cbind(1:length(Bowie),"Bowie",Bowie)
This.study=cbind(1:length(This.study),"This study",This.study)

colnames(Dowsett)=colnames(Bowie)=colnames(This.study)=c("X","Study","N.Clusters")

dat.x=data.frame(rbind(Dowsett,Bowie,This.study))
```

```
a=ggplot(dat.x,aes(x=X,y=N.Clusters,group=Study))
b=geom_line(aes(linetype=Study))
```

```
b2=geom_point()
c=theme_classic()
d=ylim(0,22)
e=xlab('Level')
e2=ylab("# of Clusters")
e3=scale_x_discrete(breaks=c(1,2,3,4,5),labels=c("Genus","Superspecies","Species",
                             "Group","Subspecies"))

plot1=a+b+c+d+b2+e+e2+e3
print(plot1)
```

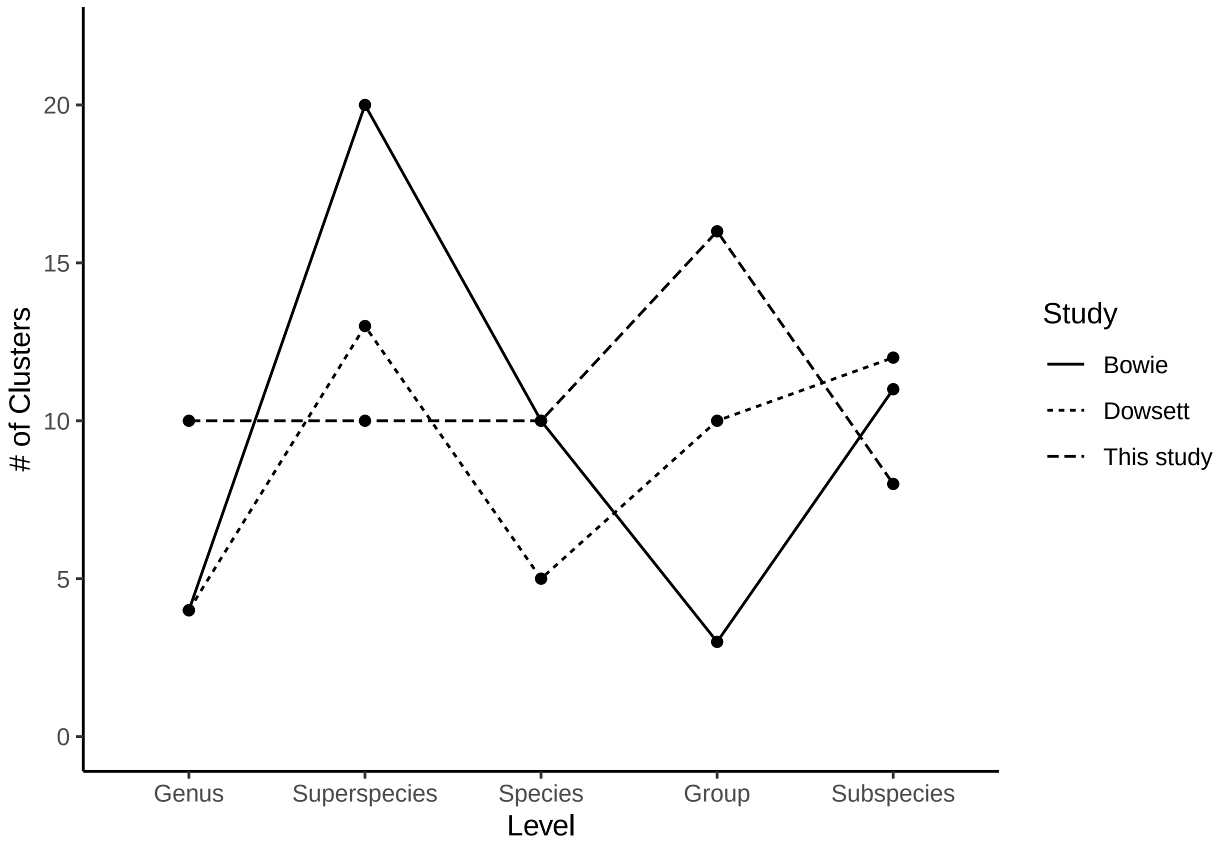


```
ggsave(plot1,filename = paste0(filepath,"number_groups.jpg"),dpi = 400)
```

```
## Saving 6.5 x 4.5 in image
```

Hierarchical Clustering

Note That this is a subjective measure.

Source	Genus	Superspecies	Species	Group	Subspecies	μ	σ
Dowsett (1986)	8	4	5	5	5	5.4	1.5
Bowie (2003)	7	5	4	6	8	6	1.6
This study	9	7	4	4	6	6	2.1
Overall μ	8.0	5.3	4,3	5.0	6.3	5.8	
Overall σ	1.0	1.5	0.6	1.0	1.5		

```
a=ggplot(dat.x,aes(x=X,y=N.Clusters,group=Study))
b=geom_line(aes(linetype=Study))
b2=geom_point()
c=theme_classic()
d=ylim(0,22)
e=xlab('Level')
e2=ylab("# of Clusters")
e3=scale_x_discrete(breaks=c(1,2,3,4,5),labels=c("Genus","Superspecies","Species",
                             "Group","Subspecies"))

plot1=a+b+c+d+b2+e+e2+e3
print(plot1)
```

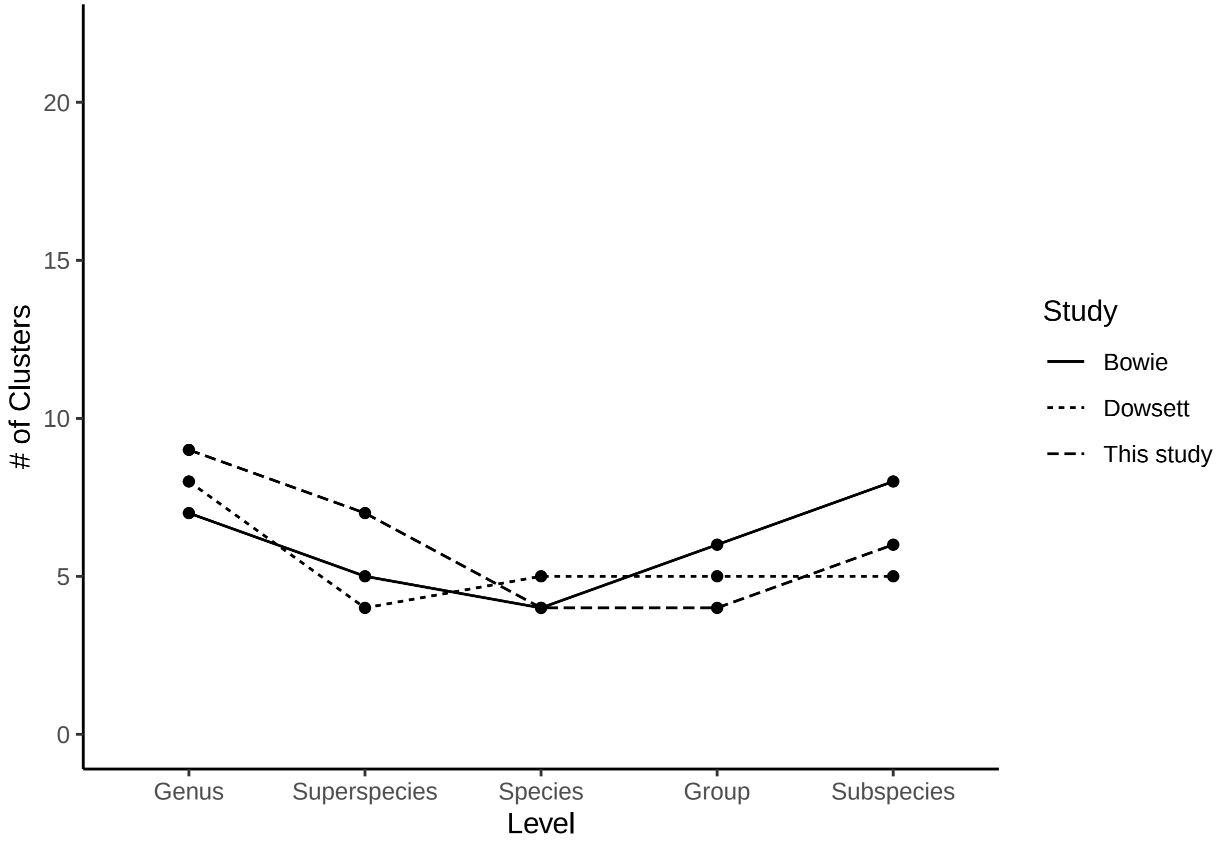


```
ggsave(plot1,filename = paste0(filepath,"number_groups.jpg"),dpi = 400)
```

```
## Saving 6.5 x 4.5 in image
```

Difference between Elbow and K-Means

Reported as $kmeans - hclust$, as the gap-statistic is a less fallible measure. Thus, positive numbers have higher numbers of clusters for *kmeans* and negative values have higher numbers of clusters for the elbow method. The difference between these values can fluctuate greatly between iterations of the pipeline, especially with regards to the ideal number of K-means clusters.

Source	Genus	Superspecies	Species	Group	Subspecies
Dowsett (1986)	-4	9	0	5	7
Bowie (2003)	-3	15	6	-3	3
This study	1	3	6	12	2

3.1 Introduction: Creating Consensus Trees

```
library(ape)
library(phytools)
```

```
## Loading required package: maps

##
## Attaching package: 'maps'

## The following object is masked from 'package:purrr':
##
##     map

##
## Attaching package: 'phytools'

## The following object is masked from 'package:dendextend':
##
##     untangle

## The following object is masked from 'package:vegan':
##
##     scores
```

```
library(ggtree)
```

```
## ggtree v3.0.4  For help: https://yulab-smu.top/treedata-book/
##
## If you use ggtree in published research, please cite the most appropriate paper(s):
##
## 1. Guangchuang Yu. Using ggtree to visualize data on tree-like structures. Current Protocols in Bioi
## 2. Guangchuang Yu, Tommy Tsan-Yuk Lam, Huachen Zhu, Yi Guan. Two methods for mapping and visualizing
## 3. Guangchuang Yu, David Smith, Huachen Zhu, Yi Guan, Tommy Tsan-Yuk Lam. ggtree: an R package for vi

##
## Attaching package: 'ggtree'

## The following object is masked from 'package:dendextend':
##
##     rotate

## The following object is masked from 'package:ape':
##
##     rotate

## The following object is masked from 'package:tidyr':
##
##     expand
```

We are going to create a consensus tree across the hierarchies using `ape`.

3.2 Cooper Trees

```
trees=list.files(filepath,pattern="*_Cooper.tre")
trees=paste0(filepath,trees)

tre1=read.tree(trees[1])
tre2=read.tree(trees[2])
tre3=read.tree(trees[3])
tre4=read.tree(trees[4])
tre5=read.tree(trees[5])

x.con=consensus(tre1,tre2,tre3,tre4,tre5,p=0.5)

write.tree(x.con,file=paste0(filepath,"Cooper-all-trees.consensus"))

x.con=fortify(x.con)

p1=ggtree(x.con)
p2=geom_tiplab(size=3)
p3=xlim(c(0,max(x.con$x)+2))
print(p1+p2+p3)
```

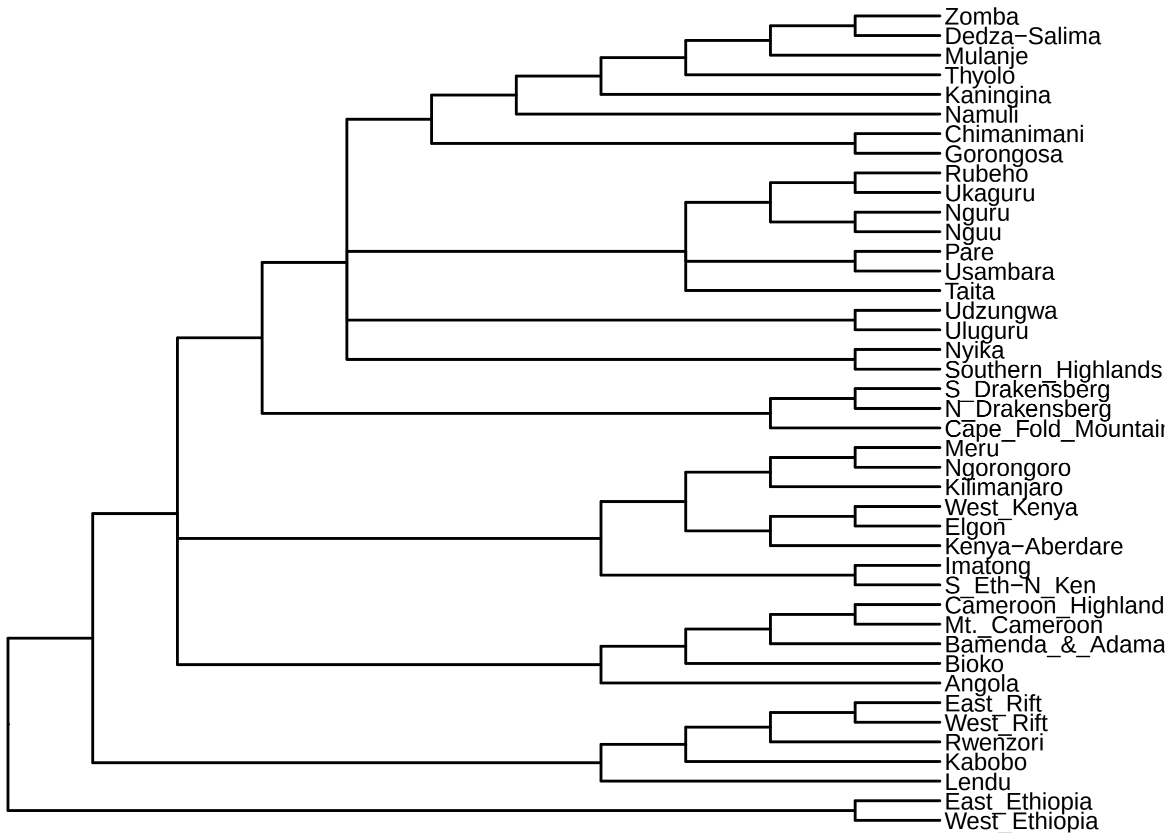

3.3 Bowie Trees

```
trees=list.files(filepath,pattern="*_Bowie.tre")
trees=paste0(filepath,trees)

tre1=read.tree(trees[1])
tre2=read.tree(trees[2])
tre3=read.tree(trees[3])
tre4=read.tree(trees[4])
tre5=read.tree(trees[5])

x.con=consensus(tre1,tre2,tre3,tre4,tre5,p=0.5)

write.tree(x.con,file=paste0(filepath,"Bowie-all-trees.consensus"))

x.con=fortify(x.con)

p1=ggtree(x.con)
p2=geom_tiplab(size=3)
p3=xlim(c(0,max(x.con$x)+2))
print(p1+p2+p3)
```

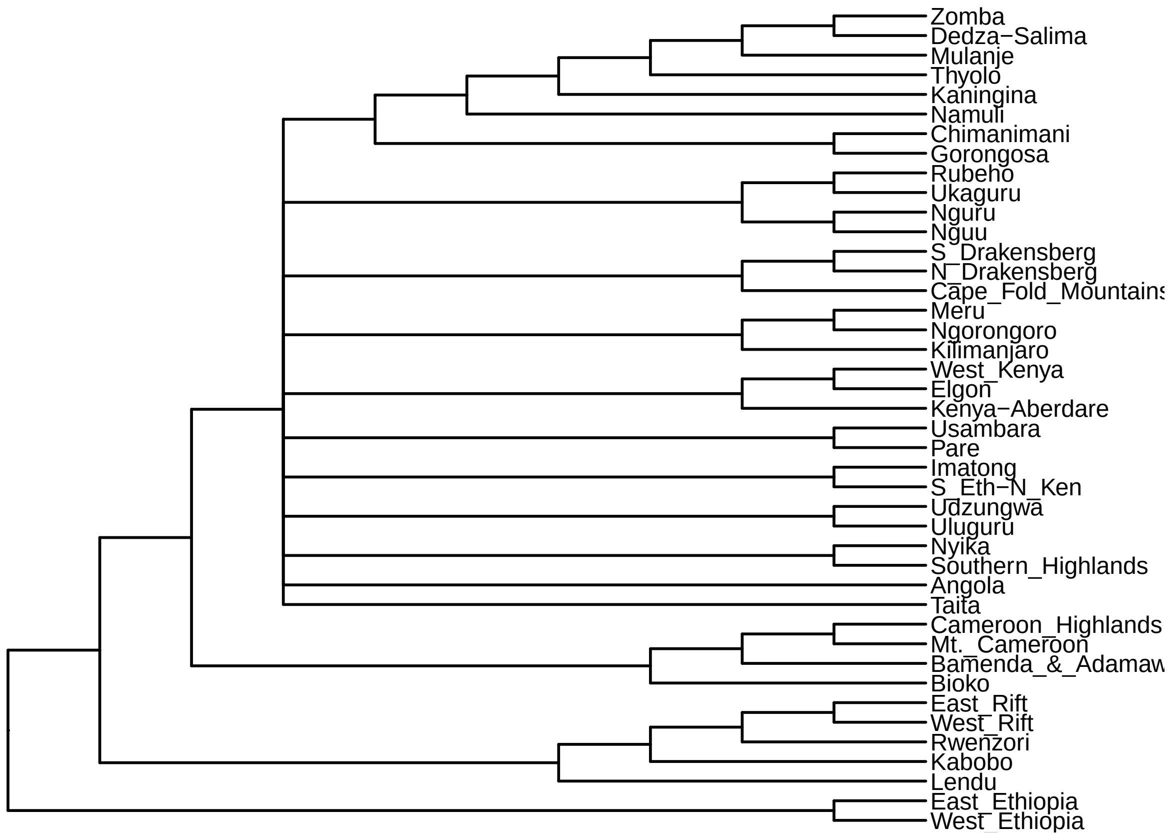

3.4 Dowsett Trees

```
trees=list.files(filepath,pattern="*_Dowsett.tre")
trees=paste0(filepath,trees)

tre1=read.tree(trees[1])
tre2=read.tree(trees[2])
tre3=read.tree(trees[3])
tre4=read.tree(trees[4])
tre5=read.tree(trees[5])

x.con=consensus(tre1,tre2,tre3,tre4,tre5,p=0.5)

write.tree(x.con,file=paste0(filepath,"Dowsett-all-trees.consensus"))

x.con=fortify(x.con)

p1=ggtree(x.con)
p2=geom_tiplab(size=3)
p3=xlim(c(0,max(x.con$x)+2))
print(p1+p2+p3)
```

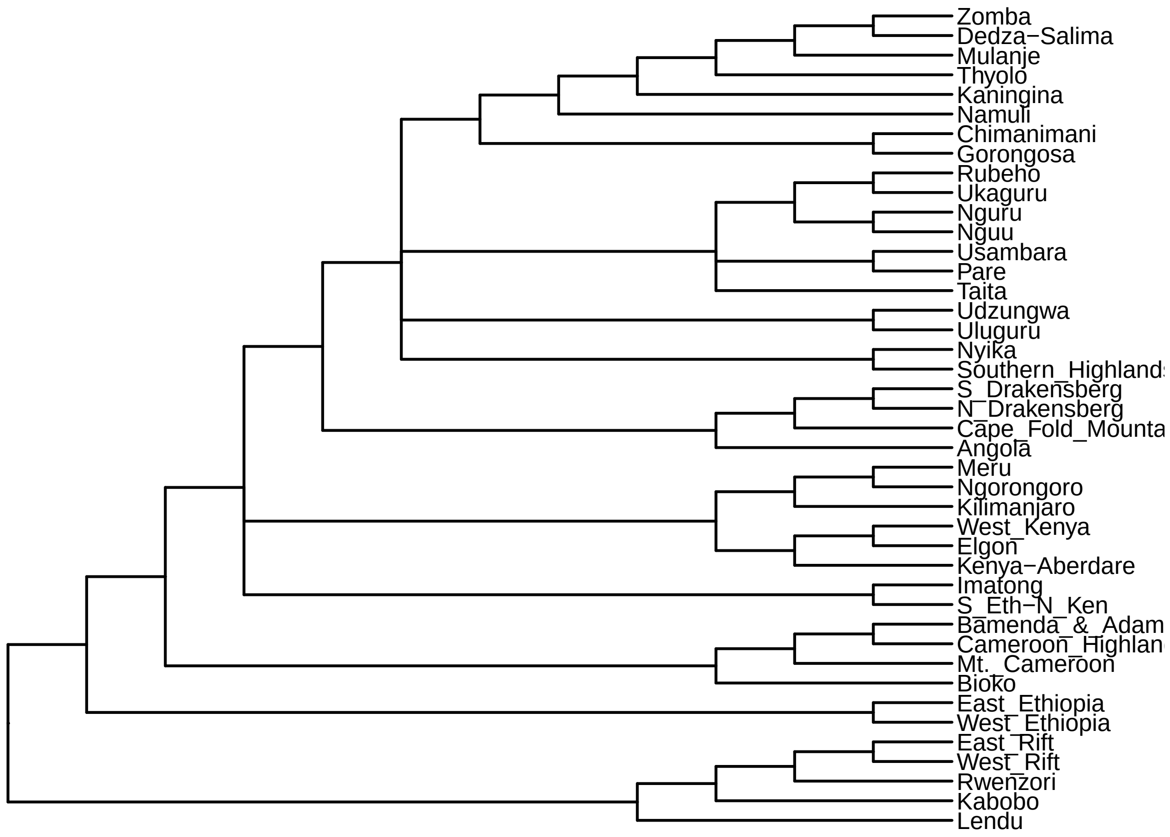

3.5 All Sources

```
coop=list.files(filepath,pattern="*_Cooper.tre")
bowi=list.files(filepath,pattern="*_Bowie.tre")
dows=list.files(filepath,pattern="*_Dowsett.tre")

trees=c(coop,bowi,dows)

trees=paste0(filepath,trees)

tre1=read.tree(trees[1])
tre2=read.tree(trees[2])
tre3=read.tree(trees[3])
tre4=read.tree(trees[4])
tre5=read.tree(trees[5])
tre6=read.tree(trees[6])
tre7=read.tree(trees[7])
tre8=read.tree(trees[8])
tre9=read.tree(trees[9])
tre10=read.tree(trees[10])
tre11=read.tree(trees[11])
tre12=read.tree(trees[12])
tre13=read.tree(trees[13])
tre14=read.tree(trees[14])
tre15=read.tree(trees[15])

x.con=consensus(tre1,tre2,tre3,
                tre4,tre5,tre6,
                tre7,tre8,tre9,
                tre10,tre11,tre12,
                tre13,tre14,tre15,
                p=0.5)

write.tree(x.con,file=paste0(filepath,"all_consensus.tre"))

x.con=fortify(x.con)

p1=ggtree(x.con)
p2=geom_tiplab(size=3)
p3=xlim(c(0,max(x.con$x)+2))
print(p1+p2+p3)
```

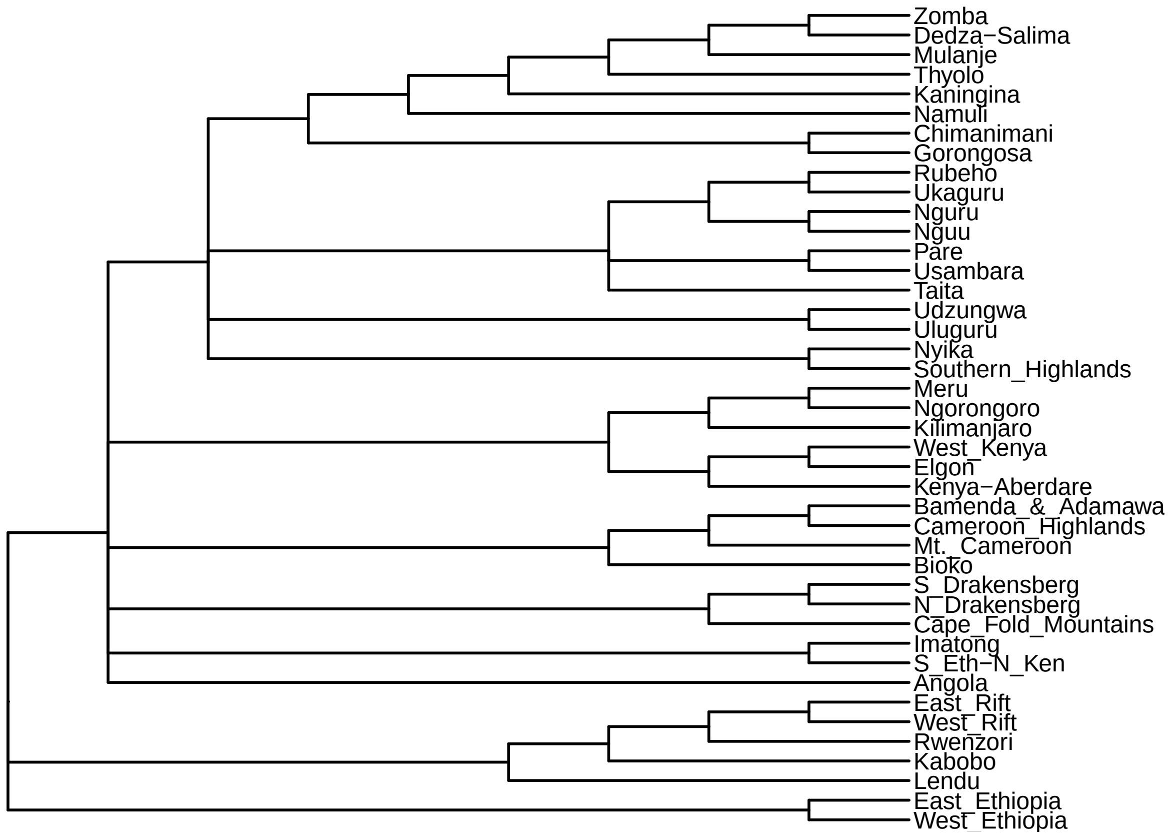

4.1 Introduction: Plotting Trees

This is intended to create nice, more readable trees for these analyses. I report some results from *k-means clustering* here, but **note** that all trees are derived from *hierarchical clustering* exercises. Groups for the trees are from the 'elbow' method and from viewing major clades *by the author*, both of which are non-exact heuristics that are subject to observer interpretation.

```
library(tidyverse)
library(phytools)
library(data.table)
```

```
##
## Attaching package: 'data.table'

## The following object is masked from 'package:dendextend':
##
##     set

## The following objects are masked from 'package:dplyr':
##
##     between, first, last

## The following object is masked from 'package:purrr':
##
##     transpose
```

```
# for first time install use:
```

```
# BiocManager::install("ggtree")

library(tidytree)
```

```
##
## Attaching package: 'tidytree'

## The following object is masked from 'package:stats':
##
##     filter
```

```
library(ggtree)
library(RColorBrewer) # palette pulled from website
```

Note on KMeans

The algorithms used for `kmeans` clustering uses ten iterations to determine cluster assignments based on a provided k value. As this is an algorithm that runs repeatedly, results are not always identical between runs. Therefore, results shown here for `kmeans` are not necessarily identical to files provided or assignments determined during other runs of the algorithm. This is another reason why `kmeans` was used in conjunction with `hclust`, and why general patterns and topologies (including patterns in what regions are uncertain) are so important.

4.2 Phylogenetic Trees

Now, for plotting phylogenetic trees. Every tree has to be custom made, as there is not an easy way to pipeline the formation of these figures. Some of this code is derived from this online resource. **Note** that the linked tutorial uses the function `fortify` to convert the `tree` objects to `data.frame` objects. However, *this changes the tree's topology*, and results in different mapping. The topology is maintained if you use the *ggtree* function `"fortify"` instead.

First, we must get the names of all species.

```
# files defined for trees
```

We can also set a five color, colorblind friendly color scheme:

```
x.colors=c('#1b9e77', # greenish
           '#000000', # black
           '#d95f02', # orangish
           '#7570b3') # purple

# group go up to 9; rep this so adjacent groups are different colors

x.colors=rep(x.colors,2)

# load coordinate data

meta.x=read.csv(paste0(filepath,"locality_metadata.csv"))
colnames(meta.x)[1]="Region"
```

4.2.1 Genus

```
csvs=list.files(filepath,pattern="*.csv")
csvs=paste0(filepath,csvs)
```

```r
z.csv=read.csv(csvs[csvs%like%"Genus"&csvs%like%"Cooper"])
y.csv=read.csv(csvs[csvs%like%"Genus"&csvs%like%"Bowie"])
x.csv=read.csv(csvs[csvs%like%"Genus"&csvs%like%"Dowsett"])

colnames(x.csv)=c("Region","Dowsett")
colnames(y.csv)=c("Region","Bowie")
colnames(z.csv)=c("Region","Cooper")

genus_clusters=x.csv%>%inner_join(y.csv,by='Region')%>%
  inner_join(z.csv,by='Region')%>%
  inner_join(meta.x,by='Region')

write.csv(genus_clusters,file=paste0(filepath,"genus_cluster.csv"),
          row.names = F,quote = F)
```

```r
# how are regions split up?
# comparison Bowie and Dowsett

y=table(genus_clusters[,2:3])
print(y)
```

```
##        Bowie
## Dowsett  1  2  3  4
##       1 21  1  0  0
##       2  0  2  4  0
##       3  1  8  0  0
##       4  0  0  0  5
```

```r
d.b.sub=genus_clusters%>%select(Region,Dowsett,Bowie)%>%unique()
d.b.sub[order(d.b.sub$Dowsett),]
```

```
##                 Region Dowsett Bowie
## 12         S Eth-N Ken       1     2
## 20               Taita       1     1
## 23                Nguu       1     1
## 24               Nguru       1     1
## 25             Ukaguru       1     1
## 26             Rubeho        1     1
## 27             Uluguru       1     1
## 28            Udzungwa       1     1
## 29  Southern Highlands       1     1
## 30               Nyika       1     1
## 31           Kaningina       1     1
## 32        Dedza-Salima       1     1
## 33               Zomba       1     1
## 34              Thyolo       1     1
## 35             Mulanje       1     1
## 36              Namuli       1     1
## 37           Gorongosa       1     1
## 38         Chimanimani       1     1
## 39       N Drakensberg       1     1
## 40       S Drakensberg       1     1
## 41 Cape Fold Mountains       1     1
## 42              Angola       1     1
```

```
## 6             West Rift       2     3
## 7              Rwenzori       2     3
## 8             East Rift       2     3
## 9                Kabobo       2     3
## 10        West Ethiopia       2     2
## 11        East Ethiopia       2     2
## 13              Imatong       3     2
## 14                Elgon       3     2
## 15           West Kenya       3     2
## 16       Kenya-Aberdare       3     2
## 17           Ngorongoro       3     2
## 18                 Meru       3     2
## 19          Kilimanjaro       3     2
## 21                 Pare       3     2
## 22             Usambara       3     1
## 1                 Bioko       4     4
## 2          Mt. Cameroon       4     4
## 3    Cameroon Highlands       4     4
## 4     Bamenda & Adamawa       4     4
## 5                 Lendu       4     4
```

Note that Bowie and Dowsett here place Lendu with the Cameroon Highlands, not the Lacustrine [Albertine] Rift!

```
d.b.sub=genus_clusters%>%select(Region,Dowsett,Cooper)%>%unique()
d.b.sub[order(d.b.sub$Dowsett),]
```

Cooper, meanwhile, places Lendu and Kabobo as their own separate outgroup for this iteration.

```
z=read.tree(files[files%like%"Genus"&files%like%"Cooper"])
y=read.tree(files[files%like%"Genus"&files%like%"Bowie"])
x=read.tree(files[files%like%"Genus"&files%like%"Dowsett"])

z$tip.label=gsub("_"," ",z$tip.label)
x$tip.label=gsub("_"," ",x$tip.label)
y$tip.label=gsub("_"," ",y$tip.label)

x="fortify"(x)
y="fortify"(y)
z="fortify"(z)

y$x=y$x+max(x$x)+1
z$x=z$x+max(y$x)+1

dd=bind_rows(x,y,z)%>%
  filter(!is.na(label))
```

```
p1=ggtree(x,layout='rectangular')# leftmost tree
p2=geom_tree(data=y) # middle tree
p3=geom_tree(data=z) # rightmost tree
p4=geom_tiplab(data=z,size=2)
p5=geom_line(aes(x,y,group=label),data=dd,alpha=.3,size=0.3)
p6=xlim(c(0,max(z$x)+5))
p7=ylim(c(-0.5,max(z$y)))

plot1=p1+p2+p3+p4+p5+p6+p7
```

```
## Scale for 'y' is already present. Adding another scale for 'y', which will
## replace the existing scale.
```

```
plot1
```

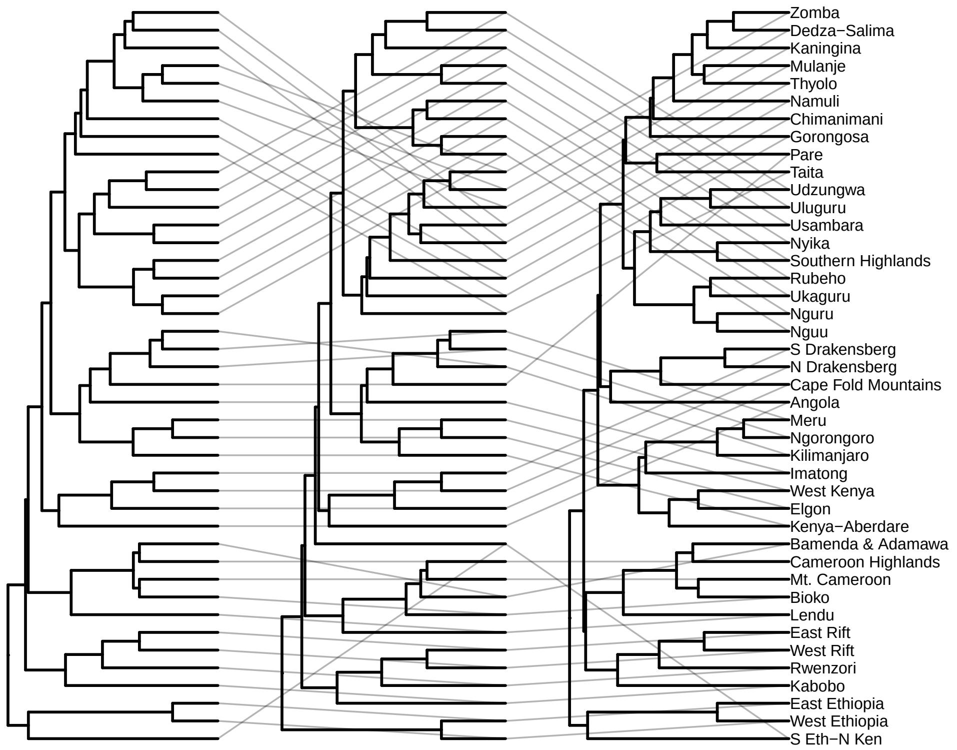


```
ggsave(paste0(filepath,"genus_compared.jpg"),
       plot = plot1,dpi = 400)
```

```
## Saving 6.5 x 4.5 in image
```

4.2.2 Superspecies

```
z.csv=read.csv(csvs[csvs%like%"Superspecies"&csvs%like%"Cooper"])
y.csv=read.csv(csvs[csvs%like%"Superspecies"&csvs%like%"Bowie"])
x.csv=read.csv(csvs[csvs%like%"Superspecies"&csvs%like%"Dowsett"])

colnames(x.csv)=c("Region","Dowsett")
colnames(y.csv)=c("Region","Bowie")
colnames(z.csv)=c("Region","Cooper")

subset_clusters=x.csv%>%inner_join(y.csv,by='Region')%>%
  inner_join(z.csv,by='Region')%>%
  inner_join(meta.x,by='Region')

write.csv(subset_clusters,file=paste0(filepath,"superspecies_cluster.csv"),
          row.names = F,quote = F)
```

```r
d.b.sub=subset_clusters%>%select(Region,Dowsett,Bowie)%>%unique()
d.b.sub[order(d.b.sub$Dowsett),]
```

```
##                 Region Dowsett Bowie
## 31           Kaningina       1     1
## 32        Dedza-Salima       1     1
## 33               Zomba       1     1
## 34              Thyolo       1     1
## 35             Mulanje       1     1
## 36              Namuli       1    12
## 5                Lendu       2    10
## 9               Kabobo       2    16
## 14               Elgon       3     2
## 15          West Kenya       3     2
## 16      Kenya-Aberdare       3     2
## 1                Bioko       4     4
## 2         Mt. Cameroon       4     4
## 3   Cameroon Highlands       4     4
## 4    Bamenda & Adamawa       4     4
## 12          S Eth-N Ken       5     5
## 13             Imatong       5     3
## 39       N Drakensberg       6     6
## 40       S Drakensberg       6     6
## 41 Cape Fold Mountains       6     6
## 23                Nguu       7     7
## 24               Nguru       7     7
## 25             Ukaguru       7     8
## 26              Rubeho       7     8
## 20               Taita       8    13
## 21                Pare       8    13
## 22            Usambara       8     9
## 27             Uluguru       9    14
## 28            Udzungwa       9    14
## 29  Southern Highlands       9    15
## 30               Nyika       9    15
## 6            West Rift      10    18
## 7             Rwenzori      10    19
## 8            East Rift      10    18
## 17          Ngorongoro      11    11
## 18                Meru      11    11
## 19         Kilimanjaro      11    11
## 37           Gorongosa      12    12
## 38         Chimanimani      12    12
## 42              Angola      12    17
## 10       West Ethiopia      13    20
## 11       East Ethiopia      13    20
```

```r
d.b.sub=subset_clusters%>%select(Region,Dowsett,Cooper)%>%unique()
d.b.sub[order(d.b.sub$Cooper),]
```

```
##                 Region Dowsett Cooper
## 31           Kaningina       1      1
## 32        Dedza-Salima       1      1
## 33               Zomba       1      1
```

```
## 34             Thyolo       1       1
## 35            Mulanje       1       1
## 36             Namuli       1       1
## 10       West Ethiopia      13       2
## 11       East Ethiopia      13       2
## 13            Imatong       5       3
## 14              Elgon       3       3
## 15         West Kenya       3       3
## 16     Kenya-Aberdare       3       3
## 17         Ngorongoro      11       3
## 18               Meru      11       3
## 19        Kilimanjaro      11       3
## 1               Bioko       4       4
## 2         Mt. Cameroon       4       4
## 3   Cameroon Highlands       4       4
## 4    Bamenda & Adamawa       4       4
## 37          Gorongosa      12       5
## 38        Chimanimani      12       5
## 42             Angola      12       5
## 39       N Drakensberg       6       6
## 40       S Drakensberg       6       6
## 41 Cape Fold Mountains       6       6
## 23               Nguu       7       7
## 24              Nguru       7       7
## 25            Ukaguru       7       7
## 26             Rubeho       7       7
## 12         S Eth-N Ken       5       8
## 20              Taita       8       8
## 21               Pare       8       8
## 22           Usambara       8       9
## 27            Uluguru       9       9
## 28           Udzungwa       9       9
## 29  Southern Highlands       9       9
## 30              Nyika       9       9
## 5               Lendu       2      10
## 6           West Rift      10      10
## 7            Rwenzori      10      10
## 8           East Rift      10      10
## 9              Kabobo       2      10
```

```
z=read.tree(files[files%like%"Superspecies"&files%like%"Cooper"])
y=read.tree(files[files%like%"Superspecies"&files%like%"Bowie"])
x=read.tree(files[files%like%"Superspecies"&files%like%"Dowsett"])

z$tip.label=gsub("_"," ",z$tip.label)
x$tip.label=gsub("_"," ",x$tip.label)
y$tip.label=gsub("_"," ",y$tip.label)

x=fortify(x)
y=fortify(y)
z=fortify(z)

y$x=y$x+max(x$x)+1
z$x=z$x+max(y$x)+1
```

```r
dd=bind_rows(x,y,z)%>%
  filter(!is.na(label))

p1=ggtree(x,layout='rectangular')
p2=geom_tree(data=y)
p3=geom_tree(data=z)
p4=geom_tiplab(data=z,size=2)
p5=geom_line(aes(x,y,group=label),data=dd,alpha=.3,size=0.3)
p6=xlim(c(0,max(z$x)+5))
p7=ylim(c(-0.5,max(z$y)))

plot1=p1+p2+p3+p4+p5+p6+p7
```

```
## Scale for 'y' is already present. Adding another scale for 'y', which will
## replace the existing scale.
```

```r
plot1
```

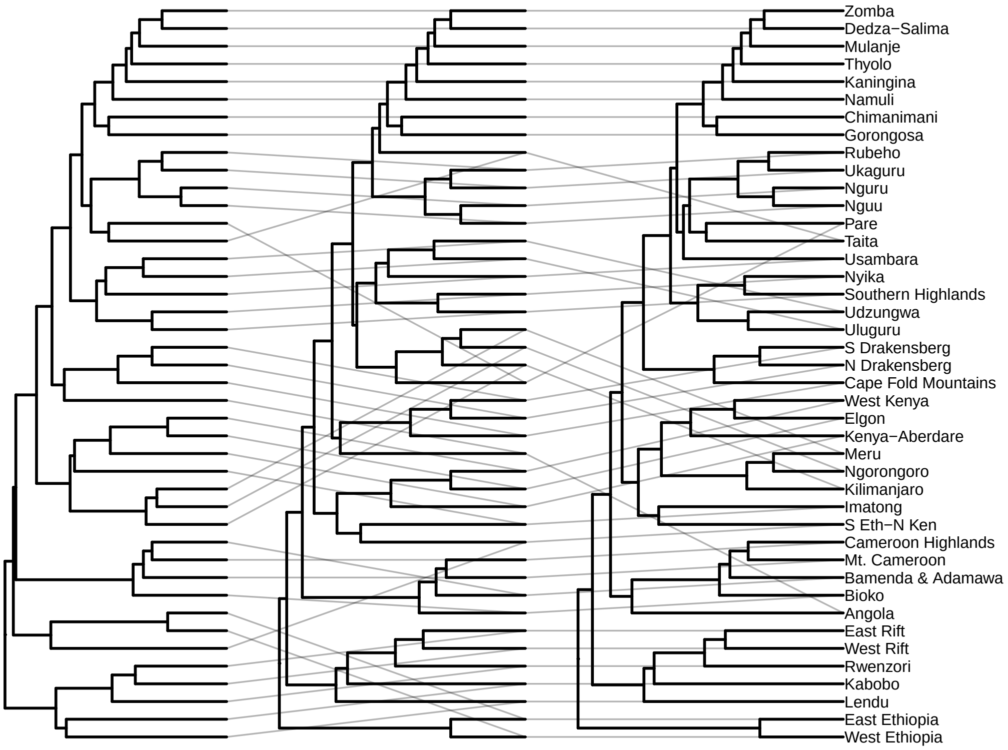


```r
ggsave(paste0(filepath,"superspecies_compared.jpg"),
       plot = plot1,dpi = 400)
```

```
## Saving 6.5 x 4.5 in image
```

4.2.3 Species

```
z.csv=read.csv(csvs[csvs%like%"Species"&csvs%like%"Cooper"])
y.csv=read.csv(csvs[csvs%like%"Species"&csvs%like%"Bowie"])
x.csv=read.csv(csvs[csvs%like%"Species"&csvs%like%"Dowsett"])

colnames(x.csv)=c("Region","Dowsett")
colnames(y.csv)=c("Region","Bowie")
colnames(z.csv)=c("Region","Cooper")

subset_clusters=x.csv%>%inner_join(y.csv,by='Region')%>%
  inner_join(z.csv,by='Region')%>%
  inner_join(meta.x,by='Region')

write.csv(subset_clusters,file=paste0(filepath,"Species_cluster.csv"),
          row.names = F,quote = F)
```

```
##        Bowie
## Dowsett 1 2 3 4 5 6 7 8 9 10
##       1 6 0 0 0 0 0 8 3 0  0
##       2 0 0 0 0 0 0 0 0 0  5
##       3 0 4 1 0 2 0 0 0 3  0
##       4 0 0 0 4 0 0 0 0 0  0
##       5 1 0 1 0 0 4 0 0 0  0
```

```
##      Cooper
## Bowie 1 2 3 4 5 6 7 8 9 10
##    1  6 0 0 0 1 0 0 0 0  0
##    2  0 0 4 0 0 0 0 0 0  0
##    3  0 0 0 0 1 0 0 1 0  0
##    4  0 0 0 4 0 0 0 0 0  0
##    5  0 2 0 0 0 0 0 0 0  0
##    6  0 0 0 0 1 3 0 0 0  0
##    7  1 0 0 0 0 0 4 0 3  0
##    8  0 0 0 0 1 0 0 0 2  0
##    9  0 0 0 0 0 0 0 0 3  0
##    10 0 0 0 0 0 0 0 0 0  5
```

```
d.b.sub=subset_clusters%>%select(Region,Dowsett,Bowie)%>%unique()
d.b.sub[order(d.b.sub$Dowsett),]
```

```
##                  Region Dowsett Bowie
## 20                Taita       1     8
## 21                 Pare       1     8
## 22             Usambara       1     8
## 23                 Nguu       1     7
## 24                Nguru       1     7
## 25              Ukaguru       1     7
## 26              Rubeho       1     7
## 27              Uluguru       1     7
## 28             Udzungwa       1     7
## 29  Southern Highlands       1     7
## 30                Nyika       1     7
## 31            Kaningina       1     1
## 32         Dedza-Salima       1     1
## 33                Zomba       1     1
## 34               Thyolo       1     1
```

```
## 35             Mulanje       1     1
## 36              Namuli       1     1
## 5                Lendu       2    10
## 6            West Rift       2    10
## 7             Rwenzori       2    10
## 8            East Rift       2    10
## 9               Kabobo       2    10
## 10       West Ethiopia       3     5
## 11       East Ethiopia       3     5
## 12         S Eth-N Ken       3     3
## 13             Imatong       3     2
## 14               Elgon       3     2
## 15          West Kenya       3     2
## 16     Kenya-Aberdare       3     2
## 17          Ngorongoro       3     9
## 18                Meru       3     9
## 19         Kilimanjaro       3     9
## 1                Bioko       4     4
## 2          Mt. Cameroon       4     4
## 3    Cameroon Highlands       4     4
## 4     Bamenda & Adamawa       4     4
## 37           Gorongosa       5     1
## 38          Chimanimani       5     6
## 39        N Drakensberg       5     6
## 40        S Drakensberg       5     6
## 41 Cape Fold Mountains       5     6
## 42              Angola       5     3
```

Now, to plot the tree to view topology and to determine best format:

```
z=read.tree(files[files%like%"Species"&files%like%"Cooper"])
y=read.tree(files[files%like%"Species"&files%like%"Bowie"])
x=read.tree(files[files%like%"Species"&files%like%"Dowsett"])

z$tip.label=gsub("_"," ",z$tip.label)
x$tip.label=gsub("_"," ",x$tip.label)
y$tip.label=gsub("_"," ",y$tip.label)

x=fortify(x)
y=fortify(y)
z=fortify(z)

y$x=y$x+max(x$x)+1
z$x=z$x+max(y$x)+1

dd=bind_rows(x,y,z)%>%
  filter(!is.na(label))

p1=ggtree(x,layout='rectangular')
p2=geom_tree(data=y)
p3=geom_tree(data=z)
p4=geom_tiplab(data=z,size=2)
p5=geom_line(aes(x,y,group=label),data=dd,alpha=.3,size=0.3)
p6=xlim(c(0,max(z$x)+5))
p7=ylim(c(-0.5,max(z$y)))
```

```
plot1=p1+p2+p3+p4+p5+p6+p7
```

```
## Scale for 'y' is already present. Adding another scale for 'y', which will
## replace the existing scale.
```

```
plot1
```

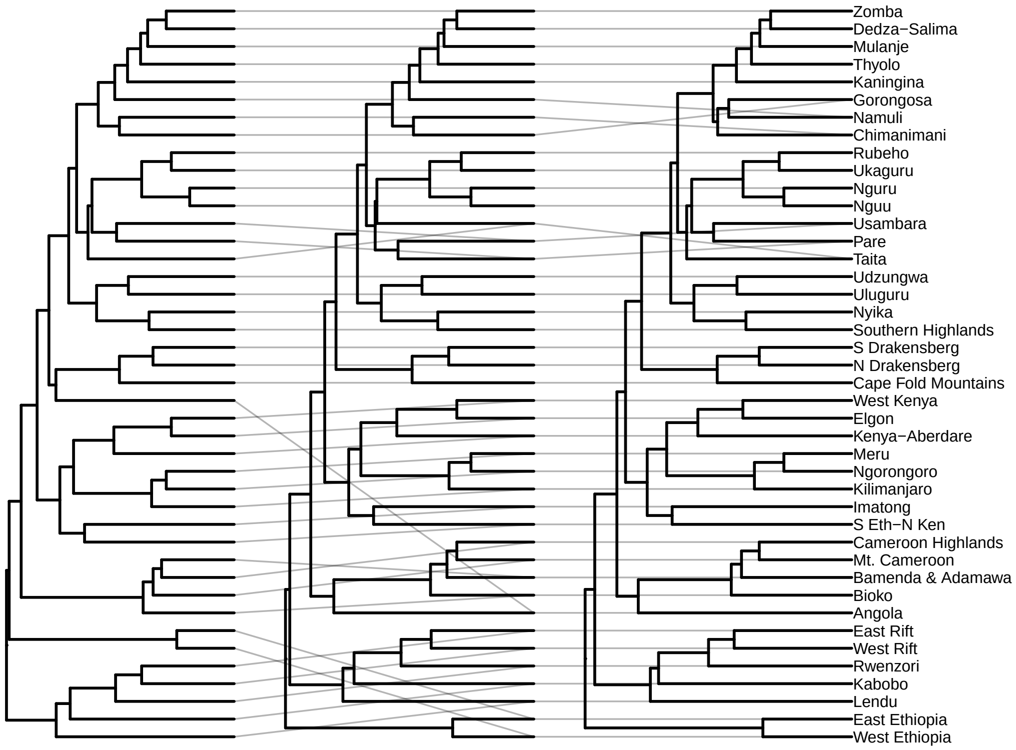


```
ggsave(paste0(filepath,"species_compared.jpg"),
       plot = plot1,dpi = 400)
```

```
## Saving 6.5 x 4.5 in image
```

4.2.4 Group

```
z.csv=read.csv(csvs[csvs%like%"Group"&csvs%like%"Cooper"])
y.csv=read.csv(csvs[csvs%like%"Group"&csvs%like%"Bowie"])
x.csv=read.csv(csvs[csvs%like%"Group"&csvs%like%"Dowsett"])

colnames(x.csv)=c("Region","Dowsett")
colnames(y.csv)=c("Region","Bowie")
colnames(z.csv)=c("Region","Cooper")

subset_clusters=x.csv%>%inner_join(y.csv,by='Region')%>%
  inner_join(z.csv,by='Region')%>%
  inner_join(meta.x,by='Region')
```

```r
write.csv(subset_clusters,file=paste0(filepath,"Group_cluster.csv"),
          row.names = F,quote = F)
```

```
##        Bowie
## Dowsett  1 2 3
##      1   7 0 0
##      2   1 2 1
##      3   0 3 0
##      4   4 0 0
##      5   0 2 0
##      6   3 0 0
##      7   7 0 0
##      8   2 3 0
##      9   3 0 0
##      10  0 0 4
```

```
##      Cooper
## Bowie  1 2 3 4 5 6 7 8 9 10 11 12 13 14 15 16
##     1  3 0 0 4 2 3 4 0 3  0  0  3  1  2  2  0
##     2  0 3 2 0 0 0 0 2 0  0  3  0  0  0  0  0
##     3  0 0 0 0 0 0 0 0 0  2  0  0  0  0  0  3
```

```r
d.b.sub=subset_clusters%>%select(Region,Dowsett,Bowie)%>%unique()
d.b.sub[order(d.b.sub$Dowsett),]
```

```
##                 Region Dowsett Bowie
## 32        Dedza-Salima       1     1
## 33               Zomba       1     1
## 34              Thyolo       1     1
## 35             Mulanje       1     1
## 36              Namuli       1     1
## 37           Gorongosa       1     1
## 38          Chimanimani       1     1
## 5                Lendu       2     3
## 12          S Eth-N Ken       2     2
## 13             Imatong       2     2
## 42              Angola       2     1
## 14               Elgon       3     2
## 15          West Kenya       3     2
## 16      Kenya-Aberdare       3     2
## 1                Bioko       4     1
## 2          Mt. Cameroon       4     1
## 3    Cameroon Highlands       4     1
## 4     Bamenda & Adamawa       4     1
## 10        West Ethiopia       5     2
## 11        East Ethiopia       5     2
## 39        N Drakensberg       6     1
## 40        S Drakensberg       6     1
## 41  Cape Fold Mountains       6     1
## 22             Usambara       7     1
## 23                 Nguu       7     1
## 24                Nguru       7     1
## 25              Ukaguru       7     1
## 26               Rubeho       7     1
## 27              Uluguru       7     1
```

```
## 28            Udzungwa       7      1
## 17          Ngorongoro       8      2
## 18                Meru       8      2
## 19         Kilimanjaro       8      2
## 20               Taita       8      1
## 21                Pare       8      1
## 29  Southern Highlands       9      1
## 30               Nyika       9      1
## 31           Kaningina       9      1
## 6            West Rift      10      3
## 7             Rwenzori      10      3
## 8            East Rift      10      3
## 9               Kabobo      10      3
```

Now, to plot the tree to view topology and to determine best format:

```
z=read.tree(files[files%like%"Group"&files%like%"Cooper"])
y=read.tree(files[files%like%"Group"&files%like%"Bowie"])
x=read.tree(files[files%like%"Group"&files%like%"Dowsett"])

z$tip.label=gsub("_"," ",z$tip.label)
x$tip.label=gsub("_"," ",x$tip.label)
y$tip.label=gsub("_"," ",y$tip.label)

x=fortify(x)
y=fortify(y)
z=fortify(z)

y$x=y$x+max(x$x)+1
z$x=z$x+max(y$x)+1

dd=bind_rows(x,y,z)%>%
  filter(!is.na(label))

p1=ggtree(x,layout='rectangular')
p2=geom_tree(data=y)
p3=geom_tree(data=z)
p4=geom_tiplab(data=z,size=2)
p5=geom_line(aes(x,y,group=label),data=dd,alpha=.3,size=0.3)
p6=xlim(c(0,max(z$x)+5))
p7=ylim(c(-0.5,max(z$y)))

plot1=p1+p2+p3+p4+p5+p6+p7
```

```
## Scale for 'y' is already present. Adding another scale for 'y', which will
## replace the existing scale.
```

```
plot1
```

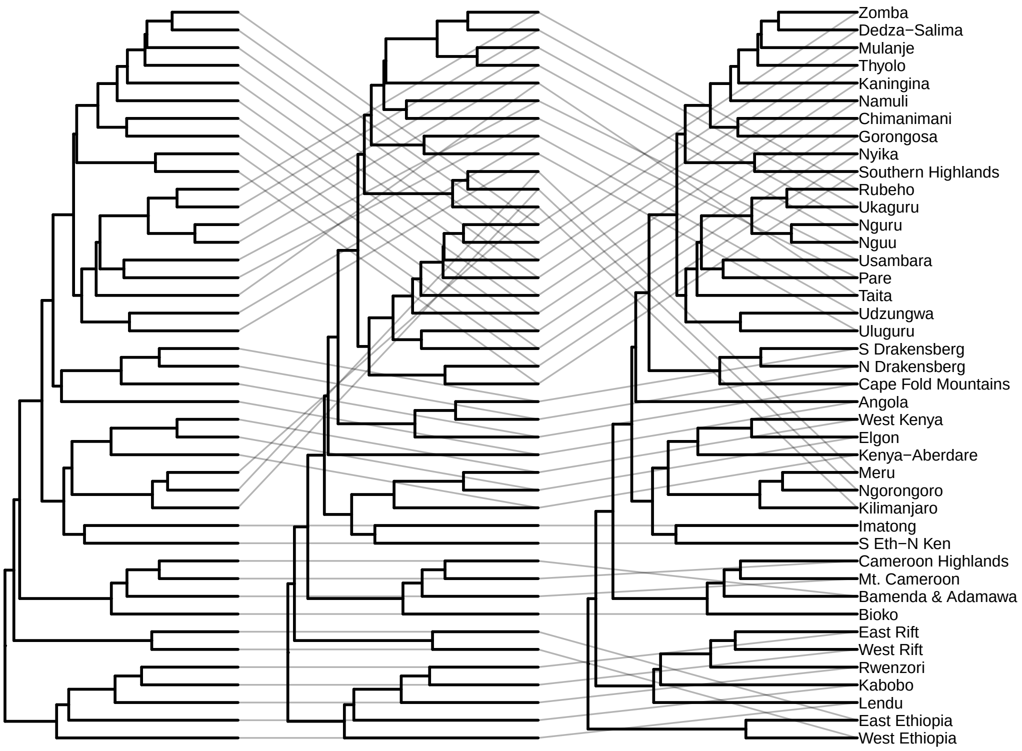


```
ggsave(paste0(filepath,"Group_compared.jpg"),
       plot = plot1,dpi = 400)
```

```
## Saving 6.5 x 4.5 in image
```

4.2.5 Subspecies

```
z.csv=read.csv(csvs[csvs%like%"Subspecies"&csvs%like%"Cooper"])
y.csv=read.csv(csvs[csvs%like%"Subspecies"&csvs%like%"Bowie"])
x.csv=read.csv(csvs[csvs%like%"Subspecies"&csvs%like%"Dowsett"])

colnames(x.csv)=c("Region","Dowsett")
colnames(y.csv)=c("Region","Bowie")
colnames(z.csv)=c("Region","Cooper")

subset_clusters=x.csv%>%inner_join(y.csv,by='Region')%>%
  inner_join(z.csv,by='Region')%>%
  inner_join(meta.x,by='Region')

write.csv(subset_clusters,file=paste0(filepath,"Subspecies_cluster.csv"),
          row.names = F,quote = F)
```

```
##         Bowie
## Dowsett 1 2 3 4 5 6 7 8 9 10 11
##       1  1 0 0 0 0 0 0 0 2  0  0
##       2  0 3 0 0 0 0 0 0 0  0  0
```

```
##      3  0 0 1 0 0 0 0 0 0  0  0
##      4  0 0 0 4 0 0 0 0 0  0  0
##      5  0 0 0 0 2 0 0 0 0  0  0
##      6  0 0 0 0 0 7 0 0 0  0  0
##      7  0 0 0 0 0 0 0 3 0  0  0
##      8  0 0 0 0 0 0 4 0 0  0  0
##      9  0 0 0 0 0 0 0 0 2  0  0
##      10 0 0 0 0 0 0 0 0 0  5  0
##      11 0 0 0 0 0 0 0 0 0  0  3
##      12 5 0 0 0 0 0 0 0 0  0  0
```

```
##      Cooper
## Bowie 1 2 3 4 5 6 7 8
##    1  6 0 0 0 0 0 0 0
##    2  0 2 0 0 0 0 0 1
##    3  0 1 0 0 0 0 0 0
##    4  0 0 0 4 0 0 0 0
##    5  0 0 0 0 2 0 0 0
##    6  2 0 0 0 0 3 0 2
##    7  0 0 0 0 0 0 4 0
##    8  0 0 0 0 0 0 3 0
##    9  2 0 0 0 0 0 2 0
##    10 0 0 5 0 0 0 0 0
##    11 0 3 0 0 0 0 0 0
```

```
d.b.sub=subset_clusters%>%select(Region,Dowsett,Bowie)%>%unique()
d.b.sub[order(d.b.sub$Dowsett),]
```

```
##                  Region Dowsett Bowie
## 29  Southern Highlands       1     9
## 30               Nyika       1     9
## 31           Kaningina       1     1
## 13             Imatong       2     2
## 14               Elgon       2     2
## 15          West Kenya       2     2
## 16      Kenya-Aberdare       3     3
## 1                Bioko       4     4
## 2          Mt. Cameroon       4     4
## 3   Cameroon Highlands       4     4
## 4    Bamenda & Adamawa       4     4
## 10        West Ethiopia       5     5
## 11        East Ethiopia       5     5
## 12          S Eth-N Ken       6     6
## 37           Gorongosa       6     6
## 38          Chimanimani       6     6
## 39        N Drakensberg       6     6
## 40        S Drakensberg       6     6
## 41 Cape Fold Mountains       6     6
## 42              Angola       6     6
## 20               Taita       7     8
## 21                Pare       7     8
## 22            Usambara       7     8
## 23                Nguu       8     7
## 24               Nguru       8     7
## 25             Ukaguru       8     7
```

```
## 26             Rubeho       8     7
## 27            Uluguru       9     9
## 28           Udzungwa       9     9
## 5               Lendu      10    10
## 6           West Rift      10    10
## 7            Rwenzori      10    10
## 8           East Rift      10    10
## 9              Kabobo      10    10
## 17         Ngorongoro      11    11
## 18               Meru      11    11
## 19        Kilimanjaro      11    11
## 32       Dedza-Salima      12     1
## 33              Zomba      12     1
## 34             Thyolo      12     1
## 35            Mulanje      12     1
## 36             Namuli      12     1
```

```
d.b.sub=subset_clusters%>%select(Region,Dowsett,Cooper)%>%unique()
d.b.sub[order(d.b.sub$Dowsett),]
```

```
##                 Region Dowsett Cooper
## 29  Southern Highlands       1      1
## 30               Nyika       1      1
## 31           Kaningina       1      1
## 13             Imatong       2      8
## 14               Elgon       2      2
## 15          West Kenya       2      2
## 16      Kenya-Aberdare       3      2
## 1                Bioko       4      4
## 2          Mt. Cameroon       4      4
## 3   Cameroon Highlands       4      4
## 4     Bamenda & Adamawa       4      4
## 10        West Ethiopia       5      5
## 11        East Ethiopia       5      5
## 12          S Eth-N Ken       6      8
## 37           Gorongosa       6      1
## 38          Chimanimani       6      1
## 39         N Drakensberg       6      6
## 40         S Drakensberg       6      6
## 41 Cape Fold Mountains       6      6
## 42              Angola       6      8
## 20               Taita       7      7
## 21                Pare       7      7
## 22            Usambara       7      7
## 23                Nguu       8      7
## 24               Nguru       8      7
## 25             Ukaguru       8      7
## 26              Rubeho       8      7
## 27             Uluguru       9      7
## 28            Udzungwa       9      7
## 5                Lendu      10      3
## 6            West Rift      10      3
## 7             Rwenzori      10      3
## 8            East Rift      10      3
## 9               Kabobo      10      3
```

```
## 17          Ngorongoro      11      2
## 18                Meru      11      2
## 19         Kilimanjaro      11      2
## 32        Dedza-Salima      12      1
## 33               Zomba      12      1
## 34              Thyolo      12      1
## 35             Mulanje      12      1
## 36              Namuli      12      1
```

Now, to plot the tree to view topology and to determine best format:

```
z=read.tree(files[files%like%"Subspecies"&files%like%"Cooper"])
y=read.tree(files[files%like%"Subspecies"&files%like%"Bowie"])
x=read.tree(files[files%like%"Subspecies"&files%like%"Dowsett"])

z$tip.label=gsub("_"," ",z$tip.label)
x$tip.label=gsub("_"," ",x$tip.label)
y$tip.label=gsub("_"," ",y$tip.label)

x=fortify(x)
y=fortify(y)
z=fortify(z)

y$x=y$x+max(x$x)+1
z$x=z$x+max(y$x)+1

dd=bind_rows(x,y,z)%>%
  filter(!is.na(label))

p1=ggtree(x,layout='rectangular')
p2=geom_tree(data=y)
p3=geom_tree(data=z)
p4=geom_tiplab(data=z,size=2)
p5=geom_line(aes(x,y,group=label),data=dd,alpha=.3,size=0.3)
p6=xlim(c(0,max(z$x)+5))
p7=ylim(c(-0.5,max(z$y)))

plot1=p1+p2+p3+p4+p5+p6+p7
```

```
## Scale for 'y' is already present. Adding another scale for 'y', which will
## replace the existing scale.
```

```
plot1
```

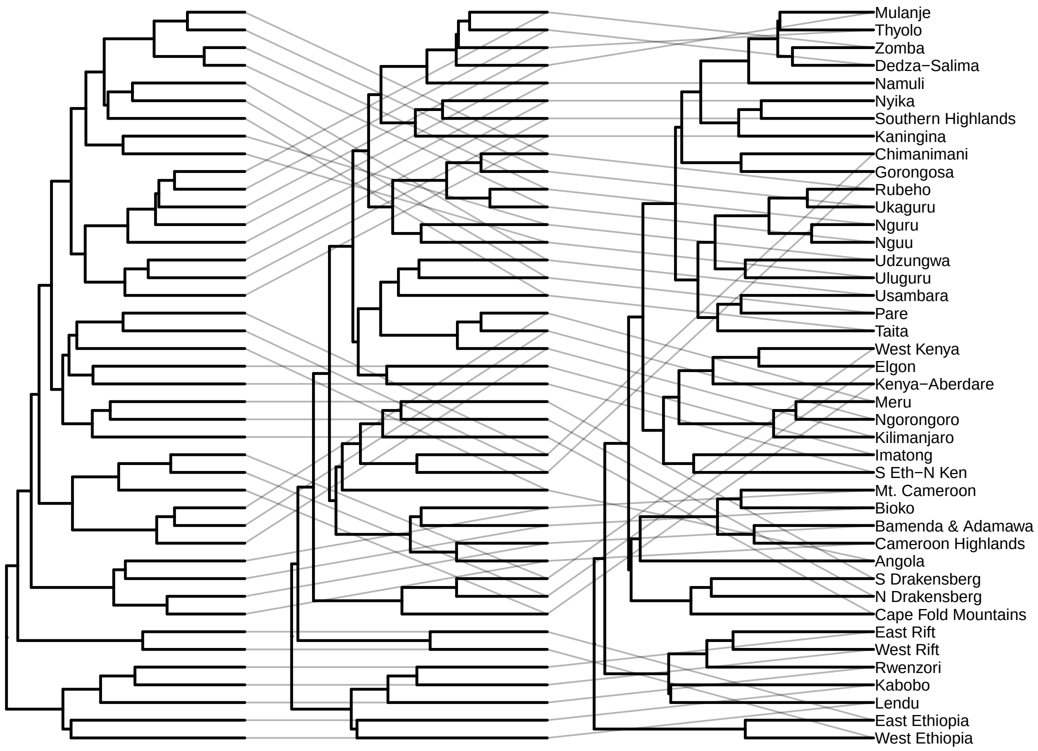


```
ggsave(paste0(filepath,"Subspecies_compared.jpg"),
       plot = plot1,dpi = 400)
```

```
## Saving 6.5 x 4.5 in image
```

4.3 Overall Consensus Tree

```
# read tree file
c.trees=list.files(filepath,pattern="*.consensus")

x=read.tree(paste0(filepath,"all_consensus.tre"))
x$tip.label=gsub("_"," ",x$tip.label)
x2=fortify(x)
```

Now, to plot the tree to view topology and to determine best format:

```
p2=ggtree(x2,layout = "rectangular")+
  geom_tiplab(size=2)+
  scale_color_manual(values="black")+
  expand_limits(x=c(0,10))

print(p2)
```

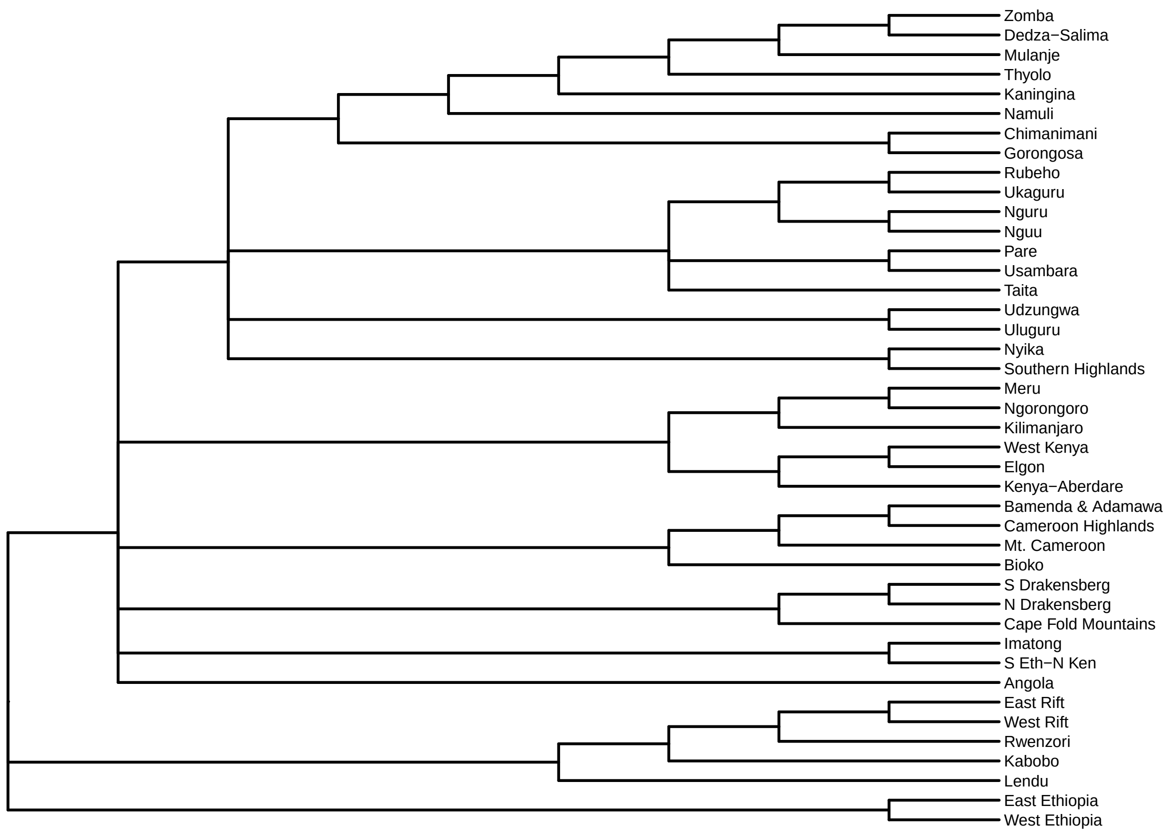

For all authors, the topology is largely polytomies.

I am now looking at the consensus for Cooper:

```
x=read.tree(paste0(filepath,c.trees[which(c.trees%like%'Cooper')]))
x$tip.label=gsub("_"," ",x$tip.label)
x2=fortify(x)

p2=ggtree(x2,layout = "rectangular")+
  geom_tiplab(size=2)+
  scale_color_manual(values="black")+
  expand_limits(x=c(0,10))

print(p2)
```

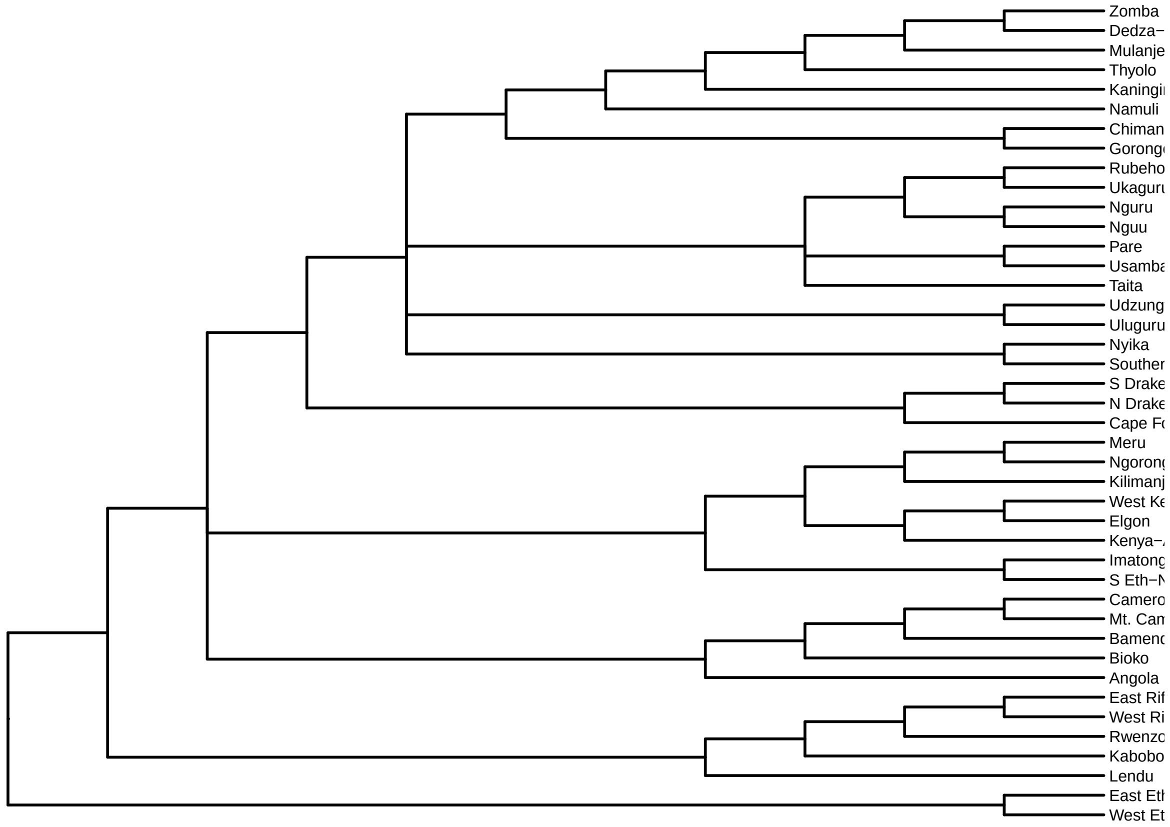

Within this tree, multiple regions are supported by the multiple taxonomic levels. These regions are user-defined based on previous iterations and the topology of the dendrogram.

3.1 Clustering for 2021 list

We find good support for the Ethiopian Highlands and the Lacustrine Rift as being distinct. However, we have a double polytomy, with a three way polytomy of:

1. Cameroonian Highlands + Angola
2. Kenya-Tanzania Highlands
3. All remaining groups excepting the Lacustrine Rift and the Ethiopian Highlands

And within the all remaining groups subset, the Southern Great Escarpment is the outgroup to all others, which constitute a polytomy of the **Expanded Eastern Arc**:

1. Northern Eastern Arc
2. Central Eastern Arc (Udzungwa & Uluguru)
3. Southern Highlands & Nyika
4. Malawi-Mozambique Highlands (central Malawi to Chimanimani, Zimbabwe/Mozambique)

3.2 Data with satellite regions, for comparison

The following is the clustering as determined with satellite regions included for the 2021 list. *Note* this also includes a few other small edits (i.e., fixed distributions etc., especially with regards to the Southern Highlands, Tanzania).

1. Ethiopian Highlands
2. Lacustrine Rift (including Lendu, Kabobo)

3. Kenya-Tanzania Highlands

- Two subregions of Kenya-Uganda and N Tanzania

  Here is the first polytomy; denoted by *A* after number.

4A. S Great Escarpment 5A. Cameroon Highlands 6A. Satellite Regions (including Angola) 7A. This branch leads to the second polytomy.

> Here is the second polytomy; denoted by *B* after number. It should be noted some of these divisions are less extreme that divisions found within the satellite regions. This area can also be considered as the "expanded" Eastern Arc.

8B. Taita Hills 9B. C Eastern Arc - Uluguru, Udzungwa, Nyika, Mahenge, Iringa 10B. Malawi-Mozambique - closely allied outliers of Chimanimani/Gorongosa - S Malawian and Mozambican Highlands 11B. Other E Arc, with two subdivisions: - Pare & Usambara Mountains - Nguu, Nguru, Malundwe, Ukaguru, Rubeho

4.4 Cooper Consensus Plot

```
# read tree file
z=x2

p2=ggtree(z,layout="rectangular")+
  geom_tiplab(size=2)+
  geom_text(aes(label=node),hjust=-.3,size=4)+
  #geom_text(aes(label=node),hjust=-.3)
  xlim(0,max(z$x)+2)#+
  #geom_nodepoint(color="black",
  #               size=(x2@phylo$node.label/25),
  #               shape="diamond")

print(p2)
```

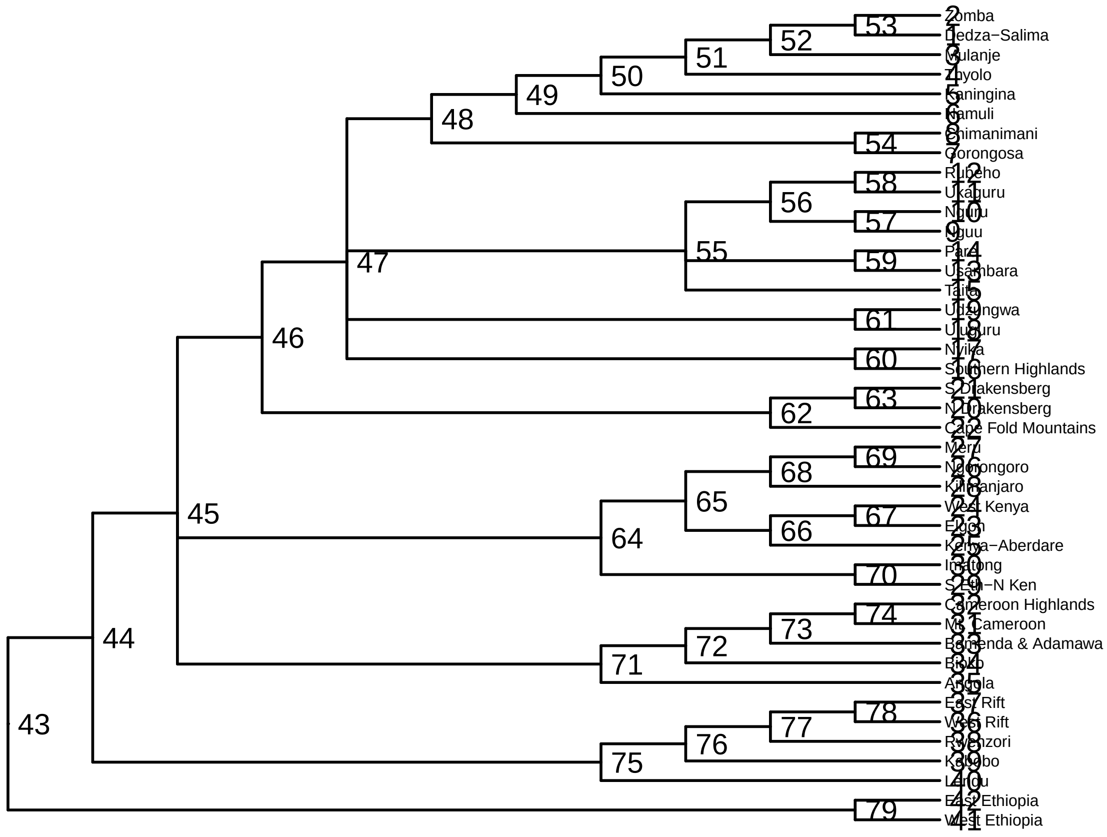

While we find 6 major regions to be ideal looking at the topology of the tree, and comparing with the estimated number of groups using the 'elbow method', and comparing these results to other biogeographic results.

Region	Node
Ethiopian Highlands	79
Lacustrine Rift	75
Cameroonian Highlands	71
Kenya-Tanzania Highlands	64
S Great Escarpment	62
Expanded Eastern Arc	47

```
offset=4

p2=ggtree(z,layout = "rectangular")+
  geom_tiplab(size=2)+
  scale_color_manual(values="black")+
  geom_cladelabel(node=79,align=T,offset=offset,color=x.colors[1],
                  label="Ethiopia")+
  geom_cladelabel(node=75,align=T,offset=offset,color=x.colors[2],
                  label="Lacustrine Rift")+
  geom_cladelabel(node=71,align=T,offset=offset,color=x.colors[3],
                  label="Cameroon Highlands & Angola")+
  geom_cladelabel(node=64,align=T,offset=offset,color=x.colors[4],
                  label="Kenya-Tanzania Highlands")+
```

```
  geom_cladelabel(node=62,align=T,offset=offset,color=x.colors[1],
                  label="S Great Escarpment")+
  geom_cladelabel(node=47,align=T,offset=offset,color=x.colors[2],
                  label="Expanded Eastern Arc")+
  #geom_cladelabel(node=75,align=T,offset=offset,color=x.colors[4],
  #                label="Cameroonian Highlands")+
  #geom_cladelabel(node=23,align=T,offset=offset,color=x.colors[1],
  #                label="Angola")+
  #geom_cladelabel(node=80,align=T,offset=offset,color=x.colors[2],
  #                label="Core Eastern Arc",fontsize=2.5)+
  #geom_cladelabel(node=92,align=T,offset=offset,color=x.colors[3],
  #                label="S Eastern Arc & Satellite Regions")+
  #geom_text(aes(label=node),hjust=-.3)
  expand_limits(x=c(0,25))#+
  #geom_nodepoint(color="black",
  #               size=(x2@phylo$node.label/25),
  #               shape="diamond")

print(p2)
```

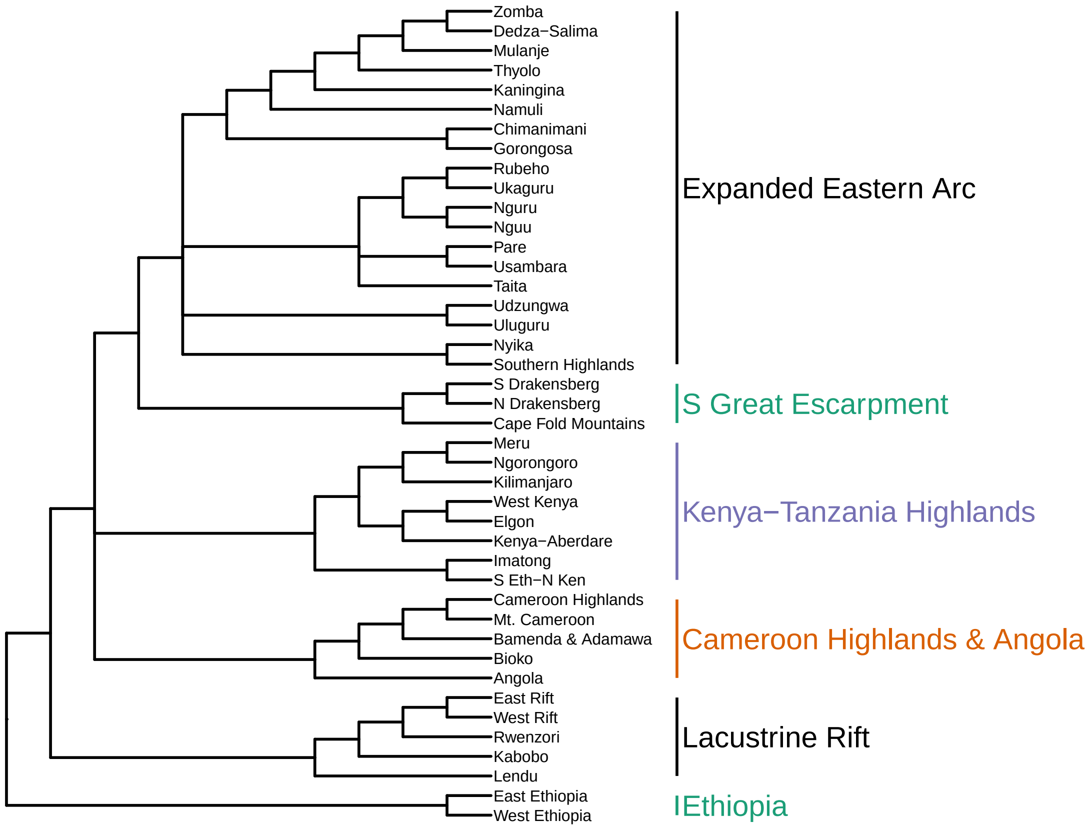


```
ggsave(p2,filename=paste0(output,"Cooper_consensus.png"),dpi=400)
```

```
## Saving 6.5 x 4.5 in image
```

We can also compare all consensuses.

```
c.trees=paste0(filepath,c.trees)

z=read.tree(c.trees[c.trees%like%"Cooper"])
y=read.tree(c.trees[c.trees%like%"Bowie"])
x=read.tree(c.trees[c.trees%like%"Dowsett"])

z$tip.label=gsub("_"," ",z$tip.label)
x$tip.label=gsub("_"," ",x$tip.label)
y$tip.label=gsub("_"," ",y$tip.label)

x=fortify(x)
y=fortify(y)
z=fortify(z)

y$x=y$x+max(x$x)+1
z$x=z$x+max(y$x)+1

dd=bind_rows(x,y,z)%>%
  filter(!is.na(label))

p1=ggtree(x,layout='rectangular')
p2=geom_tree(data=y)
p3=geom_tree(data=z)
p4=geom_tiplab(data=z,size=2)
p5=geom_line(aes(x,y,group=label),data=dd,alpha=.3,size=0.3)
p6=xlim(c(0,max(z$x)+5))
p7=ylim(c(-0.5,max(z$y)))

plot1=p1+p2+p3+p4+p5+p6+p7
```

```
## Scale for 'y' is already present. Adding another scale for 'y', which will
## replace the existing scale.
```

```
plot1
```

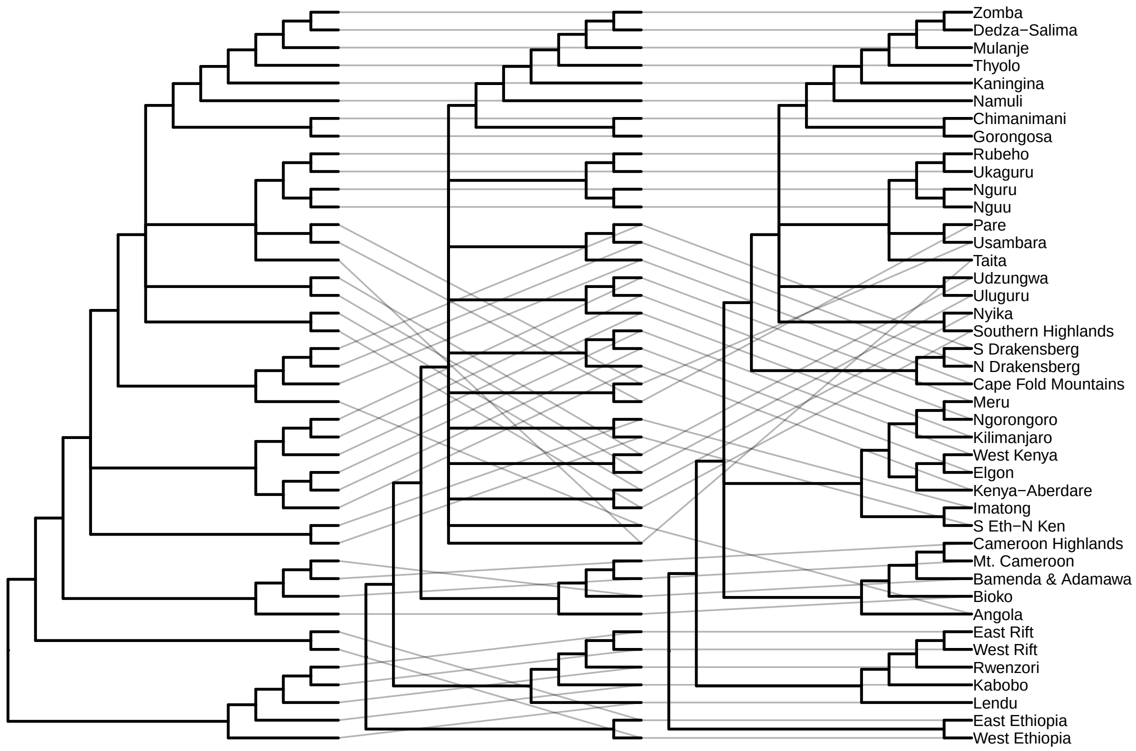


```
ggsave(paste0(filepath,"Consensus_compared.jpg"),
       plot = plot1,dpi = 400)
```

```
## Saving 6.5 x 4.5 in image
```

5.1 Introduction: Ecostructure Part I: Setup and Initial Plots

This exercise is designed to determine hypotheses of phylogenetic relationships between major biogeographic regions by means of assessing taxonomic relationships between all taxa known from these mountains. Taxa differ with regards to being described to Genera, species, and subspecies, and the hierarchical effects of these relationships sheds light on how similar populations are between different mountain ranges.

```
library(tidyverse)
library(ggplot2)
library(ecostructure)
library(data.table)
library(sf)
```

```
## Linking to GEOS 3.8.0, GDAL 3.0.4, PROJ 6.3.1
```

```
library(rnaturalearth)
library(grid)
library(gridExtra)
```

```
##
## Attaching package: 'gridExtra'
```

```
## The following object is masked from 'package:dplyr':
```

```
##
##     combine
```

```
x=as.data.frame(read_csv(paste0(filepath,"TableS1_v2.csv")))
```

```
## Rows: 732 Columns: 51
```

```
## -- Column specification --------------------------------------------------------
## Delimiter: ","
## chr  (8): From Bowie, From Dowsett, Exclude, Genus, Superspecies, Species, G...
## dbl (43): Clements, Bioko, Mt. Cameroon, Cameroon Highlands, Bamenda & Adama...
```

```
##
## i Use `spec()` to retrieve the full column specification for this data.
## i Specify the column types or set `show_col_types = FALSE` to quiet this message.
```

```
x[is.na(x)]=0
colSums(x[,-c(1:9)])
```

```
##                Bioko        Mt. Cameroon  Cameroon Highlands   Bamenda & Adamawa
##                   36                  50                  52                  64
##                Lendu          West Rift            Rwenzori           East Rift
##                   69                 132                  98                 120
##               Kabobo      West Ethiopia       East Ethiopia         S Eth-N Ken
##                   66                 103                  93                  34
##              Imatong               Elgon         West Kenya      Kenya-Aberdare
##                   63                  95                 108                 106
##           Ngorongoro                Meru         Kilimanjaro               Taita
##                   84                  84                  82                  43
##                 Pare            Usambara                Nguu               Nguru
##                   61                  81                  41                  46
##              Ukaguru              Rubeho             Uluguru            Udzungwa
##                   53                  51                  77                  88
##   Southern Highlands               Nyika          Kaningina        Dedza-Salima
##                   82                  81                  60                  48
##                Zomba              Thyolo             Mulanje              Namuli
##                   50                  50                  49                  41
##            Gorongosa         Chimanimani       N Drakensberg       S Drakensberg
##                   33                  54                  59                  68
## Cape Fold Mountains              Angola
##                   49                  59
```

```
colnames(x)
```

```
##  [1] "From Bowie"          "From Dowsett"        "Exclude"
##  [4] "Clements"            "Genus"               "Superspecies"
##  [7] "Species"             "Group"               "Subspecies"
## [10] "Bioko"               "Mt. Cameroon"        "Cameroon Highlands"
## [13] "Bamenda & Adamawa"   "Lendu"               "West Rift"
## [16] "Rwenzori"            "East Rift"           "Kabobo"
## [19] "West Ethiopia"       "East Ethiopia"       "S Eth-N Ken"
## [22] "Imatong"             "Elgon"               "West Kenya"
## [25] "Kenya-Aberdare"      "Ngorongoro"          "Meru"
## [28] "Kilimanjaro"         "Taita"               "Pare"
## [31] "Usambara"            "Nguu"                "Nguru"
## [34] "Ukaguru"             "Rubeho"              "Uluguru"
## [37] "Udzungwa"            "Southern Highlands"  "Nyika"
```

```
## [40] "Kaningina"           "Dedza-Salima"         "Zomba"
## [43] "Thyolo"              "Mulanje"              "Namuli"
## [46] "Gorongosa"           "Chimanimani"          "N Drakensberg"
## [49] "S Drakensberg"       "Cape Fold Mountains" "Angola"
```

```
x2=x

# reformat names to be hierarchical
# already done in previous document
# x2$Superspecies=paste(x2$Genus,x2$Superspecies)
# x2$Species=paste(x2$Superspecies,x2$Species)
# x2$Group=paste(x2$Species,x2$Group)
# x2$Subspecies=paste(x2$Group,x2$Subspecies)
```

```
x2$Exclude=as.factor(x2$Exclude)

data.x=x2%>%filter(Exclude=="0")%>%
  select(-`From Bowie`,-`From Dowsett`)
```

```
write_csv(data.x,paste0(filepath,"ecostructure_data.csv"))
```

```
data.x=read_csv(paste0(filepath,"ecostructure_data.csv"))
```

```
## Rows: 725 Columns: 49

## -- Column specification --------------------------------------------------------
## Delimiter: ","
## chr  (5): Genus, Superspecies, Species, Group, Subspecies
## dbl (44): Exclude, Clements, Bioko, Mt. Cameroon, Cameroon Highlands, Bamend...

##
## i Use `spec()` to retrieve the full column specification for this data.
## i Specify the column types or set `show_col_types = FALSE` to quiet this message.
```

Now, to set up the code that can iteratively perform ecostructure. First, to become more familiar with ecostructure.

```
# load metadata file
meta.x=read_csv(paste0(filepath,"locality_metadata_v2.csv"))
```

```
## Rows: 42 Columns: 6

## -- Column specification --------------------------------------------------------
## Delimiter: ","
## chr (3): Locality, Division, Note
## dbl (3): Longitude, Latitude, Elevation

##
## i Use `spec()` to retrieve the full column specification for this data.
## i Specify the column types or set `show_col_types = FALSE` to quiet this message.
```

```
head(meta.x)
```

```
## # A tibble: 6 x 6
##   Locality           Division  Longitude Latitude Elevation Note
##   <chr>              <chr>         <dbl>    <dbl>     <dbl> <chr>
## 1 Bioko              Central        8.7      3.5       3012 <NA>
## 2 Mt. Cameroon       Central        9.17     4.22      4040 <NA>
## 3 Cameroon Highlands Central        9.83     5.03      2411 <NA>
## 4 Bamenda & Adamawa  Central       10.5      6.2       3011 <NA>
```

```
## 5 Lendu              Albertine     30.9       2.01       2455 <NA>
## 6 West Rift          Albertine     28.7      -2.25       3475 Mt Kahuzi coordinat~
```

```r
# Plot montane centroids

plot(asp=1,meta.x[,3:4],pch=19)
```

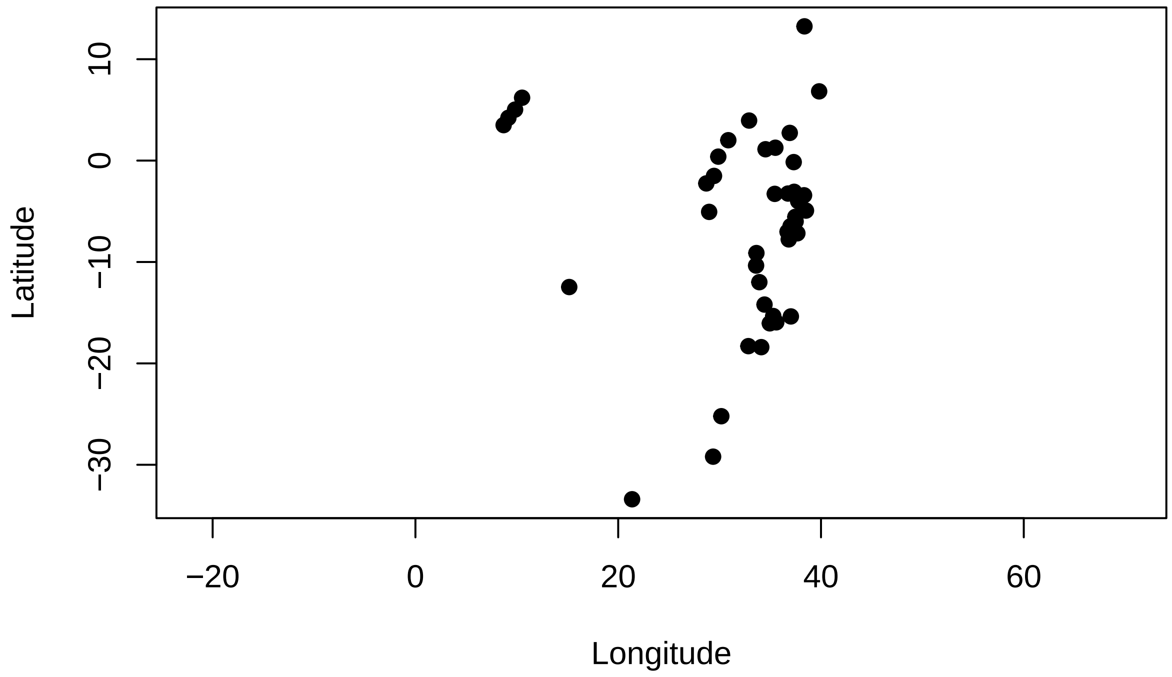


```r
set.seed(1000)
```

```r
#adjusted ecos_plot_mod

ecos_plot_mod = function(omega = NULL,
                         coords = NULL,
                         bgmap_path = NULL,
                         adjust = FALSE,
                         thresh = 0.7,
                         long_lim = c(-180,180),
                         lat_lim = c(-60,90),
                         coastline_lwd = 10,
                         intensity = 1,
                         radius = 0.5,
                         sea=NULL,
                         lake=NULL,
                         country=NULL,
                         color = c("dodgerblue2","#E31A1C", "green4",
                                   "#6A3D9A","#FF7F00", "black","gold1",
                                   "skyblue2","#FB9A99","palegreen2",
```

```
                                    "#CAB2D6", "#FDBF6F",  "gray70",
                                    "khaki2", "maroon","orchid1","deeppink1",
                                    "blue1","steelblue4","darkturquoise","green1"
                                    ,"yellow4","yellow3", "darkorange4","brown",
                                    "red", "cornflowerblue", "cyan", "brown4",
                                    "burlywood", "darkgoldenrod1","azure4",
                                    "green","deepskyblue","yellow", "azure1"),
                          pie_control = list(),
                          image_width = 1000,
                          image_height = 800,
                          path = "geostructure_plot.tiff"){

require(sf)
require(rnaturalearth)

if(is.null(coords)){
  if(is.null(rownames(omega))){
    stop("coords not provided, omega rownames do not have latitude longitude
         information either")
  }
  latlong_chars <- rownames(omega)
  coords <- cbind.data.frame(
    as.numeric(sapply(latlong_chars, function(x) strsplit(x, "_")[[1]][1])),
    as.numeric(sapply(latlong_chars, function(x) strsplit(x, "_")[[1]][2])))
  colnames(coords) <- c("lat", "long")
}else{
  if(dim(coords)[1] != dim(omega)[1]){
    stop("coords provided, but the number of rows in coords data does not
         match the number of rows in omega matrix")
  }
}

pie_control_default <- list(edges = 200, clockwise = TRUE,
                            init.angle = 90, density = NULL,
                            angle = 45, border = NULL,
                            lty = NULL, label.dist = 1.1)

pie_control <- modifyList(pie_control_default, pie_control)

if(is.null(sea)){
  print("Loading shapefile layers.")
  sea=ne_download(scale=110,type="ocean",category="physical")%>%
    st_as_sf()%>%st_geometry()
  lake=ne_download(scale=110,type="lakes",category="physical")%>%
    st_as_sf()%>%st_geometry()
  country=ne_download(scale=110,type="boundary_lines_land",
                      category="cultural")%>%
    st_as_sf()%>%st_geometry()
}else{
  print("Maps preloaded.")
}

#glob <- c(xmin=long_lim[1], xmax=long_lim[2], ymin=lat_lim[1], ymax=lat_lim[2])
```

```r
  #glob <- sf::st_bbox(glob)
  #glob <- structure(glob, crs = sf::st_crs(sea))
  #GlobalCoast <-suppressWarnings(suppressMessages(sf::st_intersection(GlobalCoast,
  #                                                                     sf::st_as_sfc(glob))))

  if(adjust){
    idx <- which(omega[,1] > thresh)
    omega <- omega[-idx,]
    coords <- coords[-idx,]
    omega <- omega[,-1]
    omega <- t(apply(omega, 1, function(x) return(x/sum(x))))
  }

  output_type <- strsplit(path, "[.]")[[1]][2]

  if(output_type == "tiff"){
    tiff(path, width = image_width, height = image_height)
  }else if(output_type == "png"){
    png(path, width = image_width, height = image_height)
  }else if(output_type == "pdf"){
    pdf(path, width = image_width, height = image_height)
  }else{
    stop("the output image may either be of  tiff, png or pdf extension")
  }

  plot(sea,col="#e1e1e1",axes=T,main="",lwd=coastline_lwd,
       xlim=long_lim,ylim=lat_lim)
  plot(country,add=T,lwd=0.5)
  plot(lake,col="#e1e1e1",axes=T,main="",lwd=coastline_lwd,add=T)

  par(lwd =.01)
  invisible(lapply(1:dim(omega)[1], function(r)
    do.call(mapplots::add.pie, append(list(
      z=as.integer(100*omega[r,]),
      x=coords[r,1],
      y=coords[r,2],
      labels=c("","",""),
      radius = radius,
      col=sapply(color, scales::alpha, intensity))
      , pie_control))))
  invisible(dev.off())
}
```

```r
# adjusted eco_block

ecos_blocks_mod=function(omega,
                         filepath,
                         level,
                         ncluster,
                         blocker_metadata,
                         order_metadata,
                         palette = c("#E69F00", "#56B4E9", "#009E73", "#F0E442",
                                     "#0072B2", "#D55E00", "#CC79A7"),
                         structure_control = list(),
```

```
                        layout){

  if(!is.factor(blocker_metadata)){
    stop("the blocker_metadata must be a factor variable")
    }
  if(!is.numeric(order_metadata)){
    stop("the order_metadata must be a numeric variable")
    }
  num_levels <- length(levels(blocker_metadata))
  if(missing(layout)){
    layout <- c(floor(sqrt(num_levels)), ceiling(sqrt(num_levels)))
    }
  structure_control_default <- list(split_line=list(split_lwd = 1,
                                                    split_col = "white"),
                                    axis_tick = list(axis_ticks_length = .1,
                                                     axis_ticks_lwd_y = .1,
                                                     axis_ticks_lwd_x = .1,
                                                     axis_label_size = 6,
                                                     axis_label_face = "bold"),
                                    plot_labels = TRUE,
                                    levels_decreasing=FALSE,
                                    order_sample=TRUE,
                                    round_off=1,
                                    panel_title_size=10,
                                    panel_title_font=4,
                                    main_title="Block Structure Plot",
                                    yaxis_label = "Locality")

  structure_control <- modifyList(structure_control_default, structure_control)

  split_indices <- split(1:dim(omega)[1], as.factor(blocker_metadata))
  split_struct <- list()

  for(l in 1:length(split_indices)){

    order_split <- round(order_metadata[split_indices[[l]]], structure_control$round_off);
    omega_split <- omega[split_indices[[l]],]
    if(structure_control$levels_decreasing){
      order_split_ordered <- order_split[order(order_split, decreasing=TRUE)]
      omega_split_ordered <- omega_split[order(order_split, decreasing=TRUE),]
    }else{
      order_split_ordered <- order_split[order(order_split, decreasing=FALSE)]
      omega_split_ordered <- omega_split[order(order_split, decreasing=FALSE),]
    }
    annotation <- data.frame(
      sample_id = paste0("X", c(1:NROW(omega_split_ordered))),
      tissue_label = factor(order_split_ordered,
                            levels = unique(order_split_ordered) ) );

    split_struct[[l]] <- CountClust::StructureGGplot(omega = omega_split_ordered,
                          annotation = annotation,
                          figure_title = names(split_indices)[l],
                          palette = palette,
```

```
                          yaxis_label = structure_control$yaxis_label,
                          split_line=structure_control$split_line,
                          order_sample = structure_control$order_sample,
                          axis_tick = structure_control$axis_tick,
                          plot_labels=structure_control$plot_labels)

  }

  plot.struct=do.call(gridExtra::grid.arrange,
                      args = list(grobs=split_struct,
                                  ncol = layout[2],
                                  nrow = layout[1],
                                  top=grid::textGrob(structure_control$main_title,
                                        gp=
                                          gpar(fontsize=
                                                 structure_control$panel_title_size,
                                               font=
                                                 structure_control$panel_title_font))))

  print(plot.struct)
  ggsave(filename=paste0(filepath,level,
                         '_',ncluster,'_','blocks_plot.png'),
         plot=plot.struct,
         dpi=400)
}
```

5.2 Genus assignment

This is the coarsest phylogenetic treatment. We will write a function that will perform the necessary analyses on our behalf.

```
# define variables just in case
# level is CASE SENSITIVE

division=as.factor(as.character(meta.x$Division))

coords.x=meta.x%>%select(Longitude,Latitude)%>%
  as.data.frame()
row.names(coords.x)=meta.x$Locality

eco_africa=function(level,ncluster=NULL,data.x,
                    tolerance=NULL,n.trials=NULL,coords.x=NA,
                    sea=NULL,lake=NULL,country=NULL){
  if(is.null(ncluster)==T){ncluster=2}
  if(is.null(tolerance)==T){tolerance=0.1}
  if(is.null(n.trials)==T){n.trials=10}

  palette.x=c('#a6cee3','#1f78b4',
              '#b2df8a','#33a02c',
              '#fb9a99','#e5e5e5',
              '#e31a1c','#fdbf6f',
              '#ff7f00','#cab2d6',
              '#6a3d9a','#ffff99',
```

```
              '#b15928','#000000')

x3=data.x%>%
  filter(Exclude!="Exclude")%>%
  select(-Clements,-Exclude)

# case sensitive, finds column match
xnames=x3[,which(colnames(x3)%flike%level)] %>% as.data.frame()

x4=x3%>%select(-Genus,-Superspecies,-Species,
               -Group,-Subspecies)%>%
  t()

colnames(x4)=xnames[,1]

u.names=unique(xnames)

for(i in 1:nrow(u.names)){
  target=u.names[i,1]
  if(sum(colnames(x4)==target)<=1){
    index=which(colnames(x4)==target)
    nu.x=x4[,c(index,index)]
    if(i==1){
      x6=as.data.frame(nu.x)[,1]
    }else{
      x6=cbind(x6,nu.x[,1])
    }
  }
  if(sum(colnames(x4)==target)>1){
    xx=x4[,which(colnames(x4)==target)]
    nu.x=rowSums(xx)
    nu.x[nu.x>1]=1
    if(i==1){
      x6=as.data.frame(cbind(nu.x,nu.x))
      x6=x6[,1]
    }else{
      x6=cbind(x6,nu.x)
    }
  }
}

colnames(x6)=u.names[,1]

fit=ecos_fit(x6,K=ncluster,
             tol=tolerance,num_trials=n.trials)

ord.x=1:nrow(fit$omega)

ecos_blocks_mod(fit$omega,blocker_metadata=as.factor('Afromontane'),
                order_metadata = ord.x,
                palette = palette.x,
                filepath=filepath,
                level=level,
```

```
                  ncluster=ncluster)

  # make maps

  features=CountClust::ExtractTopFeatures(fit$theta,
                                        top_features = 5,
                                        method="poisson",
                                        options="max")

  t(apply(features$indices,c(1,2),
          function(x){return(rownames(fit$theta)[x])}))

  # compare observed to null
  out=ecos_nullmodel(x6,K=ncluster,null.model = "richness",
                     iter_randomized = 10,option="BF")

  print(out)

  if(is.na(coords.x)==F){
    #ymin=min(coords.x$Latitude)+0.5
    #ymax=max(coords.x$Latitude)+0.5
    #xmin=min(coords.x$Longitude)+0.5
    #xmax=max(coords.x$Longitude)+0.5

    ecos_plot_mod(omega=fit$omega,
                  lat_lim=c(-42,14),
                  long_lim=c(-20,60),
                  coords=coords.x,
                  path=paste0(filepath,level,
                              '_',ncluster,'_','geostructure_plot.png'),
                  color = palette.x,
                  coastline_lwd = 2,
                  sea=sea,lake=lake,country=country)
  }

  if(is.na(coords.x)==F){
    #ymin=min(coords.x$Latitude)+0.5
    #ymax=max(coords.x$Latitude)+0.5
    #xmin=min(coords.x$Longitude)+0.5
    #xmax=max(coords.x$Longitude)+0.5

    ecos_plot_mod(omega=fit$omega,
                  lat_lim=c(-14,3),
                  long_lim=c(30,42),
                  coords=coords.x,
                  path=paste0(filepath,level,
                              '_',ncluster,'_','subset_geostructure_plot.png'),
                  color = palette.x,
                  coastline_lwd = 2,
                  sea=sea,lake=lake,country=country)
  }
}
```

K = 2

Load maps the first time.

```
# the following loads maps from online
# as of 6 Sep 2021, this was not working
# could not resolve URL
# see workaround below
# note from 13 Oct 2021:
# appeared to be issue with dependency 'rnaturalearthhires'
# package best loaded separately

sea=ne_download(scale=110,type="ocean",category="physical")%>%
  st_as_sf()%>%st_geometry()
lake=ne_download(scale=110,type="lakes",category="physical")%>%
  st_as_sf()%>%st_geometry()
country=ne_download(scale=110,type="boundary_lines_land",
                    category="cultural")%>%
  st_as_sf()%>%st_geometry()
```

```
library(rgdal)
```

```
## Loading required package: sp

## Please note that rgdal will be retired by the end of 2023,
## plan transition to sf/stars/terra functions using GDAL and PROJ
## at your earliest convenience.
##
## rgdal: version: 1.5-27, (SVN revision 1148)
## Geospatial Data Abstraction Library extensions to R successfully loaded
## Loaded GDAL runtime: GDAL 3.0.4, released 2020/01/28
## Path to GDAL shared files: /usr/share/gdal
## GDAL binary built with GEOS: TRUE
## Loaded PROJ runtime: Rel. 6.3.1, February 10th, 2020, [PJ_VERSION: 631]
## Path to PROJ shared files: /usr/share/proj
## Linking to sp version:1.4-5
## To mute warnings of possible GDAL/OSR exportToProj4() degradation,
## use options("rgdal_show_exportToProj4_warnings"="none") before loading sp or rgdal.
```

```
# download from local shapefiles
# save in a directory referenced as 'GIS'

sea=readOGR(paste0(GIS,
                   'ne_10m_ocean/',
                   'ne_10m_ocean.shp'))%>%
  st_as_sf()%>%st_geometry()
```

```
## OGR data source with driver: ESRI Shapefile
## Source: "/home/j141c380/Dropbox/GIS/ne_10m_ocean/ne_10m_ocean.shp", layer: "ne_10m_ocean"
## with 1 features
## It has 3 fields
```

```
lake=readOGR(paste0(GIS,
                    'ne_10m_lakes/',
                    'ne_10m_lakes.shp'))%>%
  st_as_sf()%>%st_geometry()
```

```
## OGR data source with driver: ESRI Shapefile
## Source: "/home/j141c380/Dropbox/GIS/ne_10m_lakes/ne_10m_lakes.shp", layer: "ne_10m_lakes"
## with 1354 features
## It has 37 fields
## Integer64 fields read as strings:  scalerank ne_id
```

```
country=readOGR(paste0(GIS,
                       'ne_10m_admin_0_boundary_lines_land/',
                       'ne_10m_admin_0_boundary_lines_land.shp'))%>%
  st_as_sf()%>%st_geometry()
```

```
## OGR data source with driver: ESRI Shapefile
## Source: "/home/j141c380/Dropbox/GIS/ne_10m_admin_0_boundary_lines_land/ne_10m_admin_0_boundary_lines_
## with 462 features
## It has 18 fields
## Integer64 fields read as strings:  scalerank
```

```
eco_africa(level="Genus",ncluster = 2,
           data.x=data.x,coords.x=coords.x,
           sea=sea,lake=lake,country=country)
```

K = 3

```
eco_africa(level="Genus",ncluster = 3,
           data.x=data.x,coords.x=coords.x,sea=sea,lake=lake,country=country)
```

K = 4

```
eco_africa(level="Genus",ncluster = 4,
           data.x=data.x,coords.x=coords.x,sea=sea,lake=lake,country=country)
```

K = 5

```
eco_africa(level="Genus",ncluster = 5,
           data.x=data.x,coords.x=coords.x,sea=sea,lake=lake,country=country)
```

K = 6

```
eco_africa(level="Genus",ncluster = 6,
           data.x=data.x,coords.x=coords.x,sea=sea,lake=lake,country=country)
```

K = 7

```
eco_africa(level="Genus",ncluster = 7,
           data.x=data.x,coords.x=coords.x,sea=sea,lake=lake,country=country)
```

K = 8

```
eco_africa(level="Genus",ncluster = 8,
           data.x=data.x,coords.x=coords.x,sea=sea,lake=lake,country=country)
```

K = 9

```
eco_africa(level="Genus",ncluster = 9,
           data.x=data.x,coords.x=coords.x,sea=sea,lake=lake,country=country)
```

K = 10

```
eco_africa(level="Genus",ncluster = 10,
           data.x=data.x,coords.x=coords.x,sea=sea,lake=lake,country=country)
```

K = 11

```
eco_africa(level="Genus",ncluster = 11,
           data.x=data.x,coords.x=coords.x,sea=sea,lake=lake,country=country)
```

K = 12

```
eco_africa(level="Genus",ncluster = 12,
           data.x=data.x,coords.x=coords.x,sea=sea,lake=lake,country=country)
```

K = 13

```
eco_africa(level="Genus",ncluster = 13,
           data.x=data.x,coords.x=coords.x,sea=sea,lake=lake,country=country)
```

K = 14

```
eco_africa(level="Genus",ncluster = 14,
           data.x=data.x,coords.x=coords.x,sea=sea,lake=lake,country=country)
```

6.1 Introduction: Ecostructure Part II

This is a setup for how other levels of *ecostructure* were called. **Note** that I do not include calls for every level of *ecostructure* in the documentation here, but they are performed using the same functions etc.

6.2 Code Setup

```
library(tidyverse)
library(ggplot2)
library(ecostructure)
library(data.table)
library(sf)
library(rnaturalearth)
library(grid)
library(gridExtra)

source(paste0(filepath,"ecostructure_mod_codes.R"))
```

```
# the following loads maps from online
# as of 6 Sep 2021, this was not working
```

```
# could not resolve URL
# see workaround below

sea=ne_download(scale=110,type="ocean",category="physical")%>%
  st_as_sf()%>%st_geometry()
lake=ne_download(scale=110,type="lakes",category="physical")%>%
  st_as_sf()%>%st_geometry()
country=ne_download(scale=110,type="boundary_lines_land",
                    category="cultural")%>%
  st_as_sf()%>%st_geometry()
```

```
library(rgdal)

# download from local shapefiles
# save in a directory referenced as 'GIS'

sea=readOGR(paste0(GIS,
                   'ne_10m_ocean/',
                   'ne_10m_ocean.shp'))%>%
  st_as_sf()%>%st_geometry()
```

```
## OGR data source with driver: ESRI Shapefile
## Source: "/home/j141c380/Dropbox/GIS/ne_10m_ocean/ne_10m_ocean.shp", layer: "ne_10m_ocean"
## with 1 features
## It has 3 fields
```

```
lake=readOGR(paste0(GIS,
                    'ne_10m_lakes/',
                    'ne_10m_lakes.shp'))%>%
  st_as_sf()%>%st_geometry()
```

```
## OGR data source with driver: ESRI Shapefile
## Source: "/home/j141c380/Dropbox/GIS/ne_10m_lakes/ne_10m_lakes.shp", layer: "ne_10m_lakes"
## with 1354 features
## It has 37 fields
## Integer64 fields read as strings:  scalerank ne_id
```

```
country=readOGR(paste0(GIS,
                       'ne_10m_admin_0_boundary_lines_land/',
                       'ne_10m_admin_0_boundary_lines_land.shp'))%>%
  st_as_sf()%>%st_geometry()
```

```
## OGR data source with driver: ESRI Shapefile
## Source: "/home/j141c380/Dropbox/GIS/ne_10m_admin_0_boundary_lines_land/ne_10m_admin_0_boundary_lines_
## with 462 features
## It has 18 fields
## Integer64 fields read as strings:  scalerank
```

```
data.x=read_csv(paste0(filepath,"ecostructure_data.csv"))
```

```
## Rows: 725 Columns: 49
```

```
## -- Column specification --------------------------------------------------------
## Delimiter: ","
## chr  (5): Genus, Superspecies, Species, Group, Subspecies
## dbl (44): Exclude, Clements, Bioko, Mt. Cameroon, Cameroon Highlands, Bamend...
```

```
##
## i Use `spec()` to retrieve the full column specification for this data.
## i Specify the column types or set `show_col_types = FALSE` to quiet this message.
```

```
# load metadata file
meta.x=read_csv(paste0(filepath,"locality_metadata_v2.csv"))
```

```
## Rows: 42 Columns: 6
```

```
## -- Column specification --------------------------------------------------------
## Delimiter: ","
## chr (3): Locality, Division, Note
## dbl (3): Longitude, Latitude, Elevation
```

```
##
## i Use `spec()` to retrieve the full column specification for this data.
## i Specify the column types or set `show_col_types = FALSE` to quiet this message.
```

```
coords.x=meta.x%>%select(Longitude,Latitude)%>%
  as.data.frame()
row.names(coords.x)=meta.x$Locality

set.seed(1000)
```

6.3 Superspecies assignment

This is the coarsest phylogenetic treatment. We will write a function that will perform the necessary analyses on our behalf.

K = 2

```
eco_africa(level="Superspecies",ncluster = 2,
           data.x=data.x,coords.x=coords.x,sea=sea,lake=lake,country=country)
```

K = 3

```
eco_africa(level="Superspecies",ncluster = 3,
           data.x=data.x,coords.x=coords.x,sea=sea,lake=lake,country=country)
```

K = 4

```
eco_africa(level="Superspecies",ncluster = 4,
           data.x=data.x,coords.x=coords.x,sea=sea,lake=lake,country=country)
```

K = 5

```
eco_africa(level="Superspecies",ncluster = 5,
           data.x=data.x,coords.x=coords.x,sea=sea,lake=lake,country=country)
```

K = 6

```
eco_africa(level="Superspecies",ncluster = 6,
           data.x=data.x,coords.x=coords.x,sea=sea,lake=lake,country=country)
```

K = 7

```
eco_africa(level="Superspecies",ncluster = 7,
           data.x=data.x,coords.x=coords.x,sea=sea,lake=lake,country=country)
```

K = 8

```
eco_africa(level="Superspecies",ncluster = 8,
           data.x=data.x,coords.x=coords.x,sea=sea,lake=lake,country=country)
```

K = 9

```
eco_africa(level="Superspecies",ncluster = 9,
           data.x=data.x,coords.x=coords.x,sea=sea,lake=lake,country=country)
```

K = 10

```
eco_africa(level="Superspecies",ncluster = 10,
           data.x=data.x,coords.x=coords.x,sea=sea,lake=lake,country=country)
```

K = 11

```
eco_africa(level="Superspecies",ncluster = 11,
           data.x=data.x,coords.x=coords.x,sea=sea,lake=lake,country=country)
```

K = 12

```
eco_africa(level="Superspecies",ncluster = 12,
           data.x=data.x,coords.x=coords.x,sea=sea,lake=lake,country=country)
```

K = 13

```
eco_africa(level="Superspecies",ncluster = 13,
           data.x=data.x,coords.x=coords.x,sea=sea,lake=lake,country=country)
```

K = 14

```
eco_africa(level="Superspecies",ncluster = 14,
           data.x=data.x,coords.x=coords.x,sea=sea,lake=lake,country=country)
```
